# Large breathing effect in ZIF-65(Zn) with expansion and contraction of the SOD cage

**DOI:** 10.1038/s41467-022-32332-x

**Published:** 2022-08-05

**Authors:** Meizhen Gao, Rui-Kang Huang, Bin Zheng, Pengfei Wang, Qi Shi, Wei-Xiong Zhang, Jinxiang Dong

**Affiliations:** 1 College of Chemistry and Chemical Engineering, Taiyuan University of Technology, Taiyuan, 030024 Shanxi China; 2 MOE Key Laboratory of Bioinorganic and Synthetic Chemistry, School of Chemistry, Sun Yat-Sen University, Guangzhou, 510275 Guangdong China; 3 School of Materials Science and Engineering, Xiʹan University of Science and Technology, Xiʹan, 710054 Shaanxi China; 4 State Key Laboratory of Coal Conversion, Institute of Coal Chemistry, Chinese Academy of Sciences, Taiyuan, 030001 Shanxi China

**Keywords:** Metal-organic frameworks, Solid-state chemistry

## Abstract

The flexibility and guest-responsive behavior of some metal-organic frameworks (MOFs) indicate their potential in the fields of sensors and molecular recognition. As a subfamily of MOFs, the flexible zeolitic imidazolate frameworks (ZIFs) typically feature a small displacive transition due to the rigid zeolite topology. Herein, an atypical reversible displacive transition (6.4 Å) is observed for the sodalite (SOD) cage in flexible ZIF-65(Zn), which represents an unusually large breathing effect compared to other ZIFs. ZIF-65(Zn) exhibits a stepwise II → III → I expansion between an unusual ellipsoidal SOD cage (8.6 Å × 15.9 Å for II) and a spherical SOD cage (15.0 Å for I). The breathing behavior of ZIF-65(Zn) varies depending on the nature of the guest molecules (polarity and shape). Computational simulations are employed to rationalize the differences in the breathing behavior depending on the structure of the ZIF-65(Zn) cage and the nature of the guest-associated host–guest and guest–guest interactions.

## Abstract (web summary)

Flexible metal-organic frameworks have potential applications in the development of sensors and switching materials. Here, the authors report a large breathing effect in a zeolitic imidazolate framework upon guest adsorption.

## Introduction

Flexibility is a unique property of metal-organic frameworks (MOFs), which differentiates them from rigid inorganic porous materials, such as zeolites^1^. Flexibility is associated with reversible movements, such as gate-opening and/or breathing effects, which require an external stimulus^2^. The flexible effects based on host–guest interactions suggest the immense potential of flexible frameworks in the fields of sensors, molecular recognition, adsorption, and separation^2^. The pioneering works on flexible MOFs were reported by Férey’s^3^ and Kitagawa’s^4,5^ groups. Thereafter, extensive research activities on the synthesis and applications of flexible MOFs have emerged^6–13^.

Zeolitic imidazolate frameworks (ZIFs)^14–17^ are a subclass of MOFs^18^, which consist of tetrahedral metal units linked by imidazole (Im) ligands and have also been reported and referred to as metal azolate frameworks^19,20^, tetrahedral imidazolate frameworks^21,22^, boron imidazolate frameworks^23,24^. ZIFs usually have zeolite topologies as the Im coordination angle in ZIFs is similar to the Si–O–Si angle in zeolites, which were originally considered to be rigid like zeolite.

ZIF-8 [Zn(mIm)_2_, mIm = 2-methylimidazole] is a prototypical ZIF with a SOD topology^14^, which is generally considered as a rigid MOF. However, under extreme pressure^25^ and low temperature^26^, ZIF-8 retains its space group symmetry, but displays gate-opening or swing effect phenomena, which involves the rotation of the mIm linker and the expansion of the pore windows. In addition, some reports have examined the effect of functional groups on the structural transition using ZIF-8 isoreticular frameworks^27,28^. The flexibility of the ZIF-8 lattice was observed upon the application of an electric field^29^ or low-frequency terahertz irradiation^30^, which was attributed to the linker movement and the change in the bond angles. Similar to ZIF-8, COK-17^31^, EMM-19^32^, and EMM-36^33^ with the same SOD topology also display structural flexibility.

Compared with ZIF-8, ZIF-7 [Zn(bIm)_2_, bIm = benzimidazole] with the same SOD topology displays pronounced gate-opening or breathing effects under conventional conditions, such as room temperature and relatively low pressure^34–45^. After the removal of the guests in ZIF-7, a narrow-pore structure was obtained. A large-pore structure was obtained upon the adsorption of the guests, which was derived from the rotation of the linker and the change in the bond angles. In addition, an extra-large pore structure can be observed under CO_2_ at cryogenic conditions. Noted that the crystal structure of the extra-large pore ZIF-7 was not determined^42,43^. Moreover, Long et al. examined the effects of metal substitution on the structural transitions using the isoreticular series of the ZIF-7 family (ZIF-9 and CdIF-13)^46^. In addition, a reversible transition in ZIFs has been extensively studied between the crystal and amorphous/disorder phases^47,48^.

Therefore, among the ~250 ZIFs structures reported to date^49^, the above-mentioned ZIFs exhibit gate-opening or breathing effects upon the adsorption–desorption of guest molecules. In addition, ZIF-8 and ZIF-7 have the same SOD topology and basic composite building units (SOD cage), which feature a reversible minimal and medium change in the size and pore volume of the SOD cage, respectively.

Replacement of the −CH_3_ group on ZIF-8 with −NO_2_ groups leads to isostructural ZIF-65(Zn) [Zn(nIm)_2_, nIm = 2-nitroimidazole]. Banerjee et al. first reported the synthesis and structure of ZIF-65 which was formed by connecting the Co ions via the nIm ligands^15,50^. Furthermore, the zinc counterpart, ZIF-65(Zn) [also named NOF-1, ZIF-108 or α-ZIF-65(Zn)] has been reported^51–57^. ZIF-65(Zn) has generally been synthesized using dimethylformamide (DMF) as the solvent that ultimately fills the pore space. In most practical applications, the activation of the as-synthesized ZIF-65(Zn) is necessary to fully open the pore space. Noteworthily, the ZIF-65(Zn) crystal usually turned into an unknown structure after activation upon removal of the guests^53,54,56,57^, but can be recovered when the activated ZIF-65(Zn) is immersed in the reaction solvent^57^. Although the powder X-ray diffraction (PXRD) and N_2_ sorption results provide a preliminary experimental description of the structural flexibility of ZIF-65(Zn), the crystal structure of the activated ZIF-65(Zn) has not been determined. The challenge is to understand and predict the occurrence of structural transitions in flexible materials, which is imperative for their use in any practical application.

In this work, we report the structural characterization of the activated ZIF-65(Zn) and a systematic study of the guest-responsive reversible structural transition of flexible ZIF-65(Zn). ZIF-65(Zn) exhibits a substantial expansion and contraction between its ellipsoidal and spherical SOD cage, which is associated with the adsorption and desorption of guest molecules with specific polarity/shape. The reversible structural transition of ZIF-65(Zn) is studied in detail and compared to other flexible ZIFs. To the best of our knowledge, the drastic contraction and expansion of the SOD cage and very large breathing effect (6.4 Å) reported herein for ZIF-65(Zn) are uncommon^25,29,39,44,57^.

## Results

### Structural analysis of ZIF-65(Zn)

The original ZIF-65(Zn) [labeled as ZIF-65(Zn)-I] was prepared using DMF as the reaction solvent, which ultimately fills the pore space. It is generally believed that the structure of ZIF-65(Zn)-I turned into an unknown structure [labeled as ZIF-65(Zn)-II] after activation upon the removal of guests^53,54,56,57^. The relatively weak polarity ethanol is an effective exchange solvent and the corresponding slow solvent release strategy can activate the ZIF-65(Zn)-I without structural transition, which is described in detail in the Supplementary Information. Thus, ZIF-65(Zn)-I cannot be recovered by immersing the ZIF-65(Zn)-II sample in exchange-solvent ethanol, but was recovered in reaction solvent DMF.

**Fig. 1:**
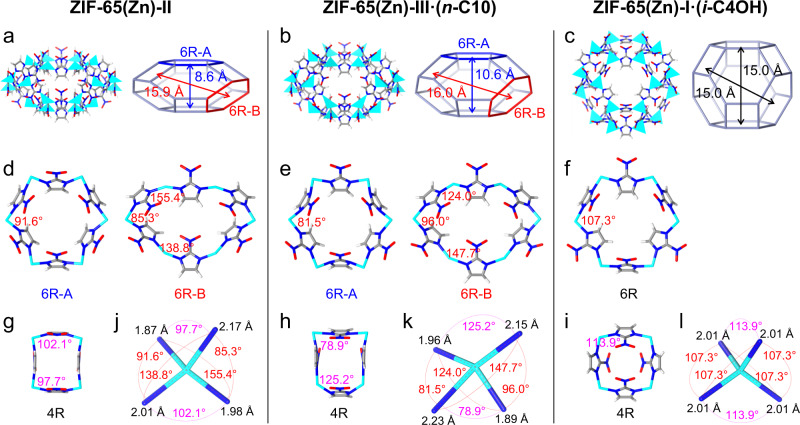
The structure of ZIF-65(Zn)-II, ZIF-65(Zn)-III·(*n*-C10), and ZIF-65(Zn)-I·(*i*-C4OH). Polyhedron representation and topological net of the SOD cage and cage size based on the distance between the face-to-face six-membered rings (6R) planes (**a**–**c**). The SOD cage of ZIF-65(Zn)-II and ZIF-65(Zn)-III·(*n*-C10) with two different 6R (the ratio of 6R-A and 6R-B is 1:3) is ellipsoidal, while the SOD cage of ZIF-65(Zn)-I·(*i*-C4OH) with one unique 6R is spherical. The face-to-face 6R planes are 8.6 Å × 15.9 Å, 10.6 Å × 16.0 Å, and 15.0 Å × 15.0 Å for ZIF-65(Zn)-II, ZIF-65(Zn)-III·(*n*-C10), and ZIF-65(Zn)-I·(*i*-C4OH), respectively. Six-membered rings (6R) and corresponding N–Zn–N bond angle (red) (**d**–**f**). Compared with the normal N–Zn–N bond angle (107.3°) in ZIF-65(Zn)-I·(*i*-C4OH), three N–Zn–N bond angles of 6R-B in ZIF-65(Zn)-II and ZIF-65(Zn)-III·(*n*-C10) are strongly twisted. Four-membered rings (4R) and corresponding N–Zn–N bond angle (magenta) (**g**–**i**). All N–Zn–N bond angles and Zn–N bond lengths (black) (**j**–**l**). Compared with the Zn–N bond length (2.01 Å) in ZIF-65(Zn)-I·(*i*-C4OH), the Zn–N bond length in ZIF-65(Zn)-II and ZIF-65(Zn)-III·(*n*-C10) has a more dispersive distribution. Zn: cyan; C: gray; N: blue; O: red; H: white. [Note: *n*-decane (*n*-C10), isobutanol (*i*-C4OH).].

To investigate the reversible structural transition between ZIF-65(Zn)-II and ZIF-65(Zn)-I, the crystal structure of the ZIF-65(Zn)-II should be determined. Since the single crystal of ZIF-65(Zn)-II was poor, its structural analysis had to rely on higher-quality X-ray powder diffraction. In addition, the intermediate phase (labeled as ZIF-65(Zn)-III) can be obtained in the structural transition process from the contraction phase ZIF-65(Zn)-II to the expansion phase ZIF-65(Zn)-I. Details of the structure model and Rietveld refinement for ZIF-65(Zn) are shown in the Methods section. The final Rietveld plots reveal a very good agreement between the calculated and experimental PXRD profiles (Supplementary Figs. 7–11). ZIF-65(Zn)-II, ZIF-65(Zn)-III, and ZIF-65(Zn)-I have identical SOD topologies and the basic composite building units SOD cage (Fig. 1a–c, Supplementary Fig. 12). The SOD cage whose surface is defined by six 4-rings (4R) and eight 6-rings (6R). The cage parameter (the distance between the face-to-face 6R planes) is used to analyze the cage expansion and contraction for ZIF-65(Zn). In addition, the N–Zn–N angles and Zn–N bond lengths between different ZIF-65(Zn) phases are also compared.

ZIF-65(Zn)-II and ZIF-65(Zn)-III have the trigonal crystal system, but contain an unusual ellipsoidal SOD cage which is rare in ZIFs (Fig. 1a, b). The spherical SOD cage of ZIF-65(Zn)-I resemble those of other known cubic ZIFs (ZIF-8 and ZIF-90). The cage of ZIF-65(Zn)-II and ZIF-65(Zn)-III has two different 6R (the ratio of 6R-A and 6R-B is 1:3) and the distances between the face-to-face 6R planes are 8.6 Å × 15.9 Å for ZIF-65(Zn)-II and 10.6 Å × 16.0 Å for ZIF-65(Zn)-III. While the cage of ZIF-65(Zn)-I have one unique 6R and the distance between the face-to-face 6R planes was estimated to be 15.0 Å. The drastic displacive transition (6.4 Å) in the SOD cage (8.6 Å for II, 10.6 Å for III, and 15.0 Å for I) is indicative of the very large breathing effect of the ZIF-65(Zn) framework. During the transition of classical ZIF-7-II^44^ to ZIF-7-I^14^, the adsorption of the guest molecules induces a small swelling of the SOD cage (11.3 Å × 16.1 Å–14.3 Å × 15.8 Å, Supplementary Fig. 14). ZIFs reported to date typically feature a small displacive transition due to the rigid zeolite topology^25,29,39,44,57^ (Supplementary Fig. 14 and Supplementary Table 5). ZIF-65(Zn) provides an example of ZIFs exhibiting a very large breathing effect (6.4 Å) without any change in the topology. Noted that MIL-88D^58^ represents the typical example of the large breathing effect in MOFs and the displacive transition of triangular bipyramid cages is close to 10 Å (Supplementary Fig. 15).

The expansion and contraction of the SOD cage are mainly due to the N–Zn–N bond angle of the ring (Fig. 1d–f). It should be noted that the standard Im–M–Im angle in ZIFs and O–Si–O angle in zeolites are close to 109.5°. In ZIF-65(Zn)-I, one unique 6R in the SOD cage has the normal N–Zn–N angle (107.3°). In ZIF-65(Zn)-II, there are two kinds of 6R with strongly different conformations, the conformation of 6R-A has an N–Zn–N angle of 91.6°, which resembles that of ZIF-65(Zn)-I; while the conformation of 6R-B is less symmetric and has three strongly twisted N–Zn–N angles (85.3°, 138.8°, and 155.4°). The dramatic change in the N–Zn–N angle (48.1°) between ZIF-65(Zn)-II and ZIF-65(Zn)-I leads to a large displacive transition (6.4 Å) of the SOD cage. In contrast, there is no link in the equatorial plane of the triangular bipyramid cages for MIL-88D^58^, and the distance between the three metal trimers in the equatorial plane can vary without constraints, which is essential for the large displacive transition (Supplementary Fig. 15). The drastic change in the size/shape of the SOD cage and the corresponding N–Zn–N angle has not been previously observed in the known ZIFs^25,29,39,44,57^ (Supplementary Fig. 14, Supplementary Tables 5, 6). The N–Zn–N angle in ZIF-65(Zn)-III is strongly twisted and resembles that of ZIF-65(Zn)-II, which will not be described in detail. Compared with the Zn–N bond length 2.01 Å in ZIF-65(Zn)-I, the Zn–N distance in ZIF-65(Zn)-II has a more dispersive distribution, lying in the range of 1.87–2.17 Å (Fig. 1j, l and Supplementary Table 3). The significant difference between bond lengths also plays an important role in the large displacive transition of the ZIF-65(Zn) SOD cage.

### The liquid adsorption of ZIF-65(Zn)

To investigate the guest-responsive structural transition of ZIF-65(Zn), the ZIF-65(Zn)-II solid was soaked in various polar/nonpolar and linear/branched solvents.

**Fig. 2:**
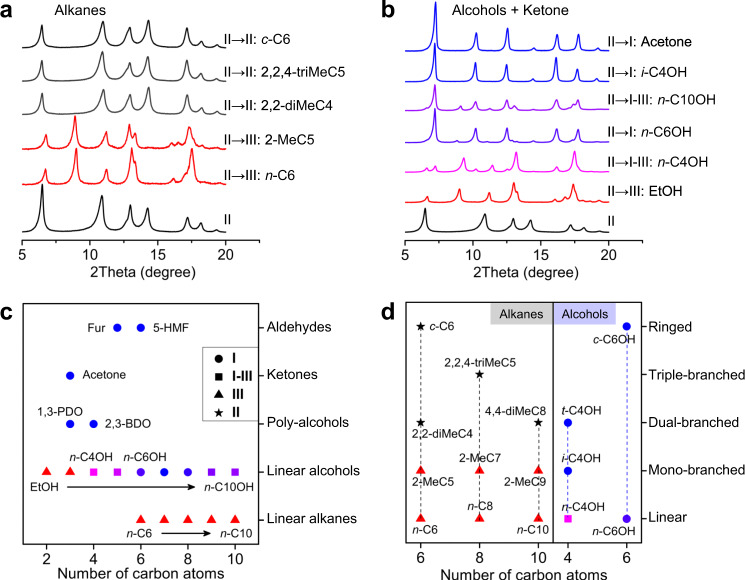
Liquid adsorption of ZIF-65(Zn)-II. PXRD of ZIF-65(Zn)-II after immersion in typical **a** alkanes, **b** alcohols and ketones at 298 K. The distribution map of the guest-responsive structural transition depended on **c** the polarity of guest molecules: non-polar (linear alkanes), polar (linear alcohols) and more polar (poly-alcohols, aldehydes and ketones) molecules, **d** the shape of guest molecules: linear, mono-branched, dual-branched, triple-branched and ringed molecules (alkanes and alcohols). [Note: *n*-hexane (*n*-C6), *n*-octane (*n*-C8), *n*-decane (*n*-C10), 2-methylpentane (2-MeC5), 2-methylheptane (2-MeC7), 2-methylnonane (2-MeC9), 2,2-dimethylbutane (2,2-diMeC4), 2,2,4-trimethylpentane (2,2,4-triMeC5), 4,4-dimethyloctane (4,4-diMeC8), cyclohexane (*c*-C6), ethanol (EtOH), *n*-butanol (*n*-C4OH), *n*-hexanol (*n*-C6OH), *n*-decanol (*n*-C10OH), isobutanol (*i*-C4OH), tert-butanol (*t*-C4OH), cyclohexanol (*c*-C6OH), 1,3-propanediol (1,3-PDO), 2,3-butanediol (2,3-BDO), furfural (Fur), 5-hydroxymethylfurfural (5-HMF).].

The degree of structural transition of ZIF-65(Zn) is selective. Noted that the molecule polarizability increases gradually as the number of carbon atoms increases. Depending on the polarity (dipole moment and polarizability) of the solvent molecules (Supplementary Table 7), different degrees of structural transition are observed in Fig. 2c and Supplementary Fig. 16: (i) Non-polar linear alkanes [*n*-hexane (*n*-C6) and *n*-decane (*n*-C10)] induce a small swelling of the SOD cage due to weak interactions. ZIF-65(Zn)-II (8.6 Å × 15.9 Å) transforms into ZIF-65(Zn)-III (10.6 Å × 16.0 Å) and the expansion displacive transition is increased by 2.0 Å. (ii) Notably, a threshold of the expansion magnitude exists for polar linear alcohols. ZIF-65(Zn)-III and I are obtained using short linear alcohols [ethanol (EtOH) and *n*-propanol (*n*-C3OH)] and long linear alcohols [*n*-hexanol (*n*-C6OH) and *n*-octanol (*n*-C8OH)], respectively, and a mixture phase of III and I is obtained for middle linear alcohols [*n*-butanol (*n*-C4OH) and *n*-pentanol (*n*-C5OH)]. As the number of carbon atoms increases, the linear alcohol molecule contains more aliphatic sites, and the host–guest and guest–guest interactions increase gradually, which induces a stepwise expansion of the SOD cage. Noted that a mixture phase of I with a small amount of III is obtained for more long linear alcohols [*n*-decanol (*n*-C10OH)], which suggests that the guest–guest interactions in ZIF-65(Zn) are weak due to the steric effects of the more long linear alcohols in the confined cage. (iii) More polar poly-alcohols [1,3-propanediol (1,3-PDO)], aldehydes [furfural (Fur)] and ketones (acetone) generate a large expansion of the SOD cage due to strong interactions. ZIF-65(Zn)-II (8.6 Å × 15.9 Å) transforms into ZIF-65(Zn)-I (15.0 Å) and the expansion displacive transition reaches 6.4 Å.

Depending on the size and shape (degree of the branch) of the solvent molecules, different degrees of structural transition are also observed in Fig. 2d and Supplementary Fig. 16: (i) Linear and mono-branched alkanes induce a small swelling from II to III. However, no structural transition can be observed upon the increasing degree of branching in the alkanes when using dual- and triple-branched alkanes as the solvent, which is attributed to a combination of the steric effects and weak interactions in the confined cage. TG results (Supplementary Fig. 17a) also show that the cage can only accommodate one dual/triple-branched alkane molecule, which can generate only weak interactions. (ii) The mixture phase of III and I is obtained using the linear *n*-C4OH. In contrast, the immersion of ZIF-65(Zn)-II in branched isobutanol (*i*-C4OH) leads to the complete transition to phase I. The results indicate that the branch of the alkyl groups for polar alcohols considerably strengthens the host–guest and guest–guest interactions in the confined cage.

Thus, the degree of structural transition and expansion magnitude of ZIF-65(Zn) is selective and responsive depending on the polarity and shape of the guest molecules. Similarly, depending on the chemical nature of guest molecules (polarity and shape), three degrees of pore opening for MIL-88C are also evidenced^58^. The main difference between the expansion of ZIF-65(Zn) and MIL-88C is the position and types of host–guest interactions. In MIL-88C, guest molecules can interact with both the inorganic metal trimers (coordination of the metal, H-bonding interactions) and organic linkers (van der Waals (vdW), CH-π, and π-π interactions). While in ZIF-65(Zn), guest molecules can only interact with the organic linkers (vdW and H-bonding interactions). The reason for such dissimilarities in the expansion magnitude can be observed in the characteristic of the ZIF-65(Zn) cage and the nature of the guest-associated host–guest and guest–guest interactions^58^, which will be discussed below using computational calculations.

### The vapor and gas adsorption of ZIF-65(Zn)

To further explore the breathing behavior of ZIF-65(Zn) depending on the polarity and shape of the guest molecules, the vapor adsorption-desorption isotherms of ZIF-65(Zn)-II for the representative alkanes, alcohols, aldehydes, and ketones are shown in Fig. 3a–e and Supplementary Figs. 18–29. For comparison, the isotherms of ZIF-65(Zn)-I were also studied. In addition, the dynamic reversible structural transitions of ZIF-65(Zn) in the vapor adsorption-desorption processes were monitored using in situ PXRD in Fig. 4b–d and Supplementary Figs. 30–40.

**Fig. 3:**
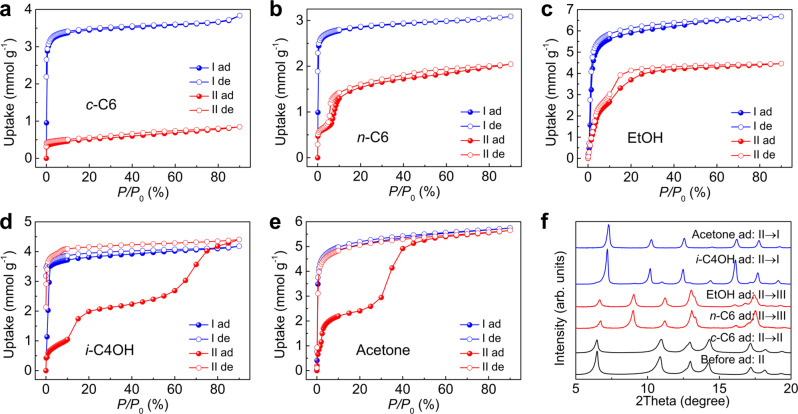
The vapor adsorption isotherms in ZIF-65(Zn) and corresponding PXRD patterns. Comparison of the adsorption (solid) and desorption (empty) isotherms of **a** *c*-C6, **b** *n*-C6, **c** EtOH, **d** *i*-C4OH, and **e** acetone in ZIF-65(Zn)-II (red) and ZIF-65(Zn)-I (blue) at 298 K, respectively. **f** PXRD patterns of ZIF-65(Zn)-II before and after *c*-C6, *n*-C6, EtOH, *i*-C4OH, and acetone adsorption (*P*/*P*_0_ = 90%). [Note: cyclohexane (*c*-C6), *n*-hexane (*n*-C6), ethanol (EtOH), isobutanol (*i*-C4OH), adsorption (ad), desorption (de).].

**Fig. 4:**
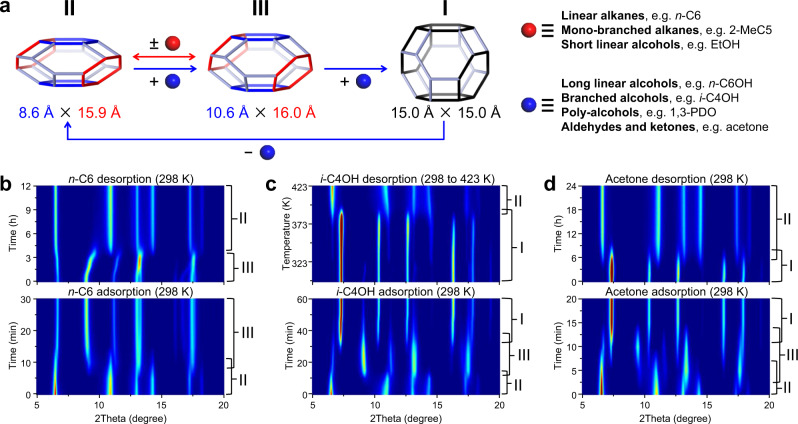
The breathing behavior of ZIF-65(Zn). **a** The breathing behavior and expansion magnitude of ZIF-65(Zn) is selective and responsive depending on the nature of the guest molecules. In situ PXRD patterns for ZIF-65(Zn)-II measured during the adsorption and desorption of **b** *n*-C6 (II ↔ III at 298 K), **c** *i*-C4OH (II → III → I at 298 K, I → II from 298 to 423 K), and **d** acetone (II → III → I at 298 K, I → II at 298 K), respectively. [Note: *n*-hexane (*n*-C6), 2-methylpentane (2-MeC5), ethanol (EtOH), *n*-hexanol (*n*-C6OH), isobutanol (*i*-C4OH), 1,3-propanediol (1,3-PDO).].

The cyclohexane (*c*-C6) isotherms of ZIF-65(Zn)-II shows no step in the accessible pressure range (Fig. 3a), and in situ PXRD reveals that the ZIF-65(Zn) framework remains in II when *c*-C6 adsorption took place (Supplementary Fig. 30), which is attributed to a combination of the steric effects and weak interactions for ring conformation *c*-C6 in the confined ZIF-65(Zn)-II cage. In contrast, the linear molecule *n*-C6 isotherms of ZIF-65(Zn)-II exhibit a significant pre-step uptake, followed by a steep adsorption-desorption step with hysteresis loops (Fig. 3b), which implies a reversible transition of ZIF-65(Zn). In situ PXRD in the adsorption-desorption processes further confirms the reversible II ↔ III structural transition (Fig. 4b). The *n*-C7 isotherms of ZIF-65(Zn)-II also exhibit a similar step to the *n*-C6 isotherms, but *n*-C8, *n*-C9, and *n*-C10 isotherms of ZIF-65(Zn)-II show somewhat less distinct stepwise adsorption (Supplementary Figs. 20–23), which can be attributed to their steric effects. EtOH isotherms of ZIF-65(Zn)-II show fewer distinct steps but feature a hysteresis loop (Fig. 3c), *n*-C3OH and *n*-C4OH isotherms show both a distinct step and hysteresis loop (Supplementary Figs. 25, 26), which can be ascribed to the reversible II ↔ III structural transition (Supplementary Figs. 36–38).

The *i*-C4OH isotherms of ZIF-65(Zn)-II exhibit a significant pre-step uptake, followed by two well-defined stepping uptake phenomena (Fig. 3d), which suggest the existence of two distinct structural transitions. The observed adsorption behavior can be confirmed using in situ PXRD, which can be ascribed to II → III and III → I structural transitions, respectively (Fig. 4c). The adsorption amounts of *i*-C4OH on ZIF-65(Zn)-II and ZIF-65(Zn)-I almost coincide at *P*/*P*_0_ = 90%, which also indicates the II → I structural transition. However, no sharp step is present in the *i*-C4OH desorption branch and ZIF-65(Zn) remains in phase I. It is noted that the inverse I → II transition will occur at 398 K in the desorption processes (Fig. 4c). A similar two steps adsorption is observed in the case of the more polar acetone vapor, which can be ascribed to II → III and III → I structural transitions, respectively (Fig. 3e). Compared to the *i*-C4OH desorption branch of the isotherms, a fairly gradual decrease in the adsorbed acetone with a larger hysteresis is visible during the desorption step. The acetone desorption behavior using in situ PXRD can confidently confirm the inverse I → II transition (Fig. 4d). Unfortunately, the inverse I → III transition was not observed.

Thus, the adsorption and desorption of linear alkanes and short linear alcohols in ZIF-65(Zn)-II correspond to the reversible II ↔ III structural transition, which induces a small expansion and contraction of the SOD cage (2.0 Å). While, the adsorption and desorption of branched molecule *i*-C4OH and more polar acetone in ZIF-65(Zn)-II correspond to the reversible II ↔ I structural transition, which induces a large expansion and contraction of the SOD cage (6.4 Å).

**Fig. 5:**
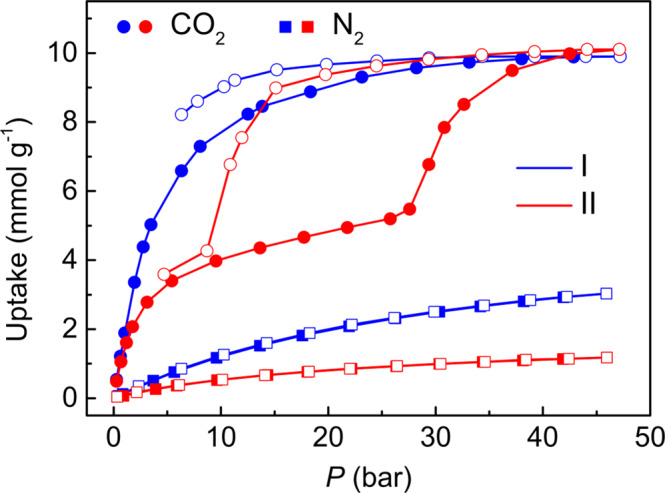
Gas adsorption of ZIF-65(Zn). Comparison of the adsorption (solid) and desorption (empty) isotherms of CO_2_ (circles) and N_2_ (squares) in ZIF-65(Zn)-II (red) and ZIF-65(Zn)-I (blue) at 298 K, respectively.

Furthermore, high-pressure adsorption-desorption isotherms with CO_2_ and N_2_ were carried out at 298 K (Fig. 5). N_2_ isotherms of ZIF-65(Zn)-II show low uptake and no step in the accessible pressure range, which indicate no structural transition occurs. CO_2_ isotherms of ZIF-65(Zn)-II exhibit pre-step adsorption of 3.4 mmol g^–1^ at 5.4 bar and the step-shaped adsorption from 5.5 mmol g^–1^ (27.6 bar) to 10.1 mmol g^–1^ (47.1 bar) and a large hysteresis loop, which hint at CO_2_ may induce structural transition. In addition, CO_2_ adsorption amounts on II and I almost coincide at 47.1 bar, which indicates the II → I structural transition. This transition is brought about by more polar CO_2_ but not by N_2_, which implies the potential application of ZIF-65(Zn) in separation.

### The dynamic structural transition of ZIF-65(Zn)

**Fig. 6:**
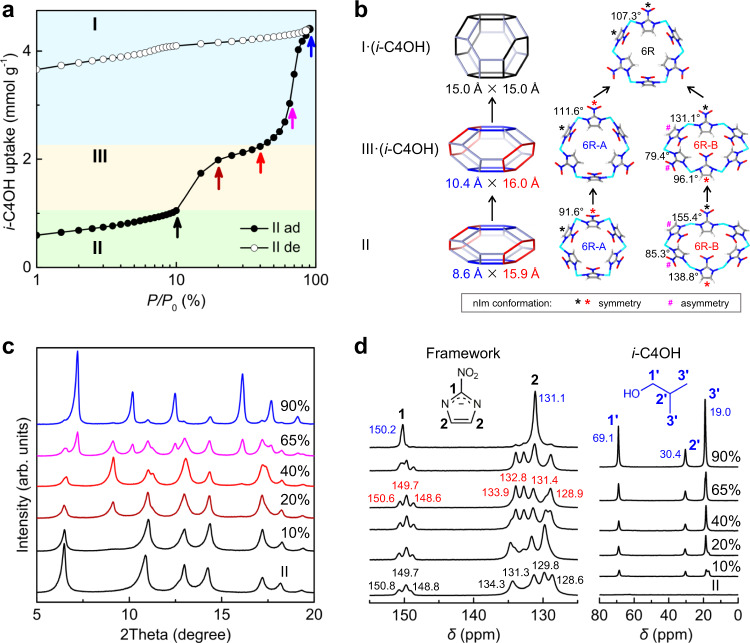
The dynamic structural transition for *i*-C4OH adsorption in ZIF-65(Zn)-II. **a** The *i*-C4OH adsorption (solid) and desorption (empty) logarithmic isotherms at 298 K. **b** The change of SOD cage, nIm conformation, and N–Zn–N bond angle in six-membered rings (6R) during II → III·(*i*-C4OH) → I·(*i*-C4OH) structural transitions. **c** The PXRD patterns and **d** ^13^C NMR spectra upon increasing the *i*-C4OH relative pressure. [Note: isobutanol (*i*-C4OH), adsorption (ad), desorption (de).].

To directly observe the structural transitions in ZIF-65(Zn)-II upon the adsorption of the guest molecules, we conducted PXRD and ^13^C NMR measurements under the same conditions as the adsorption isotherms measurements. The representative *i*-C4OH (II → III → I) adsorption and corresponding structural transitions will be discussed below (Fig. 6). The *c*-C6 (II → II), *n*-C6 (II → III), and *n*-C4OH (II → III) adsorption will be discussed in Supplementary Information (Supplementary Fig. 41).

Remarkably, the *i*-C4OH adsorption in ZIF-65(Zn)-II proceeds in one pre-step and two distinct steps of adsorption (Fig. 6a). ZIF-65(Zn)-II shows an initial *i*-C4OH uptake below 10% (*P*/*P*_0_) and remains II from PXRD observation (Fig. 6c), in which the adsorption mainly occurs in the pore volume of II. The adsorption isotherm shows a sudden increase between 10 and 20% (*P*/*P*_0_) and maintains a slow smooth increase between 20 and 40% (*P*/*P*_0_), which corresponds to the II → III structural transition from PXRD observation, and the adsorption mainly occurs in the pore volume of III. From the ^13^C NMR spectrum of II and III·(*i*-C4OH) (*P*/*P*_0_ = 40%) (Fig. 6d), C#1 and C#2 of the nIm linker both correspond to three and four peaks, respectively, which are consistent with the three conformations of the nIm linker in II and III (Fig. 6b, Supplementary Fig. 13, and Supplementary Table 4). For C#2 of nIm linker in III·(*i*-C4OH), two peaks (*δ* = 131.4/128.9 ppm) are similar to those in II, but another two peaks (*δ* = 133.9/132.8 ppm) appear with the disappearance of another two peaks (*δ* = 134.3/129.8 ppm) in II. The most important difference is found that the N–Zn–N angle decreases from 155.4/138.8° (II) to 131.1/96.1° (III·(*i*-C4OH)) (Fig. 6b) corresponding to the displacement of nIm, which leads to the structural transition. [Note: the ^13^C NMR spectrum of III·(*i*-C4OH) is similar to that of III·(*n*-C4OH) in Supplementary Fig. 41. The structure of III·(*i*-C4OH) can refer to that of III·(*n*-C4OH)].

After reaching *P*/*P*_0_ = 55% (Supplementary Fig. S41a), the *i*-C4OH adsorption exhibits an abrupt increase and final uptake of cal. 4.4 mmol g^–1^ at *P*/*P*_0_ = 90%, which corresponds to the III → I structural transition from PXRD observation (Fig. 6c), and the adsorption mainly occurs in the pore volume of I. The ^13^C NMR spectrum of I·(*i*-C4OH) (*P*/*P*_0_ = 90%) exhibits one peak in C#1 and C#2 of the nIm linker, at *δ* = 150.2 and 131.1 ppm, respectively, which is consistent with the structure of ZIF-65(Zn)-I containing one unique nIm linker (Fig. 6b). The transition of multiple conformations of nIm linkers into one conformation further demonstrates the III → I structural transition.

### Understanding the flexibility of ZIF-65(Zn)

**Fig. 7:**
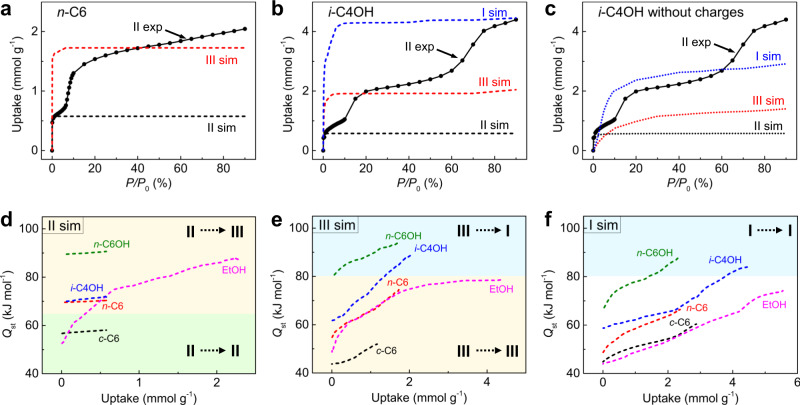
GCMC simulations. **a** Comparison of the simulated adsorption isotherms (dashed line) for *n*-C6 in II and III of ZIF-65(Zn) with the first and second plateau on the experimental adsorption isotherms (solid line) of ZIF-65(Zn)-II at 298 K. Comparison of the simulated adsorption isotherms (dashed line) for **b** *i*-C4OH with charges and **c** *i*-C4OH without charges in II, III, and I of ZIF-65(Zn) with the first, second, and third plateau on the experimental adsorption isotherms (solid line) of ZIF-65(Zn)-II at 298 K. The simulated adsorption heats (dashed line) of *c*-C6, *n*-C6, EtOH, *i*-C4OH and *n*-C6OH in **d** II, **e** III, and **f** I of ZIF-65(Zn), and the corresponding threshold in the guest adsorption heat above which breathing occurs. [Note: cyclohexane (*c*-C6), *n*-hexane (*n*-C6), ethanol (EtOH), isobutanol (*i*-C4OH), *n*-hexanol (*n*-C6OH).].

Theoretical calculations were performed to gain insight into the flexibility of ZIF-65(Zn) demonstrated in our experiments. Five guests with representative polarity/shape were selected to simulate the adsorption isotherms using grand canonical Monte Carlo (GCMC) methods: *c*-C6, *n*-C6, EtOH, *i*-C4OH, and *n*-C6OH (Fig. 7a, b and Supplementary Figs. 52–56). For the ringed alkane *c*-C6 adsorption (Supplementary Fig. 52), the simulated isotherm of ZIF-65(Zn)-II matches the corresponding experimental data well, which further indicates that no structural transition can be observed. For the linear alkane *n*-C6 (Fig. 7a) and short linear alcohol EtOH adsorption (Supplementary Fig. 54), the simulated isotherms of ZIF-65(Zn)-II and ZIF-65(Zn)-III broadly correspond to the first and second plateau on the experimental isotherm of ZIF-65(Zn)-II, respectively, which further indicates the distinct II → III structural transition. For the branched alcohol *i*-C4OH adsorption (Fig. 7b), the simulated isotherms of ZIF-65(Zn)-II, ZIF-65(Zn)-III, and ZIF-65(Zn)-I correspond broadly to the first, second and third plateau on the experimental isotherm of ZIF-65(Zn)-II, which further indicates the stepwise II → III → I structural transition.

We also utilized an osmotic ensemble model^59^ to predict structural transitions of ZIF-65(Zn) induced by *c*-C6, *n*-C6, EtOH, and *i*-C4OH adsorption, respectively (Supplementary Fig. 57). From the Langmuir isotherms fitted on experimental or simulated adsorption, we can plot a function of pressure for each phase to calculate the free energy difference between two phases and then corresponding to the osmotic potential. The switch of osmotic potential difference (∆Ω) between two phases is used to evaluate structural transition. ∆Ω = 0, which means that is the structural transition point. It can be seen that *c*-C6 does not induce structural transition and II is thermodynamically favored throughout the pressure range. Contrarily, *n*-C6 and EtOH induce II → III structural transition, and *i*-C4OH induces II → III → I structural transition.

To explain why ZIF-65(Zn) undergoes different breathing phenomena, the guest adsorption heat (${Q}_{{{{{{\rm{st}}}}}}}$) was calculated using GCMC simulations. It can simply be mentioned here that the structural transition relates to a threshold in the guest adsorption heat above which breathing occurs^60^. Combining the guest adsorption heat and structural transition results, we can define that the thresholds of the guest adsorption heat for II → III and III → I transition are 65 and 80 kJ mol^–1^, respectively (Fig. 7d–f). The *n*-C6 and EtOH adsorption heats (${Q}_{{{{{{\rm{st}}}}}}}^{{{\max}}}$) at the maximum adsorption capacity in II are 70.4 and 87.6 kJ mol^–1^, respectively, which induce a small swelling from II to III. Furthermore, the ${{Q}}_{{{{{{\mathrm{st}}}}}}}^{{{\max}}}$ of *i*-C4OH and *n*-C6OH in III are 88.9 and 93.9 kJ mol^–1^, respectively, which induce a large expansion from III to I. The simulated guest adsorption heats (${Q}_{{{{{{\rm{st}}}}}}}^{0}$) at infinite dilution in II, III, and I all show an increase upon increasing the polarity and size of the guest molecules due to an increase in the host–guest interactions in the confined cage. In addition, the ${Q}_{{{{{{\rm{st}}}}}}}$ in II, III, and I all show an increase with increasing uptake and the increase is even greater for the adsorption of guest molecules with larger size and polarity, which can be mainly attributed to an increase in the guest–guest interactions in the confined cage.

In addition, the host energy difference (∆${E}_{{{{{{\rm{f}}}}}}}$) between different ZIF-65(Zn) phases was evaluated by density functional theory (DFT) calculations^41^. For the empty ZIF-65(Zn) structure with 18·Zn(nIm)_2_, ∆${E}_{{{{{{\rm{f}}}}}}}$(II → III) and ∆${E}_{{{{{{\rm{f}}}}}}}$(III → I) are around 112.3 and 299.5 kJ mol^–1^, respectively. The guest adsorption heat difference (∆${Q}_{{{{{{\rm{st}}}}}}}$) between different ZIF-65(Zn) phases in the transition region was obtained from GCMC simulations. To compensate for the energy penalty of the structural transition, the ∆${Q}_{{{{{{\rm{st}}}}}}}$ needs to be higher than the energy criterion ∆${E}_{{{{{{\rm{f}}}}}}}$. This energy criterions are supported by the following examples (Supplementary Fig. 58 and Supplementary Table 15): the ∆${Q}_{{{{{{\rm{st}}}}}}}$(II → III) of *c*-C6 (108.0 kJ mol^–1^) is lower than the ∆${E}_{{{{{{\rm{f}}}}}}}$(II → III), meaning that II is maintained; while, the ∆${Q}_{{{{{{\rm{st}}}}}}}$(II → III) of *n*-C6, EtOH, *i*-C4OH and *n*-C6OH are higher than the ∆${E}_{{{{{{\rm{f}}}}}}}$(II → III), which induce a small swelling from II to III. Furthermore, the ∆${Q}_{{{{{{\rm{st}}}}}}}$(III → I) of *n*-C6 and EtOH (137.6 and 200.1 kJ mol^–1^, respectively) are lower than the ∆${E}_{{{{{{\rm{f}}}}}}}$(III → I), which do not induce further structural transition; while, the ∆${Q}_{{{{{{\rm{st}}}}}}}$(III → I) of *i*-C4OH and *n*-C6OH are higher than the ∆${E}_{{{{{{\rm{f}}}}}}}$(III → I), which induce a large expansion from III to I. Noted that the comparison results between ∆${E}_{{{{{{\rm{f}}}}}}}$ and ∆${Q}_{{{{{{\rm{st}}}}}}}$ confirm that the II → III phase transition cannot be observed in the adsorption of *c*-C6, but predict the occurrence of III → I phase transition. The results indicate that the steric effect of the ringed *c*-C6 in the confined cage of ZIF-65(Zn)-III is weaker than that of ZIF-65(Zn)-II and the cage of ZIF-65(Zn)-III can accommodate more guests, which may strengthen the guest–guest interactions and lead to the III → I phase transition.

To understand the nature of the host–guest and guest–guest interactions of the *i*-C4OH adsorption in ZIF-65(Zn), we simulated 3 different isotherms of *i*-C4OH adsorption in phases II, III, and I (Fig. 7b, c and Supplementary Fig. 59). Notably, ZIF-65(Zn) contains polar −NO_2_ groups that can form hydrogen (H) bonds with *i*-C4OH, and H-bonds can also be formed between the *i*-C4OH molecules. Simulation 1 is based on ZIF-65(Zn) and *i*-C4OH both with charges (Fig. 7b): the vdW interactions, host–guest H-bonding and guest–guest H-bonding interactions are considered; simulation 2 is based on ZIF-65(Zn) without charges and *i*-C4OH with charges (Supplementary Fig. 59): the vdW and guest–guest H-bonding interactions are considered; simulation 3 based on ZIF-65(Zn) with charges and *i*-C4OH without charges (Fig. 7c): the vdW interactions are considered. For the *i*-C4OH adsorption in II, III, and I, only the maximum adsorption capacity (${N}_{{{\max}}}$) of simulation 1 is close to the experimental adsorption value. Thus, we proposed that the synergistic effect of the vdW, host–guest H-bonding and guest–guest H-bonding interactions formed during the *i*-C4OH adsorption in ZIF-65(Zn) ultimately leads to the stepwise II → III → I structural transition.

**Fig. 8:**
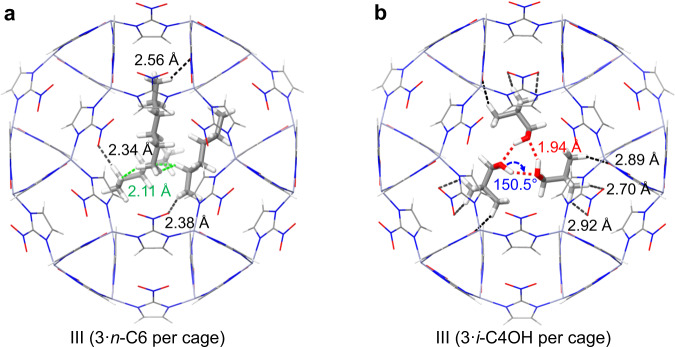
MC and DFTB calculated host–guest structures. Most probable **a** *n*-C6 and **b** *i*-C4OH adsorption site in ZIF-65(Zn)-III with the maximum number of adsorbed molecules per cage (3 *n*-C6 molecules per cage, or 3 *i*-C4OH molecules per cage). The host frameworks and guest molecules are shown as thin and thick stick models, respectively. Zn: slate gray; C: gray; N: blue; O: red; H: white. The strong guest–guest O–H···O H-bonding, weak host–guest C–H···O H-bonding, and guest–guest vdW interactions are displayed as red, black, and green dashed lines, respectively. [Note: *n*-hexane (*n*-C6), isobutanol (*i*-C4OH).].

To further determine the host–guest and guest–guest H-bonding interactions, the configurations of *n*-C6 and *i*-C4OH adsorption in ZIF-65(Zn) from the Monte Carlo (MC) simulations were optimized by the density-functional tight-binding (DFTB) calculations (Fig. 8 and Supplementary Figs. 60–66). In II, *n*-C6 and *i*-C4OH can form weak C–H···O and strong O–H···O H-bonds with the −NO_2_ groups, respectively (Supplementary Figs. 60, 61). Thus, *n*-C6 and *i*-C4OH lead to a small swelling from II to III due to the host–guest H-bonding interactions. In III, there are only weak C–H···O H-bonds formed between *n*-C6 and −NO_2_ groups (Fig. 8a and Supplementary Fig. 62); while, the –OH groups of three *i*-C4OH molecules interact head-to-tail with each other to form a triangle H-bond loop (bond length: 1.94–1.96 Å and bond angle: 149.9–150.5°) along the three-fold axis of the crystal, simultaneously, the –CH groups of three *i*-C4OH molecules further form regular C–H···O H-bonds with the −NO_2_ group (Fig. 8b and Supplementary Fig. 63). Thus, *i*-C4OH can lead to a further expansion from III to I due to the synergistic effect of the host–guest and guest–guest H-bonding interactions.

## Discussion

We have reported the structural characterization of flexible ZIF-65(Zn) and performed a systematic study of its guest-responsive reversible structural transition. ZIF-65(Zn)-II and ZIF-65(Zn)-III contain an unusual ellipsoidal SOD cage (8.6 Å × 15.9 Å for II and 10.6 Å × 16.0 Å for III), which is rare in ZIFs; ZIF-65(Zn)-I contain a spherical SOD cage (15.0 Å). ZIF-65(Zn) exhibits a stepwise II (contraction phase) →III (intermediate phase) →I (expansion phase) structural transition upon the adsorption of guest molecules. An inverse I → II transition will occur upon the desorption of the guest molecules. ZIF-65(Zn) represents a drastic expansion and contraction of the SOD cage (6.4 Å) and a corresponding dramatic change in the N–Zn–N angle (48.1°) without any change of the topology, which is an unusually large breathing effect compared to other ZIFs.

No structural transition can be observed in the adsorption and desorption of di/tri-branched alkanes in ZIF-65(Zn)-II. The adsorption and desorption of mono-branched alkanes, linear alkanes, and short linear alcohols in ZIF-65(Zn)-II correspond to a reversible II ↔ III structural transition, which induces a small expansion and contraction of the SOD cage. The adsorption and desorption of long linear alcohols, branched alcohols, and more polar poly-alcohols, aldehydes, and ketones in ZIF-65(Zn)-II correspond to a reversible II ↔ I structural transition, which induces a large expansion and contraction of the SOD cage. This II ↔ I structural transition is also brought about by more polar CO_2_ but not by N_2_ in the high pressure, which implies the potential application of ZIF-65(Zn) in separation. The breathing behavior and expansion magnitude of ZIF-65(Zn) depends on the nature of the guest molecules (polarity and shape), which has been further rationalized thanks to computational simulations. Non-polar linear molecule *n*-C6 leads to a small swelling from II to III due to the host–guest H-bonding interactions. In contrast, polar branched molecule *i*-C4OH leads to a large expansion from II to I due to the synergistic effect of the host–guest and guest–guest H-bonding interactions. We believe that this discovery will open up new avenues for the development of flexible ZIFs materials for a wide range of applications.

## Methods

### Synthesis of ZIF-65(Zn)

ZIF-65(Zn) was synthesized using a modification of the method published by Yaghi et al.^15^. Zinc acetate dehydrate [0.110 g, 0.5 mmol] and 2-nitroimidazole (nIm) [0.141 g, 1.25 mmol] were added to dimethylformamide (DMF) [15 mL] in a 30 mL Teflon-lined autoclave and heated at 100 °C for 48 h. The as-synthesized sample was labeled as ZIF-65(Zn)-I.

### Activation of ZIF-65(Zn)

Methanol exchange and activation: the as-synthesized sample was immersed in methanol (MeOH) for 24 h, changing the solvent with fresh MeOH at 12 h intervals. The exchanged sample was further activated by heating at 50 °C for 12 h or vacuuming at 150 °C for 12 h, which was labeled as ZIF-65(Zn)-II. Ethanol exchange and activation: the as-synthesized sample was immersed in ethanol (EtOH) for 36 h, changing the solvent with fresh EtOH at 12 h intervals. The exchanged sample was further activated by vacuuming at 50 °C for 1 h, which was also labeled as ZIF-65(Zn)-I.

### Preparation of different ZIF-65(Zn) phases

ZIF-65(Zn)-II was obtained via MeOH exchange and activation of the as-synthesized sample. ZIF-65(Zn)-III can be obtained by adsorbing specific guest molecules in ZIF-65(Zn)-II. Typically, ZIF-65(Zn)-III·(*n*-C10) was formed by immersing the ZIF-65(Zn)-II sample in *n*-C10. ZIF-65(Zn)-III·(*n*-C4OH) was formed via *n*-C4OH vapor adsorption in ZIF-65(Zn)-II. ZIF-65(Zn)-I (Cubic *I*-43*m*) was synthesized using DMF as the reaction solvent. ZIF-65(Zn)-I (Cubic *I*-43*m*) can also be obtained by adsorbing specific guest molecules in ZIF-65(Zn)-II. Typically, ZIF-65(Zn)-I·(*i*-C4OH) was obtained by immersing the ZIF-65(Zn)-II sample in *i*-C4OH. ZIF-65(Zn)-I (Cubic *P*-43*m*) was obtained via EtOH exchange and activation of the as-synthesized sample.

### Structural model and Rietveld refinement of ZIF-65(Zn)

The PXRD data obtained for structural refinement was collected on a Bruker D8 Advance diffractometer equipped with Cu Kα radiation (*λ* = 1.5418 Å) at 40 kV and 40 mA, in the 2*θ* range of 5–80° with a scan step size of 0.02° and 4 s per step. The indexing and refinement of the PXRD patterns were carried out using the Reflex module of Materials Studio 8.0^61^. The patterns of ZIF-65(Zn)-II, ZIF-65(Zn)-III·(*n*-C10), ZIF-65(Zn)-III·(*n*-C4OH), ZIF-65(Zn)-I·(*i*-C4OH) and ZIF-65(Zn)-I were well indexed to the *R*3*m*, *R*3*m*, *R*3*m, I-*43*m,* and *P-*43*m* space groups, respectively. Pawley refinement was then performed in the 2*θ* range of 5–50° on the unit-cell parameters, zero point, and background terms with *Pseudo*–*Voigt* profile function and *Berar*–*Baldinozzi* asymmetry correction function. Considering the cell originated from the reported structure α-ZIF-65(Zn)^57^, the initial structure model for the Rietveld refinement was constructed by rebuilding the crystal symmetry and redefining the lattice to obtain the corresponding space groups, by using the build module of Materials Studio 8.0^61^. The unit cell of each model is given based on the result of the Pawley refinement, and the number of guests is decided by the vapor absorption of each sample. Finally, each structure model is optimized by the Forcite module of Materials Studio 8.0^61^. The Rietveld refinement was then performed in the 2*θ* range of 5–80° on the unit-cell parameters, zero point, and background terms with *Pseudo*–*Voigt* profile function, *Berar*–*Baldinozzi* asymmetry correction function, and *Rietveld*-*Toraya* Preferred Orientation function. All atoms are treated with global anisotropic temperature factors. CCDC 2123793 [ZIF-65(Zn)-II], 2123794 [ZIF-65(Zn)-III·(*n*-C10)], 2123795 [ZIF-65(Zn)-III·(*n*-C4OH)], 2123796 [ZIF-65(Zn)-I·(*i*-C4OH)] and 2123797 [ZIF-65(Zn)-I] contain the supplementary crystallographic data for this paper. These data are obtained free of charge by The Cambridge Crystallographic Data Centre.

### Basic characterization

PXRD analysis was performed on an X-ray diffractometer (Rigaku, UItima IV) with Cu Kα radiation (*λ* = 1.5418 Å). Solid-state ^13^C nuclear magnetic resonance (^13^C NMR) spectroscopy was performed at 151 MHz (14.1 T) on Bruker Advance III 600 WB spectrometer using a 4 mm magic-angle spinning (MAS) probe with a spinning speed of 10 kHz. The cross-polarization (CP) MAS spectroscopy was recorded with 2 s recycle delays and 4 ms contact times; high power proton decoupling (HPDEC) MAS spectroscopy was recorded with 2 s recycle delays.

### Liquid, vapor, and gas adsorption

ZIF-65(Zn)-II samples were soaked in various polar/nonpolar and linear/branched solvents for 12 h at room temperature and then filtered, respectively. When there was no liquid on the filter paper, their PXRD was collected to observe the structural transitions. The organic vapor adsorption isotherms of ZIF-65(Zn) at 298 K were measured on an automated gravimetric sorption analyzer (Surface Measurement Systems, DVS Resolution). The organic vapor sorption was analyzed at a relative pressure *P*/*P*_0_ (*P*_0_ is the saturation vapor pressure) in the range of 0–90%. High-pressure CO_2_ and N_2_ adsorption isotherms of ZIF-65(Zn) were carried out using volumetric methods at 298 K (BSD Instrument, PH1-1139-A).

### In situ PXRD

In situ PXRD measurements were carried out on a Bruker D8 Advance with Cu Kα radiation (*λ* = 1.5406 Å) in a 2*θ* range of 5–20° at a scanning rate of 4° min^−1^, which was equipped with a vapor adsorption system comprised of a bubbler loaded with the organic solvent. For the adsorption step, N_2_ was passed through the bubbler at a flow rate of 50 mL min^−1^ at 298 K, which then brings the organic vapor into the in-situ cell; for the desorption step, N_2_ flowed directly into the in-situ cell at a flow rate of 50 mL min^−1^. In terms of heating desorption, the samples were heated at a ramping rate of 5 °C min^−1^ from 298 to 423 K.

### Computational details

Molecular simulations were performed with the sorption code in Materials Studio 8.0^61^. The GCMC method was applied to simulate the adsorption isotherms of the organic molecules in the empty ZIF-65(Zn) and the guest adsorption heat. The MC method was conducted by fixing the loading, which can not only be used to evaluate the guest adsorption heat, but also to determine the initial adsorption site. Alkanes and alcohols were denoted by the united-atom models with each CH_*x*_ acting as a single interaction site, in which the potential parameters were employed by the transferable potentials for the phase equilibria (TraPPE) force field^62–64^. All of the ZIF-65(Zn) frameworks, including ZIF-65(Zn)-II, ZIF-65(Zn)-III, ZIF-65(Zn)-I_*I*-43*m*, and ZIF-65(Zn)-I_*P*-43*m*, were kept rigid during the simulations. The Lennard–Jones 12-6 (LJ) potentials parameters of ZIF-65(Zn) were described by the DREIDING force field^65^. The partial atomic charges of ZIF-65(Zn)-I_*I*-43*m* were taken from the reported work of Nieto-Draghi et al.^66^. The atomic charges of other ZIF-65(Zn) structures were calculated according to the method of our previous work^67^. The fragmental clusters and charges are described in Supplementary Figs. 47–51 and Supplementary Tables 8–12. The vdW and electrostatic interactions were set using atom-based (cut-off radius of 12.8 Å) and Ewald sum methods, respectively. The adsorption simulations used 1.0 × 10^7^ steps to reach equilibration, followed by 1.0 × 10^7^ steps to collect the data.

The DFT calculations were conducted using the Dmol3 code in Materials Studio 8.0^61^ to assess the host energy difference between different ZIF-65(Zn) phases. The atomic positions and shape of the unit cell were allowed fully relaxed during the optimization. Due to the large unit cell of each ZIF-65(Zn) phase, only the Gamma point was sampled. We used the generalized gradient approximation with the Perdew–Burke–Ernzerhof functional, Tkatchenko–Scheffler method for density functional dispersion correction, the DFT Semi-core Pseudopots core treatment, and the double numerical plus functions basis set. The energy, force, and displacement convergence were set to be 1 × 10^–5^ Ha, 2 × 10^–3^ Ha, and 5 × 10^–3^ Å, respectively.

The self-consistent charge density-functional tight-binding method was employed using the DFTB + code^68^ to further explore the host–guest and guest–guest interactions. The configurations of the organic molecules in ZIF-65(Zn) obtained from the MC simulations were selected as the initial structures to be optimized by the DFTB calculations.

## Supplementary information

Supplementary Information

## Data Availability

The experimental data supporting this study are provided in this article and its Supplementary Information. The raw data are available from the corresponding author upon request. The X-ray crystallographic coordinates for structures reported in this study have been deposited at the Cambridge Crystallographic Data Centre (CCDC), under the deposition numbers: 2123793 [ZIF-65(Zn)-II], 2123794 [ZIF-65(Zn)-III·(*n*-C10)], 2123795 [ZIF-65(Zn)-III·(*n*-C4OH)], 2123796 [ZIF-65(Zn)-I·(*i*-C4OH)] and 2123797 [ZIF-65(Zn)-I]. These data can be obtained free of charge from The Cambridge Crystallographic Data Centre via www.ccdc.cam.ac.uk/data_request/cif.

## References

[1] Lee JH, Jeoung S, Chung YG, Moon HR (2019). Elucidation of flexible metal-organic frameworks: research progresses and recent developments. Coord. Chem. Rev..

[2] Schneemann A (2014). Flexible metal-organic frameworks. Chem. Soc. Rev..

[3] Férey G, Serre C (2009). Large breathing effects in three-dimensional porous hybrid matter: facts, analyses, rules and consequences. Chem. Soc. Rev..

[4] Horike S, Shimomura S, Kitagawa S (2009). Soft porous crystals. Nat. Chem..

[5] Krause S, Hosono N, Kitagawa S (2020). Chemistry of soft porous crystals: structural dynamics and gas adsorption properties. Angew. Chem. Int. Ed..

[6] Kim JY (2020). Specific isotope-responsive breathing transition in flexible metal-organic frameworks. J. Am. Chem. Soc..

[7] Yang M (2021). Fabrication of moisture-responsive crystalline smart materials for water harvesting and electricity transduction. J. Am. Chem. Soc..

[8] Kundu T, Wahiduzzaman M, Shah BB, Maurin G, Zhao D (2019). Solvent-induced control over breathing behavior in flexible metal-organic frameworks for natural-gas delivery. Angew. Chem. Int. Ed..

[9] Chen Q (2021). High-efficiency separation of *n*-hexane by a dynamic metal-organic framework with reduced energy consumption. Angew. Chem. Int. Ed..

[10] Hiraide S (2020). High-throughput gas separation by flexible metal-organic frameworks with fast gating and thermal management capabilities. Nat. Commun..

[11] Chanut N (2020). Tailoring the separation properties of flexible metal-organic frameworks using mechanical pressure. Nat. Commun..

[12] Pallach R (2021). Frustrated flexibility in metal-organic frameworks. Nat. Commun..

[13] Wang J (2022). Fine pore engineering in a series of isoreticular metal-organic frameworks for efficient C_2_H_2_/CO_2_ separation. Nat. Commun..

[14] Park KS (2006). Exceptional chemical and thermal stability of zeolitic imidazolate frameworks. Proc. Natl Acad. Sci. USA.

[15] Banerjee R (2008). High-throughput synthesis of zeolitic imidazolate frameworks and application to CO_2_ capture. Science.

[16] Phan A (2010). Synthesis, structure, and carbon dioxide capture properties of zeolitic imidazolate frameworks. Acc. Chem. Res..

[17] Shi Q (2016). Zeolite CAN and AFI-type zeolitic imidazolate frameworks with large 12-membered ring pore openings synthesized using bulky amides as structure-directing agents. J. Am. Chem. Soc..

[18] Eddaoudi M, Sava DF, Eubank JF, Adil K, Guillerm V (2015). Zeolite-like metal-organic frameworks (ZMOFs): design, synthesis, and properties. Chem. Soc. Rev..

[19] Huang X-C, Lin Y-Y, Zhang J-P, Chen X-M (2006). Ligand-directed strategy for zeolite-type metal-organic frameworks: zinc(II) imidazolates with unusual zeolitic topologies. Angew. Chem. Int. Ed..

[20] Zhang J-P, Zhang Y-B, Lin J-B, Chen X-M (2012). Metal azolate frameworks: from crystal engineering to functional materials. Chem. Rev..

[21] Wu T (2008). A new zeolitic topology with sixteen-membered ring and multidimensional large pore channels. Chem. Eur. J..

[22] Wu T, Bu X, Zhang J, Feng P (2008). New zeolitic imidazolate frameworks: from unprecedented assembly of cubic clusters to ordered cooperative organization of complementary ligands. Chem. Mater..

[23] Zhang J (2009). Zeolitic boron imidazolate frameworks. Angew. Chem. Int. Ed..

[24] Zhang H-X, Liu M, Wen T, Zhang J (2016). Synthetic design of functional boron imidazolate frameworks. Coord. Chem. Rev..

[25] Moggach SA, Bennett TD, Cheetham AK (2009). The effect of pressure on ZIF-8: increasing pore size with pressure and the formation of a high-pressure phase at 1.47 GPa. Angew. Chem. Int. Ed..

[26] Fairen-Jimenez D (2011). Opening the gate: framework flexibility in ZIF-8 explored by experiments and simulations. J. Am. Chem. Soc..

[27] Hobday CL (2018). Tuning the swing effect by chemical functionalization of zeolitic imidazolate frameworks. J. Am. Chem. Soc..

[28] Chaplais G (2018). Impacts of the imidazolate linker substitution (CH_3_, Cl, or Br) on the structural and adsorptive properties of ZIF-8. J. Phys. Chem. C..

[29] Knebel A (2017). Defibrillation of soft porous metal-organic frameworks with electric fields. Science.

[30] Ryder MR (2014). Identifying the role of terahertz vibrations in metal-organic frameworks: from gate-opening phenomenon to shear-driven structural destabilization. Phys. Rev. Lett..

[31] Wee LH (2021). Chlorination of a zeolitic-imidazolate framework tunes packing and van der Waals interaction of carbon dioxide for optimized adsorptive separation. J. Am. Chem. Soc..

[32] Ni, Z., Afeworki, M., Weston, S. C., Zengel, J. & Stern, D. L. Linker exchange in zeolitic imidazolate frameworks. US2013259783(A1).

[33] Falkowski JM (2022). Tunable hydrocarbon adsorption based on a zeolitic imidazolate framework in the sodalite topology. J. Mater. Chem. A.

[34] Gücüyener C, van den Bergh J, Gascon J, Kapteijn F (2010). Ethane/ethene separation turned on its head: selective ethane adsorption on the metal-organic framework ZIF-7 through a gate-opening mechanism. J. Am. Chem. Soc..

[35] van den Bergh J (2011). Understanding the anomalous alkane selectivity of ZIF-7 in the separation of light alkane/alkene mixtures. Chem. Eur. J..

[36] Aguado S (2011). Guest-induced gate-opening of a zeolite imidazolate framework. N. J. Chem..

[37] Cai W (2014). Thermal structural transitions and carbon dioxide adsorption properties of zeolitic imidazolate framework-7 (ZIF-7). J. Am. Chem. Soc..

[38] Zhao P, Lampronti GI, Lloyd GO, Suard E, Redfern SAT (2014). Direct visualisation of carbon dioxide adsorption in gate-opening zeolitic imidazolate framework ZIF-7. J. Mater. Chem. A.

[39] Zhao P (2014). Phase transitions in zeolitic imidazolate framework 7: the importance of framework flexibility and guest-induced instability. Chem. Mater..

[40] Zhao P (2015). Pressure-induced oversaturation and phase transition in zeolitic imidazolate frameworks with remarkable mechanical stability. Dalton Trans..

[41] Zhao P (2019). Structural dynamics of a metal-organic framework induced by CO_2_ migration in its non-uniform porous structure. Nat. Commun..

[42] Du Y (2015). New high- and low-temperature phase changes of ZIF-7: elucidation and prediction of the thermodynamics of transitions. J. Am. Chem. Soc..

[43] Du Y (2017). Insights into the flexibility of ZIF-7 and its structural impact in alcohol adsorption. J. Phys. Chem. C..

[44] Klein RA (2021). Structural resolution and mechanistic insight into hydrogen adsorption in flexible ZIF-7. Chem. Sci..

[45] Jo YK (2021). Exclusive and ultrasensitive detection of formaldehyde at room temperature using a flexible and monolithic chemiresistive sensor. Nat. Commun..

[46] McGuirk CM (2018). Influence of metal substitution on the pressure-induced phase change in flexible zeolitic imidazolate frameworks. J. Am. Chem. Soc..

[47] Bennett TD, Cheetham AK, Fuchs AH, Coudert F-X (2017). Interplay between defects, disorder and flexibility in metal-organic frameworks. Nat. Chem..

[48] Bennett TD, Horike S (2018). Liquid, glass and amorphous solid states of coordination polymers and metal–organic frameworks. Nat. Rev. Mater..

[49] Noh K, Lee J, Kim J (2018). Compositions and structures of zeolitic imidazolate frameworks. Israel J. Chem..

[50] Biswal BP, Pandaa T, Banerjee R (2012). Solution mediated phase transformation (RHO to SOD) in porous Co-imidazolate based zeolitic frameworks with high water stability. Chem. Commun..

[51] Diring S (2013). Localized cell stimulation by nitric oxide using a photoactive porous coordination polymer platform. Nat. Commun..

[52] Ban Y (2014). Metal-substituted zeolitic imidazolate framework ZIF-108: gas-sorption and membrane-separation properties. Chem. Eur. J..

[53] Tu M, Wiktor C, Röslera C, Fischer RA (2014). Rapid room temperature syntheses of zeolitic-imidazolate framework (ZIF) nanocrystals. Chem. Commun..

[54] Tu M, Wannapaiboon S, Khaletskaya K, Fischer RA (2015). Engineering zeolitic-imidazolate framework (ZIF) thin film devices for selective detection of volatile organic compounds. Adv. Funct. Mater..

[55] Orsi A (2017). Porous zinc and cobalt 2-nitroimidazolate frameworks with six-membered ring windows and a layered cobalt 2-nitroimidazolate polymorph. CrystEngComm.

[56] Bhattacharyya S (2018). Acid gas stability of zeolitic imidazolate frameworks: generalized kinetic and thermodynamic characteristics. Chem. Mater..

[57] Choi Y, Noh K, Lee J, Kim J (2018). Porosity properties of the conformers of sodalite-like zeolitic imidazolate frameworks. J. Am. Chem. Soc..

[58] Serre C (2007). Role of solvent-host interactions that lead to very large swelling of hybrid frameworks. Science.

[59] Coudert F-X, Jeffroy M, Fuchs AH, Boutin A, Mellot-Draznieks C (2008). Thermodynamics of guest-induced structural transitions in hybrid organic-inorganic frameworks. J. Am. Chem. Soc..

[60] Llewellyn PL (2008). Prediction of the conditions for breathing of metal organic framework materials using a combination of X-ray powder diffraction, microcalorimetry, and molecular simulation. J. Am. Chem. Soc..

[61] Dassault Systèmes BIOVIA. *Materials Studio Modeling Environment*, *Release 2017*. (Dassault Systèmes BIOVIA, San Diego, CA, 2016).

[62] Martin MG, Siepmann JI (1998). Transferable potentials for phase equilibria. 1. united-atom description of *n*-alkanes. J. Phys. Chem. B.

[63] Chen B, Potoff JJ, Siepmann JI (2001). Monte carlo calculations for alcohols and their mixtures with alkanes. transferable potentials for phase equilibria. 5. united-atom description of primary, secondary, and tertiary alcohols. J. Phys. Chem. B.

[64] Keasler SJ, Charan SM, Wick CD, Economou IG, Siepmann JI (2012). Transferable potentials for phase equilibria-united atom description of five- and six-membered cyclic alkanes and ethers. J. Phys. Chem. B.

[65] Mayo SL, Olafson BD, Goddard WA (1990). Dreiding: a generic force field for molecular simulations. J. Phys. Chem..

[66] Amrouche H (2011). Experimental and computational study of functionality impact on sodalite–zeolitic imidazolate frameworks for CO_2_ separation. J. Phys. Chem. C..

[67] Gao M, Wang J, Rong Z, Shi Q, Dong J (2018). A combined experimental-computational investigation on water adsorption in various ZIFs with the SOD and RHO topologies. RSC Adv..

[68] Official internet link to the program DFTB+: http://www.dftb-plus.info/.

