# Supplementary material for: Large breathing effect in ZIF-65(Zn) with expansion and contraction of the SOD cage
Source: Nat Commun. 2022 Aug 5;13:4569. doi: 10.1038/s41467-022-32332-x (PMC9355966; doi:10.1038/s41467-022-32332-x)
Supplement: Supplementary file 1 — Supplementary Information [file 41467_2022_32332_MOESM1_ESM.pdf]

Supplementary Information

Large breathing effect in ZIF-65(Zn) with expansion and contraction of the SOD cage

Meizhen Gao[1,#], Rui-Kang Huang[2,#], Bin Zheng[3,#], Pengfei Wang[4], Qi Shi[1,*], Wei-Xiong Zhang[2] & Jinxiang Dong[1]

[1] College of Chemistry and Chemical Engineering, Taiyuan University of Technology, Taiyuan 030024, Shanxi, China.

[2] MOE Key Laboratory of Bioinorganic and Synthetic Chemistry, School of Chemistry, Sun Yat-Sen University, Guangzhou 510275, Guangdong, China.

[3] School of Materials Science and Engineering, Xi′an University of Science and Technology, Xi′an 710054, Shaanxi, China.

[4] State Key Laboratory of Coal Conversion, Institute of Coal Chemistry, Chinese Academy of Sciences, Taiyuan 030001, Shanxi, China.

[#] These authors contributed equally: Meizhen Gao, Rui-Kang Huang, and Bin Zheng.

[*] Corresponding author: Qi Shi. Email: shiqi594@163.com

Table of Contents

Supplementary Methods
S1.1 Synthesis and activation of ZIF-65(Zn)
S1.2 Preparation of different ZIF-65(Zn) phases
S1.3 Structural model and Rietveld refinement of ZIF-65(Zn)
S1.4 Basic characterization of ZIF-65(Zn)
S1.5 Liquid adsorption, vapor adsorption, and high-pressure gas adsorption of ZIF-65(Zn)
S1.6 In situ PXRD for vapor adsorption and desorption of ZIF-65(Zn)
S1.7 Computational details
Supplementary Discussion
S2.1 Activation strategies for ZIF-65(Zn)
Supplementary Fig. 1
Supplementary Fig. 2
Supplementary Fig. 3
Supplementary Table 1
Supplementary Fig. 4
Supplementary Fig. 5
Supplementary Fig. 6
S2.2 The structural analysis of ZIF-65(Zn)
Supplementary Fig. 7
Supplementary Fig. 8
Supplementary Fig. 9
Supplementary Fig. 10
Supplementary Fig. 11
Supplementary Table 2
Supplementary Fig. 12
Supplementary Table 3
Supplementary Fig. 13
Supplementary Table 4
Supplementary Fig. 14
Supplementary Table 5
Supplementary Table 6
Supplementary Fig. 15
S2.3 The liquid adsorption of ZIF-65(Zn)
Supplementary Table 7
Supplementary Fig. 16
Supplementary Fig. 17
S2.4 The vapor adsorption of ZIF-65(Zn)
Supplementary Fig. 18
Supplementary Fig. 19
Supplementary Fig. 20
Supplementary Fig. 21
Supplementary Fig. 22
Supplementary Fig. 23
Supplementary Fig. 24
Supplementary Fig. 25
Supplementary Fig. 26
Supplementary Fig. 27
Supplementary Fig. 28
Supplementary Fig. 29
Supplementary Fig. 30
Supplementary Fig. 31
Supplementary Fig. 32
Supplementary Fig. 33

Supplementary Fig. 34
Supplementary Fig. 35
Supplementary Fig. 36
Supplementary Fig. 37
Supplementary Fig. 38
Supplementary Fig. 39
Supplementary Fig. 40
S2.5 The dynamic structural transition of ZIF-65(Zn)
Supplementary Fig. 41
Supplementary Fig. 42
Supplementary Fig. 43
Supplementary Fig. 44
Supplementary Fig. 45
Supplementary Fig. 46
S2.6 Understanding the flexibility of ZIF-65(Zn)
Supplementary Fig. 47
Supplementary Table 8
Supplementary Fig. 48
Supplementary Table 9
Supplementary Fig. 49
Supplementary Table 10
Supplementary Fig. 50
Supplementary Table 11
Supplementary Fig. 51
Supplementary Table 12
Supplementary Fig. 52
Supplementary Fig. 53
Supplementary Fig. 54
Supplementary Fig. 55
Supplementary Fig. 56
Supplementary Table 13
Supplementary Table 14
Supplementary Fig. 57
Supplementary Fig. 58
Supplementary Table 15
Supplementary Fig. 59
Supplementary Table 16
Supplementary Fig. 60
Supplementary Fig. 61
Supplementary Fig. 62
Supplementary Fig. 63
Supplementary Fig. 64
Supplementary Fig. 65
Supplementary Fig. 66
Supplementary References

Supplementary Methods

S1.1 Synthesis and activation of ZIF-65(Zn)

ZIF-65(Zn) was synthesized using a modification of the method published by Yaghi *et al*[1]. Zinc acetate dehydrate [0.110 g, 0.5 mmol] and 2-nitroimidazole (nIm) [0.141 g, 1.25 mmol] were added to dimethylformamide (DMF) [15 mL] in a 30 mL Teflon-lined autoclave and heated at 100 °C for 48 h. The as-synthesized sample was labeled as ZIF-65(Zn)-I.

Methanol exchange and activation: the as-synthesized sample was immersed in methanol (MeOH) for 24 h, changing the solvent with fresh MeOH at 12 h intervals. The exchanged sample was further activated by heating at 50 °C for 12 h or vacuuming at 150 °C for 12 h, which was labeled as ZIF-65(Zn)-II.

Ethanol exchange and activation: the as-synthesized sample was immersed in ethanol (EtOH) for 36 h, changing the solvent with fresh EtOH at 12 h intervals. The exchanged sample was further activated by vacuuming at 50 °C for 1 h, which was also labeled as ZIF-65(Zn)-I.

S1.2 Preparation of different ZIF-65(Zn) phases

ZIF-65(Zn)-II: ZIF-65(Zn)-II was obtained via MeOH exchange and activation of the as-synthesized sample. ZIF-65(Zn)-II can be obtained by desorption of specific guest molecules in ZIF-65(Zn)-III or ZIF-65(Zn)-I.

ZIF-65(Zn)-III: ZIF-65(Zn)-III can be obtained by adsorbing specific guest molecules in ZIF-65(Zn)-II. Typically, ZIF-65(Zn)-III·(*n*-C10) was formed using *n*-decane (*n*-C10) vapor adsorption in ZIF-65(Zn)-II or immersing the ZIF-65(Zn)-II sample in *n*-C10. ZIF-65(Zn)-III·(*n*-C4OH) was formed via *n*-butanol (*n*-C4OH) vapor adsorption in ZIF-65(Zn)-II.

ZIF-65(Zn)-I: ZIF-65(Zn)-I (Cubic *I*-43*m*) was synthesized using DMF as the reaction solvent. ZIF-65(Zn)-I (Cubic *I*-43*m*) can also be obtained by adsorbing specific guest molecules in ZIF-65(Zn)-II. Typically, ZIF-65(Zn)-I·(*i*-C4OH) was obtained by immersing the ZIF-65(Zn)-II sample in isobutanol (*i*-C4OH). ZIF-65(Zn)-I (Cubic *P*-43*m*) was obtained via EtOH exchange and activation of the as-synthesized sample.

S1.3 Structural model and Rietveld refinement of ZIF-65(Zn)

The powder X-ray diffraction (PXRD) data obtained for structural refinement was collected on a Bruker D8 Advance diffractometer equipped with Cu Kα radiation (λ = 1.5418 Å) at 40 kV and 40 mA, in the 2θ range of 5–80° with a scan step size of 0.02° and 4 s per step. The indexing and refinement of the PXRD patterns were carried out using the Reflex module of Materials Studio 8.0[2]. The patterns of ZIF-65(Zn)-II, ZIF-65(Zn)-III·(*n*-C10), ZIF-65(Zn)-III·(*n*-C4OH), ZIF-65(Zn)-I·(*i*-C4OH) and ZIF-65(Zn)-I were well indexed to the *R*3*m*, *R*3*m*, *R*3*m*, *I*-43*m* and *P*-43*m* space groups, respectively. Pawley refinement was then performed in the 2θ range of 5–50° on the unit-cell parameters, zero point, and background terms with *Pseudo–Voigt* profile function and *Berar–Baldinozzi* asymmetry correction function. Considering the cell originated from the reported structure α-ZIF-65(Zn)[3], the initial structure model for the Rietveld refinement was constructed by rebuilding the crystal symmetry and redefining the lattice to obtain the corresponding space groups, by using the build module of Materials Studio 8.0[2]. The unit cell of each model is given based on the result of the Pawley refinement, and the number of guests is decided by the vapor absorption of each sample. Finally, each structure model is optimized by the Forcite module of Materials Studio 8.0[2]. The Rietveld refinement was then performed in the 2θ range of 5–80° on the unit-cell parameters, zero point, and background terms with *Pseudo–Voigt* profile function, *Berar–Baldinozzi* asymmetry correction function, and *Rietveld-Toraya* Preferred Orientation function. All atoms are treated with global anisotropic temperature factors. CCDC 2123793 [ZIF-65(Zn)-II], 2123794 [ZIF-65(Zn)-III·(*n*-C10)], 2123795 [ZIF-65(Zn)-III·(*n*-C4OH)], 2123796 [ZIF-65(Zn)-I·(*i*-C4OH)] and 2123797 [ZIF-65(Zn)-I] contain the supplementary crystallographic data for this paper. These data are obtained free of charge by The Cambridge Crystallographic Data Centre.

S1.4 Basic characterization of ZIF-65(Zn)

Powder X-ray diffraction (PXRD) analysis was performed on an X-ray diffractometer (Rigaku, Ultima IV) with Cu Kα radiation (λ = 1.5418 Å). Thermogravimetric (TG) analysis was conducted on a simultaneous thermal analyzer (Setaram, Labsys Evo) under an air atmosphere. The mass loss as a function of time at 298 K was measured on an automated gravimetric sorption analyzer (Surface Measurement Systems, DVS Resolution). N_2 adsorption and desorption at 77 K were measured using an automated volumetric adsorption apparatus (Micromeritics, ASAP2010). Scanning electron microscopy (SEM) images were obtained on a scanning electron microscope (Hitachi, SU8010). Solid-state ^{13}C nuclear magnetic resonance (^{13}C NMR) spectroscopy was performed at 151 MHz (14.1 T) on Bruker Advance III 600 WB spectrometer using a 4 mm magic-angle spinning (MAS) probe with a spinning speed of 10 kHz. The cross-polarization (CP) MAS spectroscopy was recorded with 2 s recycle delays and 4 ms contact times; high power proton decoupling (HPDEC) MAS spectroscopy was recorded with 2 s recycle delays.

S1.5 Liquid adsorption, vapor adsorption, and high-pressure gas adsorption of ZIF-65(Zn)

ZIF-65(Zn)-II (no guest) samples were soaked in various polar/nonpolar and linear/branched solvents for 12 h at room temperature and then filtered, respectively. When there was no liquid on the filter paper, their PXRD was collected to observe the structural transitions. The organic vapor adsorption isotherms of ZIF-65(Zn)-I (no guest) and ZIF-65(Zn)-II (no guest) at 298 K were measured on an automated gravimetric sorption analyzer (Surface Measurement Systems, DVS Resolution). The organic vapor sorption was analyzed at a relative pressure P/P_0 (P_0 is the saturation vapor pressure) in the range of 0–90%. The vapor pressure was controlled automatically by mixing the wet vapor feed with a dry N_2 line. High-pressure CO_2 and N_2 adsorption isotherms of ZIF-65(Zn)-I (no guest) and ZIF-65(Zn)-II (no guest) were carried out using volumetric methods at 298 K (BSD Instrument, PH1-1139-A).

S1.6 In situ PXRD for vapor adsorption and desorption of ZIF-65(Zn)

In situ PXRD measurements were carried out on a Bruker D8 Advance with Cu Kα radiation (λ = 1.5406 Å) in a 2θ range of 5–20° at a scanning rate of 4° min^{-1}, which was equipped with a vapor adsorption system comprised of a bubbler loaded with the organic solvent. For the adsorption step, N_2 was passed through the bubbler at a flow rate of 50 mL min^{-1} at 298 K, which then brings the organic vapor into the in-situ cell; for the desorption step, N_2 flowed directly into the in-situ cell at a flow rate of 50 mL min^{-1}. In terms of heating desorption, the samples were heated at a ramping rate of 5 °C min^{-1} from 298 to 423 K.

S1.7 Computational details

Molecular simulations were performed with the sorption code in Materials Studio 8.0[2]. The grand canonical Monte Carlo (GCMC) method was applied to simulate the adsorption isotherms of the organic molecules in the empty ZIF-65(Zn) and the guest adsorption heat under the corresponding adsorption capacity. The Monte Carlo (MC) method was conducted by fixing the loading, which can not only be used to evaluate the guest adsorption heat, but also to determine the initial adsorption site. Alkanes and alcohols were denoted by the united-atom models with each CH_x acting as a single interaction site, in which the potential parameters were employed by the transferable potentials for the phase equilibria (TraPPE) force field[4-6]. All of the ZIF-65(Zn) frameworks, including ZIF-65(Zn)-II, ZIF-65(Zn)-III, ZIF-65(Zn)-I_*I*-43*m*, and ZIF-65(Zn)-I_*P*-43*m*, were kept rigid during the simulations. The Lennard-Jones 12-6 (LJ) potentials parameters of ZIF-65(Zn) were described by the DREIDING force field[7]. The partial atomic charges of ZIF-65(Zn)-I_*I*-43*m* were taken from the reported work of Nieto-Draghi *et al*[8]. The atomic charges of other ZIF-65(Zn) structures were calculated according to the method of our previous work[9]. The fragmental clusters and charges are described in Supplementary Fig. 47-51 and Supplementary Table 8-12. The vdW and electrostatic interactions were set using atom-based (cut-off radius of 12.8 Å) and Ewald sum methods, respectively. The adsorption simulations used 1.0×10^7 steps to reach equilibration, followed by 1.0×10^7 steps to collect the data.

The density functional theory (DFT) calculations were conducted using the Dmol3 code in Materials Studio 8.0[2] to assess the host energy difference between different ZIF-65(Zn) phases. The atomic positions and shape of the unit cell were allowed fully relaxed during the optimization. Due to the large unit cell of each ZIF-65(Zn) phase, only the Gamma point was sampled. We used the generalized gradient approximation (GGA) with the Perdew-Burke-Ernzerhof (PBE) functional, Tkatchenko-Scheffler (TS) method for density functional dispersion (DFT-D)

correction, the DFT Semi-core Pseudopots (DSPP) core treatment, and the double numerical plus functions (DNP) basis set. The energy, force, and displacement convergence were set to be 1×10^{-5} Ha, 2×10^{-3} Ha, and 5×10^{-3} Å, respectively.

The self-consistent charge density-functional tight-binding (SCC-DFTB)[10,11] method is augmented by the empirical London dispersion energy term using the DFTB+ code[12] to further explore the host–guest and guest–guest interactions. The configurations of the organic molecules in ZIF-65(Zn) obtained from the MC simulations were selected as the initial structures to be optimized by the DFTB calculations. Zn 3d-orbitals, O 2p-orbitals, C 2p-orbitals, N 2p-orbitals, and H 1s-orbitals have been considered for the tight-binding basis sets. The force and self-consistent charge conversion thresholds for relaxing the atomic coordinates and the self-consistent cycles were 1E-4 H/Bohr and 1E-6 H, respectively. The set of DFTB parameterizations for the Zinc-organic systems, the third-order parametrization for organic and biological systems ("3ob") parameter set[13], was employed in this study. The 3ob parameter set was found to give reliable structure properties of Zn contained organic structures, such as ZIF-8[14]. Additionally, the non-bonding van der Waals interactions considering the Beck-Johnson damping[15,16] were chosen.

Supplementary Discussion

S2.1 Activation strategies for ZIF-65(Zn)

Although it is generally believed that the structure of ZIF-65(Zn)-I (as-synthesized) turned into an unknown structure [labeled as ZIF-65(Zn)-II] after the removal of guest molecules[3,17-19], we herein report an effective activation method to remove the guest molecules of ZIF-65(Zn)-I which still maintain its structural integrity.

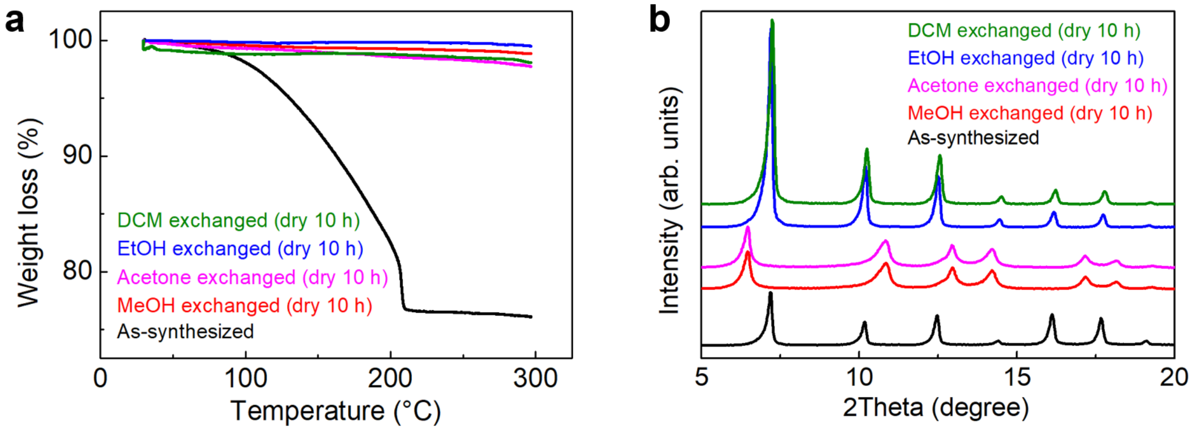

Supplementary Fig. 1 Activation of ZIF-65(Zn)-I by MeOH/acetone/EtOH/DCM. a TG curves and **b** PXRD patterns for ZIF-65(Zn)-I by solvent exchange and further drying. [Note: dichloromethane (DCM)]

ZIF-65(Zn)-I was prepared using DMF as the reaction solvent, which ultimately fills the pore space. Thus, the samples were firstly immersed in various lower boiling point solvents to remove the DMF present in the framework. Treatment with MeOH/acetone/EtOH/DCM simplified the thermogravimetric behavior of ZIF-65(Zn)-I significantly and the TG curves only show a tiny weight loss step of 2.5 wt.% in the temperature range 25–300 °C (Supplementary Fig. 1a), which indicate ZIF-65(Zn)-I can be effectively solvent-exchanged. However, our PXRD results (Supplementary Fig. 1b) show that MeOH/acetone-exchanged ZIF-65(Zn)-I structure was not thermally stable, and transformed into another crystalline material ZIF-65(Zn)-II, which has been often observed in literature[19], whereas EtOH/DCM-exchanged ZIF-65(Zn)-I maintained its structure. The above observations motivated us to study this solvent-exchange process in detail, and MeOH and EtOH were selected as the exchange solvents for comparison (Supplementary Fig. 2).

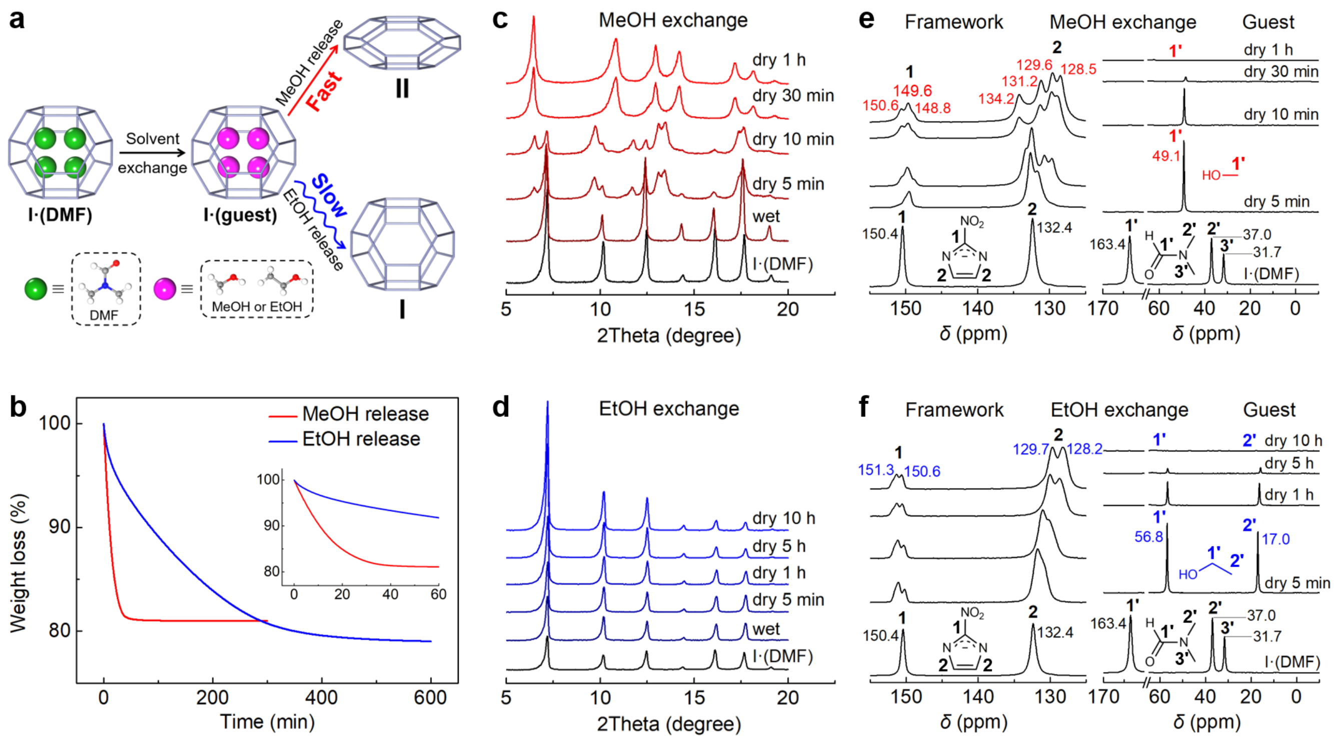

Supplementary Fig. 2 Activation mechanism of ZIF-65(Zn). a Schematic representation of the activation of ZIF-65(Zn)-I. **b** The weight loss as a function of time at 298 K for MeOH- and EtOH-exchanged ZIF-65(Zn)-I. PXRD monitoring at 298 K with increasing time for **c** MeOH- and **d** EtOH-exchanged ZIF-65(Zn)-I. ^{13}C NMR spectra monitoring at 298 K with increasing time for **e** MeOH- and **f** EtOH-exchanged ZIF-65(Zn)-I. [The as-synthesized ZIF-65(Zn) using DMF was abbreviated as I·(DMF).]

A significant structural transition occurred when the MeOH-exchanged ZIF-65(Zn)-I sample was treated in the air at 298 K (Supplementary Fig. 2c). After 10 min, part of the ZIF-65(Zn)-I transformed into the ZIF-65(Zn)-II structure with the split and shifted of their diffraction peaks (such as 6.5° and 9.7°) and the corresponding ZIF-65(Zn) sample exhibited a weight loss of 9.6 wt.% corresponding to the fast release of nearly half of the MeOH molecules (Supplementary Fig. 2c and Supplementary Table 1). After 30 min, all original characteristic peaks of ZIF-65(Zn)-I disappeared resulting from the loss of almost all of the MeOH molecules (17.7 wt.%). In contrast, the EtOH-exchanged ZIF-65(Zn)-I maintained its structure when the sample was treated in the air at 298 K (Supplementary Fig. 2d). The release time of half and all of the EtOH molecules observed for EtOH-exchanged ZIF-65(Zn)-I sample was 1 h and 5 h, respectively (Supplementary Fig. 2b and Supplementary Table 1), which indicates that the EtOH molecules leave the **SOD** cage slowly when compared to MeOH. Therefore, the rapid release of guest molecules in the ZIF-65(Zn)-I structure leads to the occurrence of the structural transition.

Subsequently, the ^{13}C NMR spectrum was used as a local probe to investigate the fine framework change observed with the release of the guest molecules (Supplementary Fig. 2e, 2f, and Supplementary Fig. 3). Firstly, the characteristic carbon atoms peaks of DMF (δ = 163.4, 37.0, and 31.7 ppm) disappeared and the peaks of MeOH (δ = 49.1 ppm) and EtOH (δ = 56.8 and 17.0 ppm) were observed in the ^{13}C NMR spectra of the MeOH- and EtOH-exchanged ZIF-65(Zn)-I samples, which indicate the effective solvent-exchange. In addition, for the MeOH- and EtOH-exchanged ZIF-65(Zn)-I sample dried for 1 h, the characteristic carbon atoms peak of the MeOH was not observed and that of EtOH reduced by around a half in the ^{13}C NMR spectra, which further confirmed that the EtOH molecules are expected to leave the **SOD** cage slowly when compared to MeOH.

For the as-synthesized ZIF-65(Zn)-I, which contains one unique site for the nIm linker, the resonances observed at δ = 150.4 and 132.4 ppm correspond to C#1 and C#2 of nIm in ZIF-65(Zn)-I framework. For the MeOH-exchanged ZIF-65(Zn)-I sample (Supplementary Fig. 2e), carbon peaks of the nIm linker are gradually divided into broad and multiple with the gradual release of the MeOH molecules. Specifically, the ^{13}C NMR spectrum of the MeOH-exchanged ZIF-65(Zn)-I sample dried at 1 h shows three peaks (δ = 150.6/149.6/148.8 ppm) corresponding to C#1 of the nIm linker and four peaks (δ= 134.2/131.2/129.6/128.5 ppm) corresponding to C#2 of nIm linker, which has multiple conformations of nIm. Thus, the phase change in ZIF-65(Zn) associated with the fast release of

the MeOH molecules is also confirmed using ^{13}C NMR spectroscopy. Fortunately, Rietveld refinement was successfully performed for the phase change structure ZIF-65(Zn)-II. ZIF-65(Zn)-II exhibits a trigonal structure with the *R*3*m* space group and three unique sites for the nIm linker (Supplementary Fig. 13 and Supplementary Table 4), which are consistent with the ^{13}C NMR results.

For the EtOH-exchanged ZIF-65(Zn)-I sample dried at 10 h (Supplementary Fig. 2f), upon the full release of the EtOH molecules, C#1 and C#2 of the nIm linkers show two sharp peaks (δ = 151.3/150.6 ppm and 129.7/128.2 ppm) with a 1:1 ratio associated with the two nIm conformations, which are consistent with the Rietveld refinement structure ZIF-65(Zn)-I (Cubic *P*-43*m*) containing one unique Zn site and two unique sites for the nIm linker (Supplementary Fig. 13 and Supplementary Table 4). It should be noted that the PXRD pattern and unit cell parameters of the EtOH-exchanged ZIF-65(Zn)-I did not change significantly when compared to the original DMF-synthesized ZIF-65(Zn)-I (Cubic *I*-43*m*). Thus, EtOH is an effective exchange solvent for the activation of ZIF-65(Zn)-I without structural transition and the slow solvent release strategy can effectively activate the ZIF-65(Zn)-I sample (Supplementary Fig. 2a).

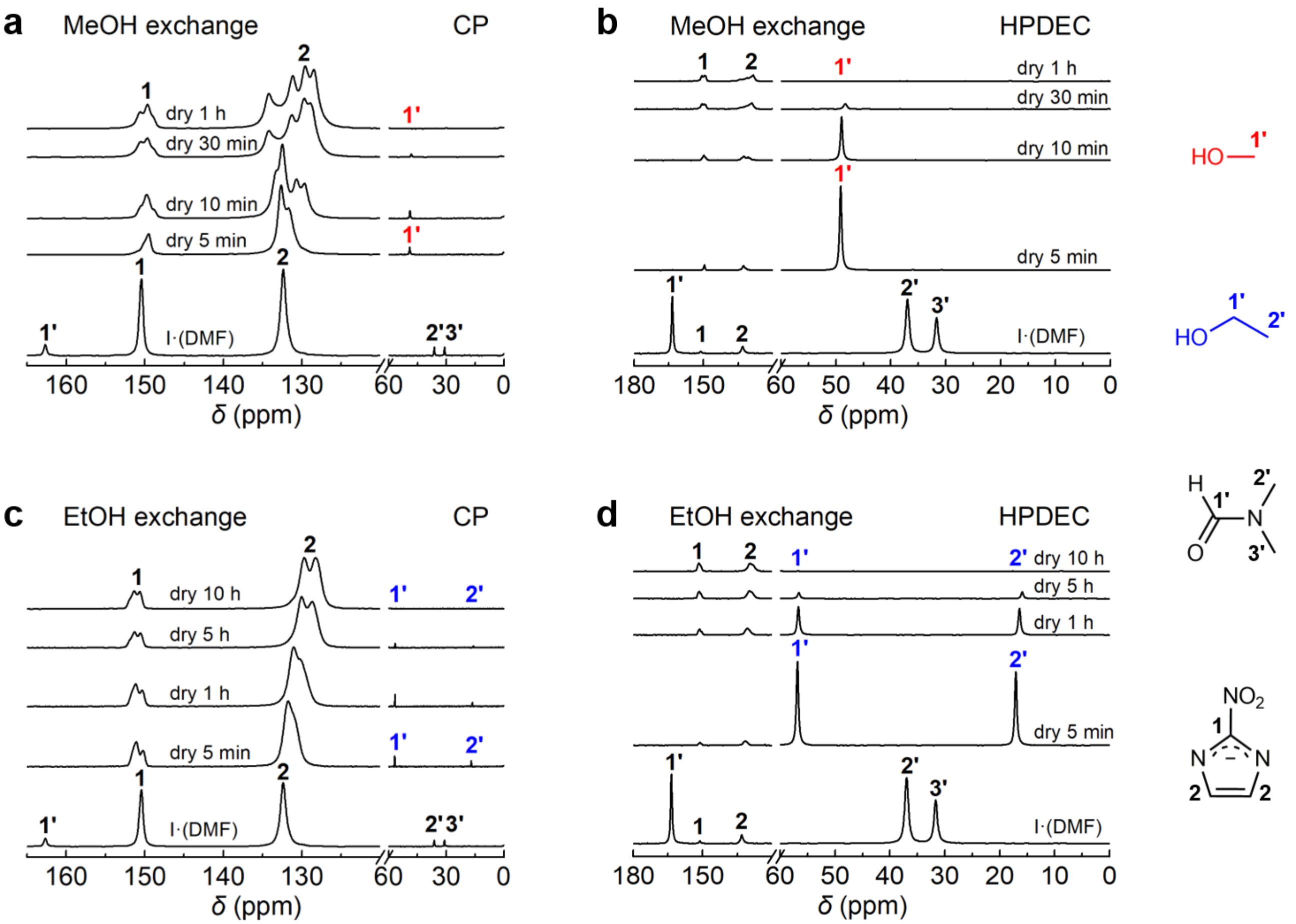

Supplementary Fig. 3 ^{13}C NMR spectra for activation of ZIF-65(Zn). a ^{13}C (CP) NMR and **b** ^{13}C (HPDEC) NMR spectra monitoring at 298 K with increasing time for MeOH-exchanged ZIF-65(Zn)-I. **c** ^{13}C (CP) NMR and **d** ^{13}C (HPDEC) NMR spectra monitoring at 298 K with increasing time for EtOH-exchanged ZIF-65(Zn)-I. [The as-synthesized ZIF-65(Zn) using DMF was abbreviated as I·(DMF).]

Supplementary Table 1 The detailed weight loss at 298 K for MeOH- and EtOH-exchanged ZIF-65(Zn)-I.

**MeOH release**		**EtOH release**			
**Time**	**Weight loss**	**Time**	**Weight loss**	**Time**	**Weight loss**
5 min	5.3 wt.%	5 min	2.0 wt.%	3 h	15.2 wt.%
10 min	9.6 wt.%	10 min	3.2 wt.%	4 h	17.6 wt.%
20 min	15.1 wt.%	20 min	4.7 wt.%	5 h	19.3 wt.%
30 min	17.7 wt.%	30 min	5.7 wt.%	6 h	20.1 wt.%
40 min	18.6 wt.%	40 min	6.6 wt.%	7 h	20.5 wt.%
50 min	18.8 wt.%	50 min	7.4 wt.%	8 h	20.7 wt.%
1 h	18.9 wt.%	1 h	8.2 wt.%	9 h	20.9 wt.%
5 h	19.0 wt.%	2 h	12.1 wt.%	10 h	21.0 wt.%

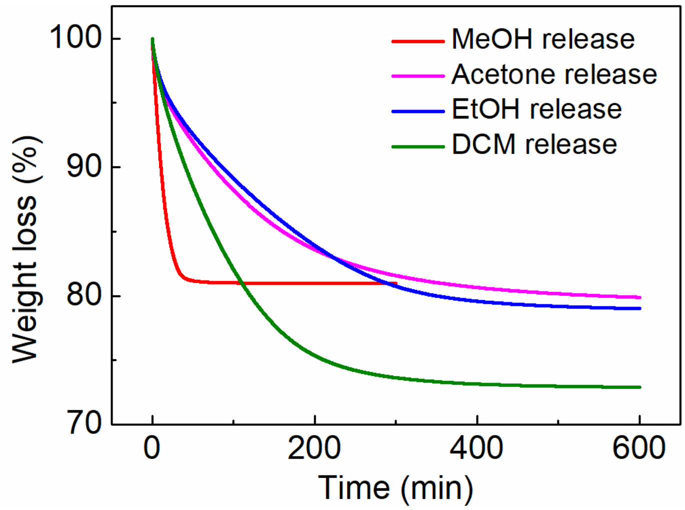

Supplementary Fig. 4 The weight loss as a function of time at 298 K for MeOH/acetone/EtOH/DCM-exchanged ZIF-65(Zn)-I. [Note: dichloromethane (DCM)]

Compared to EtOH, more polar acetone has a strong host-guest interaction with ZIF-65(Zn)-I, which leads to structural transition even if the acetone molecules leave the ZIF-65(Zn)-I structure slowly (Supplementary Fig. 4). Thus, relatively weak polarity EtOH and DCM are effective exchange solvents and the corresponding slow solvent release strategy can effectively activate the ZIF-65(Zn)-I without structural transition.

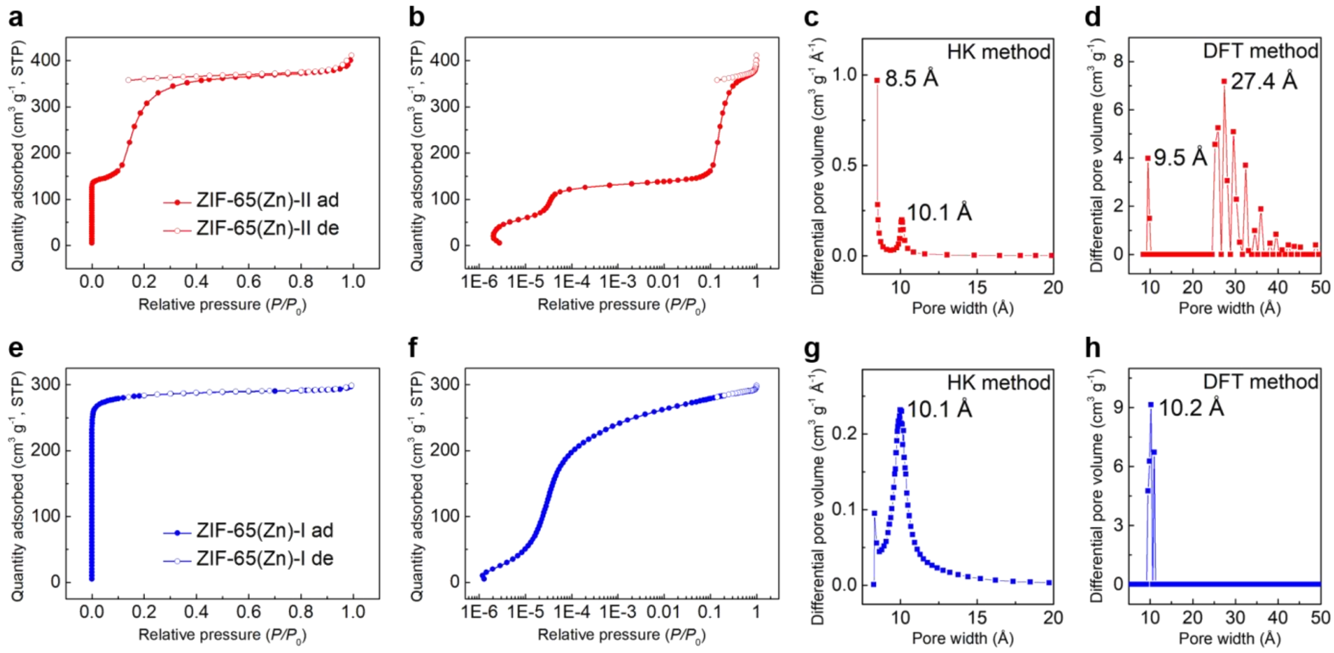

Supplementary Fig. 5 N_2 adsorption isotherms and pore size distribution curves of ZIF-65(Zn). a Linear curve and **b** logarithmic curve of N_2 adsorption isotherm of ZIF-65(Zn)-II, and **c** HK and **d** DFT pore size distribution curves of ZIF-65(Zn)-II; **e** Linear curve and **f** logarithmic curve of N_2 adsorption isotherm of ZIF-65(Zn)-I, and **g** HK and **h** DFT pore size distribution curves of ZIF-65(Zn)-I. [ZIF-65(Zn)-II: red; ZIF-65(Zn)-I: blue.]

Notably, ZIF-65(Zn)-I was recovered by immersing the ZIF-65(Zn)-II sample in DMF. The occurrence of such a structural transition was also hinted at by the observation of a stepped N_2 adsorption isotherm at 77 K for ZIF-65(Zn)-II (Supplementary Fig. 5b). Two distinctive steps were separated in the relative pressure (P/P_0) range of 1×10^{-5}–1×10^{-4} and 0.1–0.4, respectively.

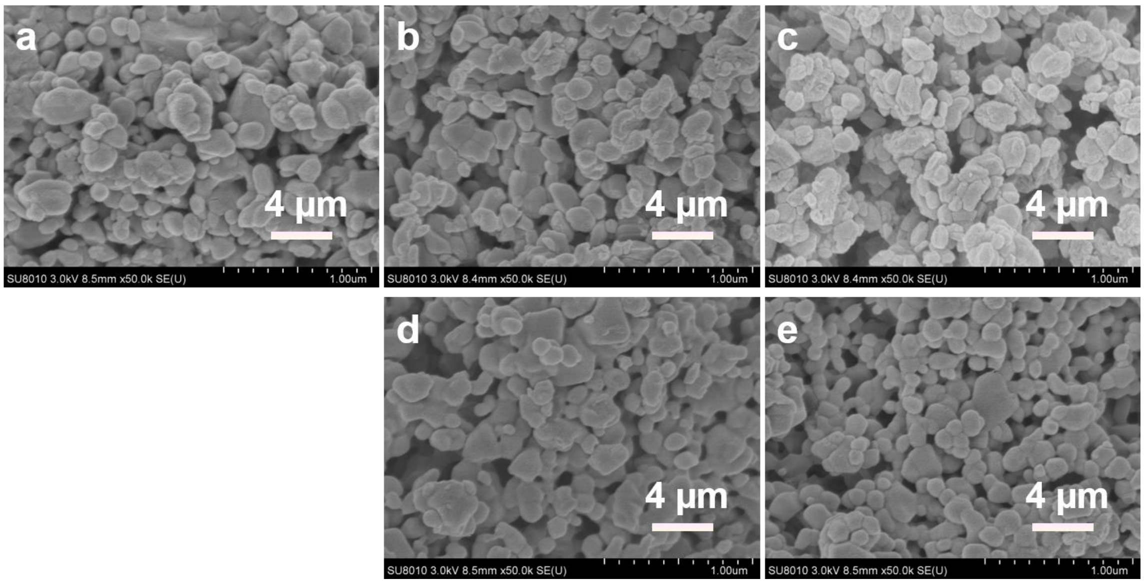

Supplementary Fig. 6 SEM images of ZIF-65(Zn). a As-synthesized, **b** MeOH exchanged (wet), **c** MeOH exchanged (dry), **d** EtOH exchanged (wet), and **e** EtOH exchanged (dry) ZIF-65(Zn).

S2.2 The structural analysis of ZIF-65(Zn)

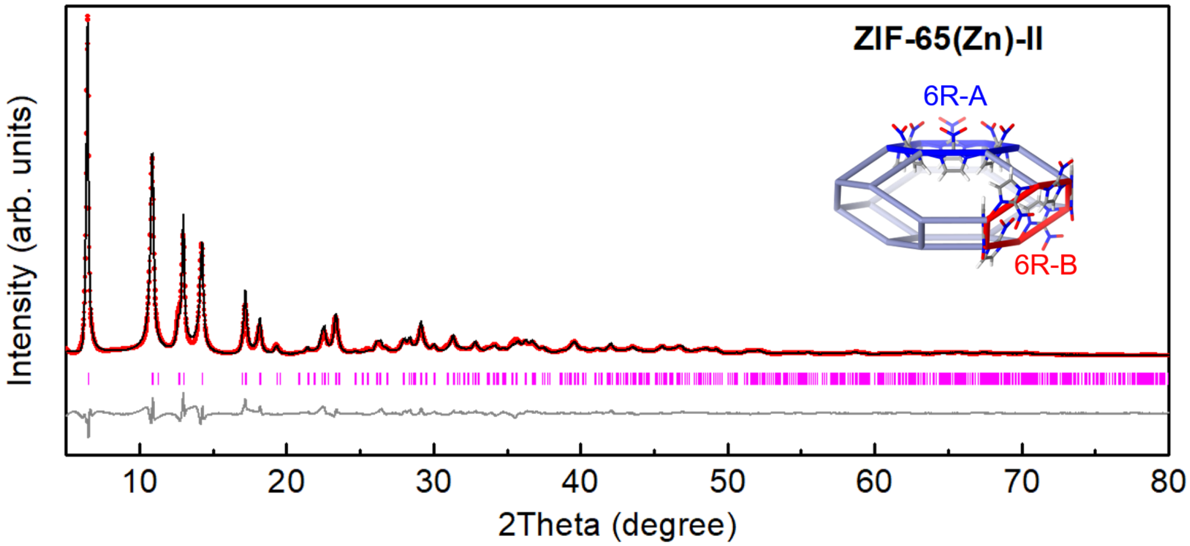

Supplementary Fig. 7 The Rietveld refinement for **ZIF-65(Zn)-II**. Red, experimental pattern; black, calculated pattern; grey, difference pattern (ex-cal); magenta, bragg peaks.

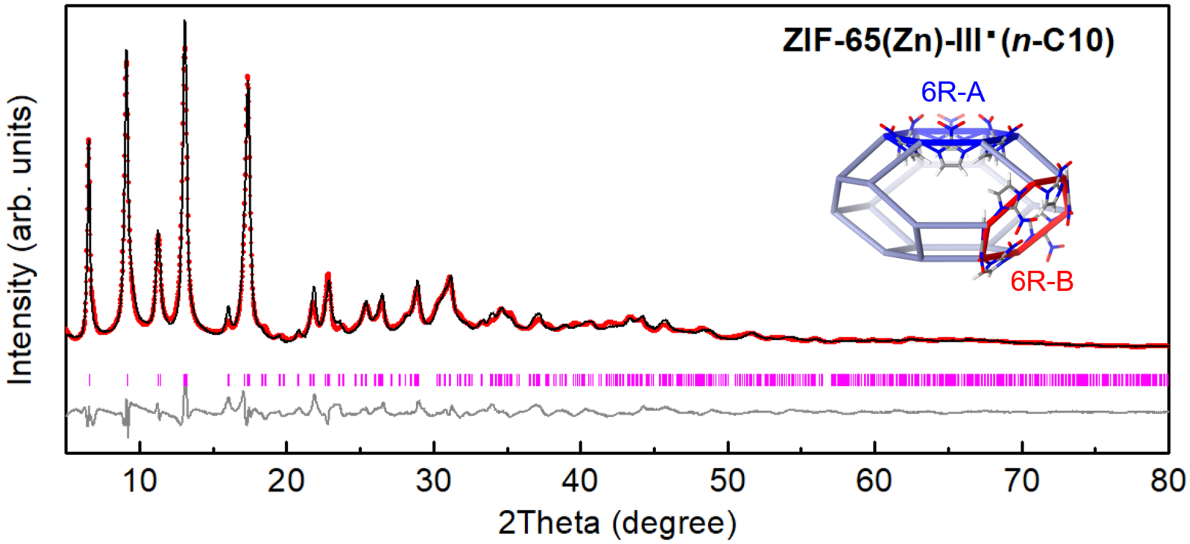

Supplementary Fig. 8 The Rietveld refinement for **ZIF-65(Zn)-III·(*n*-C10)**. Red, experimental pattern; black, calculated pattern; grey, difference pattern (ex-cal); magenta, bragg peaks.

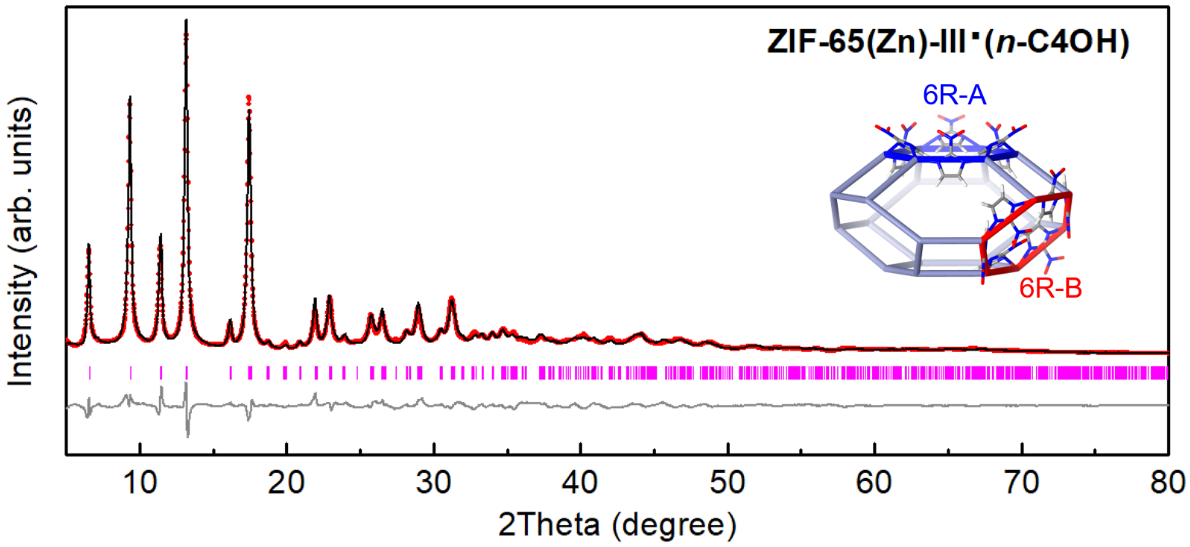

Supplementary Fig. 9 The Rietveld refinement for **ZIF-65(Zn)-III·(*n*-C4OH)**. Red, experimental pattern; black, calculated pattern; grey, difference pattern (ex-cal); magenta, bragg peaks.

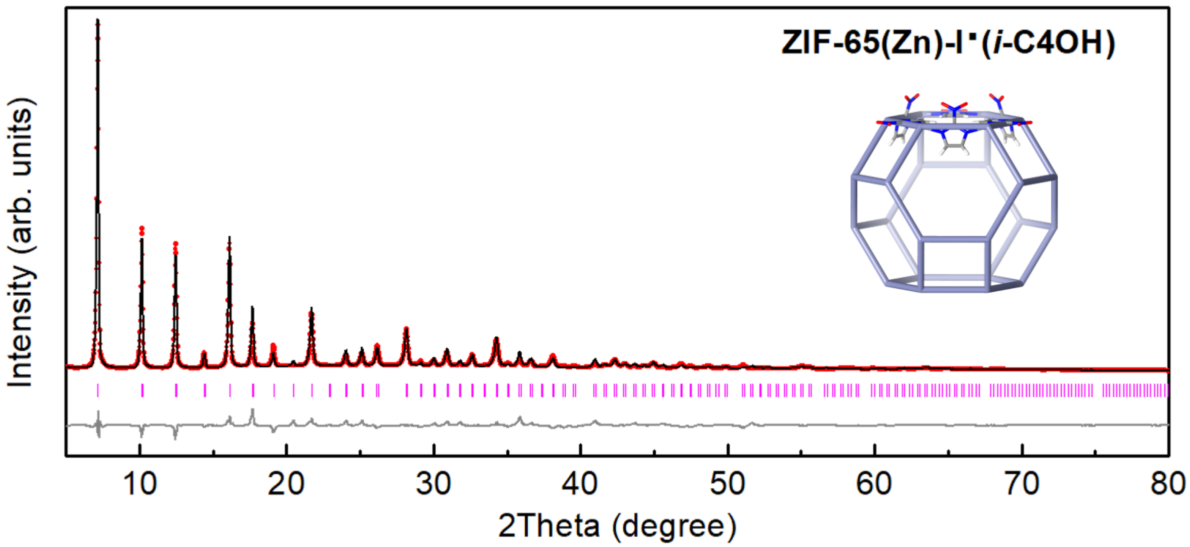

Supplementary Fig. 10 The Rietveld refinement for **ZIF-65(Zn)-I·(*i*-C4OH)**. Red, experimental pattern; black, calculated pattern; grey, difference pattern (ex-cal); magenta, bragg peaks.

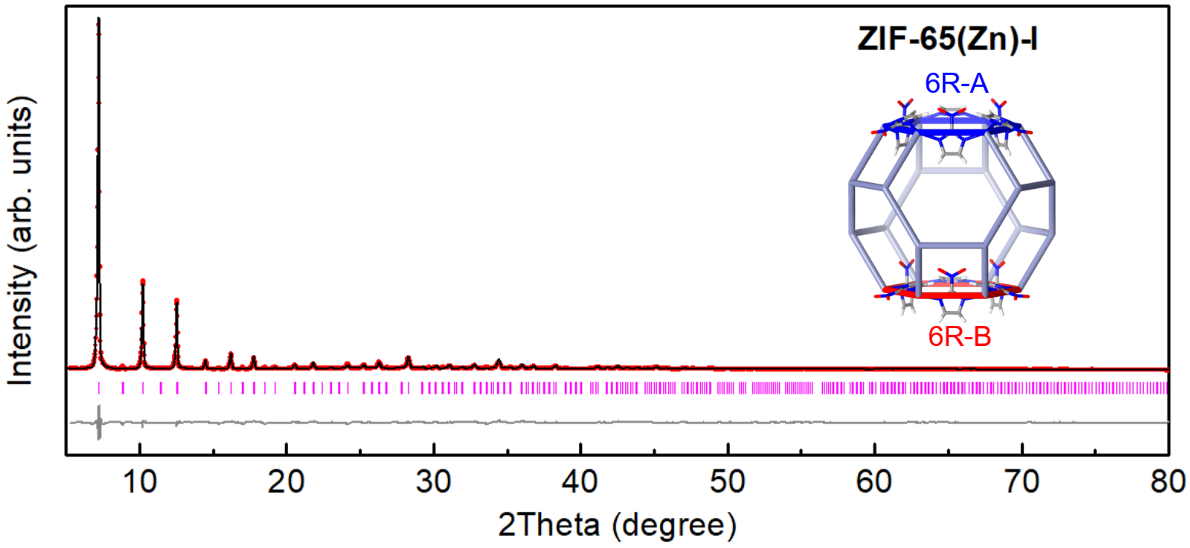

Supplementary Fig. 11 The Rietveld refinement for **ZIF-65(Zn)-I**. Red, experimental pattern; black, calculated pattern; grey, difference pattern (ex-cal); magenta, bragg peaks.

Supplementary Table 2 The crystal structure data and details of the Rietveld refinement for ZIF-65(Zn)-II, ZIF-65(Zn)-III, and ZIF-65(Zn)-I.

**Compound**	**ZIF-65(Zn)-II**	**ZIF-65(Zn)-III·(*n*-C10)**	**ZIF-65(Zn)-III·(*n*-C4OH)**	**ZIF-65(Zn)-I·(*i*-C4OH)**	**ZIF-65(Zn)-I**
Formula	$C_6H_4N_6O_4Zn$	$C_6H_4N_6O_4Zn\cdot 0.17C_{10}H_{22}$	$C_6H_4N_6O_4Zn\cdot 0.64C_4H_9OH$	$C_6H_4N_6O_4Zn\cdot 1.3C_4H_9OH$	$C_6H_4N_6O_4Zn$
Crystal system	Trigonal	Trigonal	Trigonal	Cubic	Cubic
Space group	*R*3*m*	*R*3*m*	*R*3*m*	*I*-43*m*	*P*-43*m*
a (Å)	27.163	26.936	27.005	17.315	17.327
c (Å)	8.633	10.636	10.374	–	–
V (Å^3)	5516.6	6683.2	6551.8	5191.6	5202.2
R_p[a] (%)	5.84	5.22	6.39	7.86	6.59
R_{wp}[b] (%)	7.77	6.79	8.02	10.75	9.62

R_p[a] $= \Sigma|cY^{sim}(2\theta_i) - I^{exp}(2\theta_i) + Y^{back}(2\theta_i)|/\Sigma|I^{exp}(2\theta_i)|$

R_{wp}[b] $= \{w_p[cY^{sim}(2\theta_i) - I^{exp}(2\theta_i) + Y^{back}(2\theta_i)]^2 / \Sigma w_p [I^{exp}(2\theta_i)]^2\}^{1/2}$, and $w_p = 1/I^{exp}(2\theta_i)$

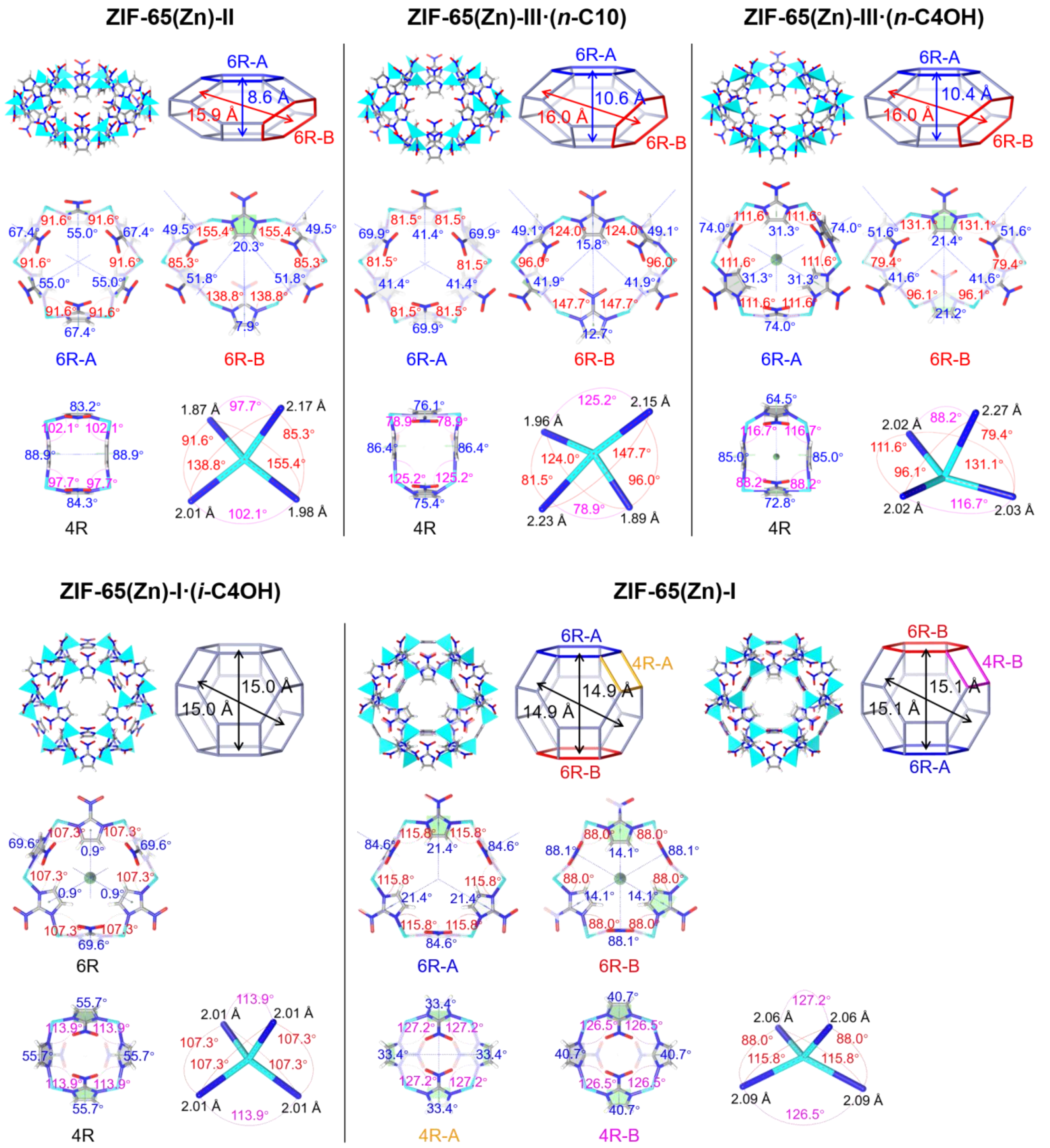

Supplementary Fig. 12 Structure analysis of ZIF-65(Zn)-II, ZIF-65(Zn)-III·(*n*-C10), ZIF-65(Zn)-III·(*n*-C4OH), ZIF-65(Zn)-I·(*i*-C4OH), and ZIF-65(Zn)-I. top) polyhedron representation and topological net of the **SOD** cage and cage size based on the distance between the face-to-face 6R planes, **middle)** six-membered rings (6R) and corresponding N–Zn–N bond angle (red) and the linker nIm-6R plane included angle (blue); **bottom)** four-membered rings (4R) and corresponding N–Zn–N bond angle (magenta) and the linker nIm-4R plane included angle (blue), and all N–Zn–N bond angles and Zn–N bond lengths (black). Zn: cyan; C: gray; N: blue; O: red; H: white. [Note: *n*-decane (*n*-C10), *n*-butanol (*n*-C4OH), isobutanol (*i*-C4OH).]

Supplementary Table 3 Structural data of ZIF-65(Zn)-II, ZIF-65(Zn)-III·(*n*-C10), ZIF-65(Zn)-III·(*n*-C4OH), ZIF-65(Zn)-I·(*i*-C4OH), and ZIF-65(Zn)-I.

**Compound**	**ZIF-65(Zn)-II**	**ZIF-65(Zn)-III·(*n*-C10)**	**ZIF-65(Zn)-III·(*n*-C4OH)**	**ZIF-65(Zn)-I·(*i*-C4OH)**	**ZIF-65(Zn)-I**
ρ [a] (g cm^{-3})	1.569	1.295	1.321	1.111	1.111
S [a] (m^2 g^{-1})	177.6	634.0	616.0	1176.4	1254.7
V_f [a] (cm^3 g^{-1})	0.104	0.264	0.273	0.447	0.459
Aperture size [b] of 6R-A (Å)	4.1	4.3	4.3	3.6	4.6
Aperture size [b] of 6R-B (Å)	1.5 × 7.6	1.6 × 6.3	0.4 × 6.9	–	3.5
Pore size [b] of cage (Å)	5.2 × 12.5	7.2 × 12.6	7.0 × 12.6	11.6 × 11.6	cage 1: 11.2 × 11.2<br>cage 2: 11.2 × 11.2
**SOD-cage size [c] (Å)**	**8.6 × 15.9**	**10.6 × 16.0**	**10.4 × 16.0**	**15.0 × 15.0**	**cage 1: 14.9 × 14.9**<br>**cage 2: 15.1 × 15.1**
**Bond length of Zn–N (Å)**	**1.87–2.17**	**1.89–2.23**	**2.02–2.27**	**2.01**	**2.06–2.09**
**Bond angle of N–Zn–N in 6R (°)**	**85.3–155.4**	**81.5–147.7**	**79.4–131.1**	**107.3**	**88.0–115.8**
Bond angle of N–Zn–N in 4R (°)	97.7–102.1	78.9–125.2	88.2–116.7	113.9	126.5–127.2
Included angle of nIm–6R (°)	8.0–67.4	12.7–69.9	21.2–74.0	0.9–69.6	14.1–88.1
Included angle of nIm–4R (°)	83.3–89.3	75.4–86.4	64.5–85.0	55.7	33.4–40.7

[a] Computational data of perfect crystals;

[b] Excluding the radius of the specific atom;

[c] The distance between the face-to-face 6R planes (min and max value).

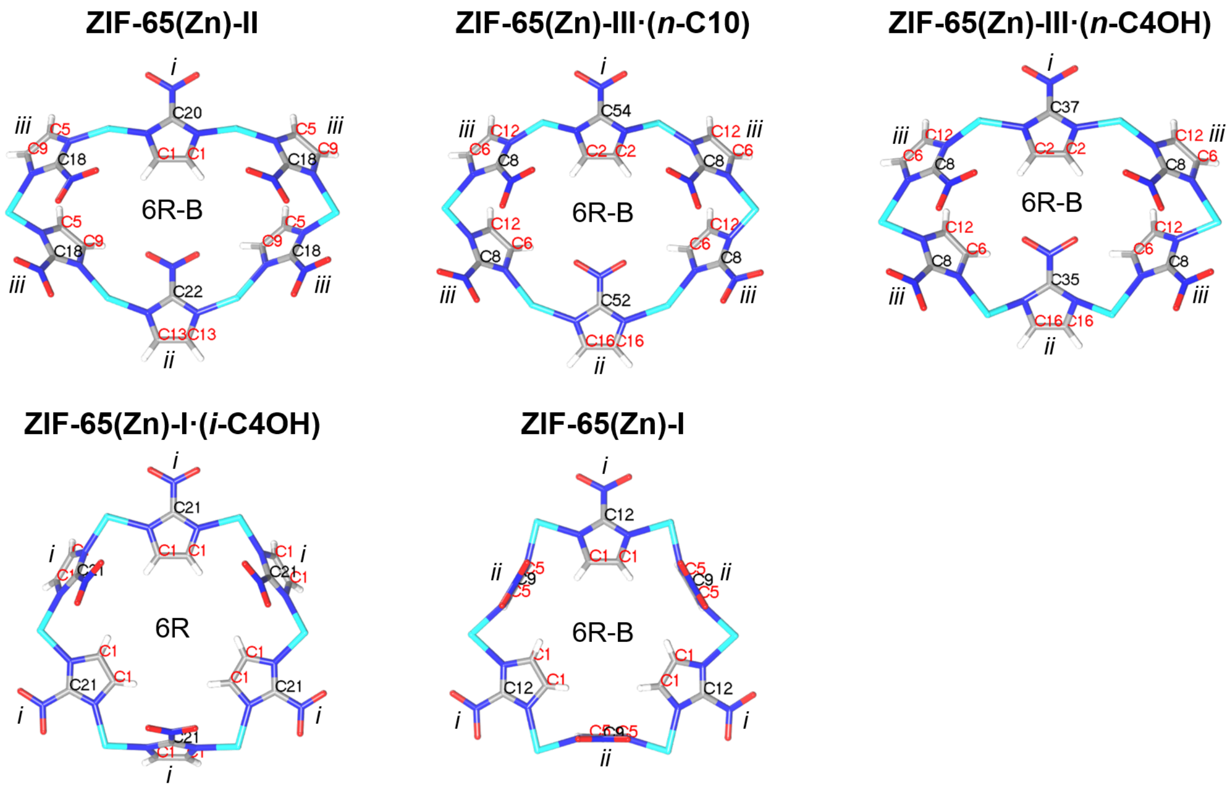

Supplementary Fig. 13 Conformation of nIm for ZIF-65(Zn)-II, ZIF-65(Zn)-III·(*n*-C10), ZIF-65(Zn)-III·(*n*-C4OH), ZIF-65(Zn)-I·(*i*-C4OH), and ZIF-65(Zn)-I.

Supplementary Table 4 The detailed conformation of nIm for Supplementary Fig. 13.

	**ZIF-65(Zn)-II**		**ZIF-65(Zn)-III·(*n*-C10)**		**ZIF-65(Zn)-III·(*n*-C4OH)**	
**Conformation of nIm**	**2-**	**4,5-**	**2-**	**4,5-**	**2-**	**4,5-**
i (symmetric nIm)	C20	C1, C1	C54	C2, C2	C37	C2, C2
ii (symmetric nIm)	C22	C13, C13	C52	C16, C16	C35	C16, C16
iii (asymmetric nIm)	C18	C5, C9	C8	C6, C12	C8	C6, C12
	**ZIF-65(Zn)-I·(*i*-C4OH)**		**ZIF-65(Zn)-I**			
**Conformation of nIm**	**2-**	**4,5-**	**2-**	**4,5-**		
i (symmetric nIm)	C21	C1, C1	C12	C1, C1		
ii (symmetric nIm)			C9	C5, C5		

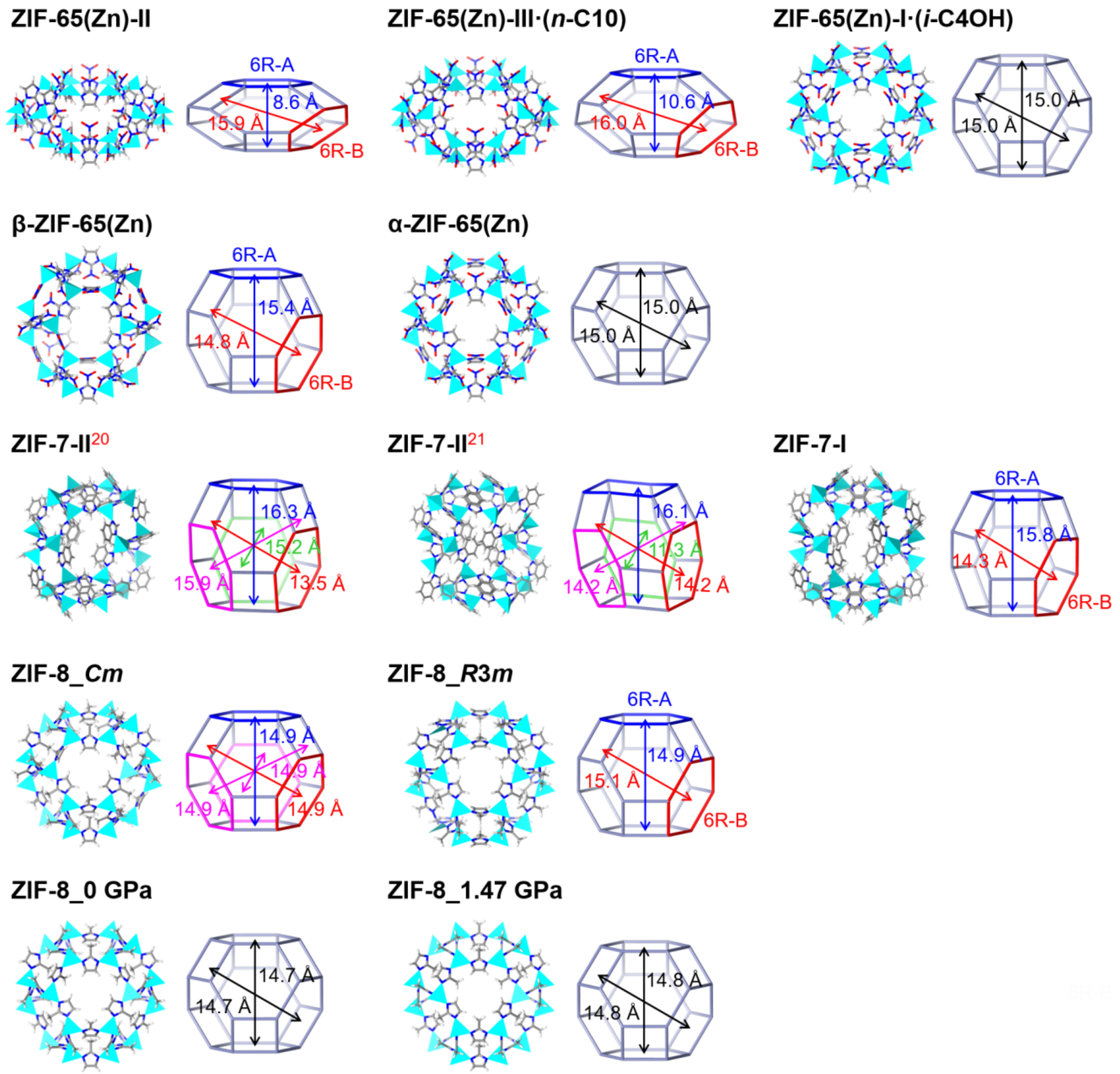

Supplementary Fig. 14 Comparison of the cage of flexible ZIF-65(Zn) and other flexible ZIFs.

Supplementary Table 5 Comparison of the structural properties of flexible ZIF-65(Zn) and other flexible ZIFs.

Compound	Crystal system	Space group	ρ (g cm^{-3})	S (m^2 g^{-1})	V (cm^3 g^{-1})	**SOD-cage size [a] (Å)**	Ref
ZIF-65(Zn)-II	Trigonal	*R*3*m*	1.569	177.6	0.104	**8.6 × 15.9**	this work
ZIF-65(Zn)-III·(*n*-C10)	Trigonal	*R*3*m*	1.295	634.0	0.264	**10.6 × 16.0**	this work
ZIF-65(Zn)-I·(*i*-C4OH)	Cubic	*I*-43*m*	1.111	1176.4	0.447	**15.0 × 15.0**	this work
β-ZIF-65(Zn)[3]	Cubic	*IA*-3	1.124	1191.4	0.448	**14.8 × 15.4**	3
α-ZIF-65(Zn)[3]	Cubic	*I*-43*m*	1.114	1152.4	0.446	**15.0 × 15.0**	3
ZIF-7-II[20]	Triclinic	*P*-1	1.131	364.2	0.280	**13.5 × 16.3**	20
ZIF-7-II[21]	Triclinic	*P*-1	1.519	0	0.007	**11.3 × 16.1**	21
ZIF-7-I[22]	Trigonal	*R*-3	1.241	243.2	0.187	**14.3 × 15.8**	22
ZIF-8_*Cm*[23]	Monoclinic	*Cm*	0.894	1429.9	0.563	**14.9 × 14.9**	23
ZIF-8_*R*3*m*[23]	Trigonal	*R*3*m*	0.885	1480.8	0.569	**14.9 × 15.1**	23
ZIF-8_0 GPa[24]	Cubic	*I*-43*m*	0.925	1364.8	0.537	**14.7 × 14.7**	24
ZIF-8_1.47 GPa[24]	Cubic	*I*-43*m*	0.912	1401.1	0.549	**14.8 × 14.8**	24

[a] The distance between the face-to-face 6R planes (min and max value).

Supplementary Table 6 Comparison of the selected bond length, bond angle, and included angle of flexible ZIF-65(Zn) and other flexible ZIFs (The min–max value for length and angle are listed).

Compound	Bond length of Zn–N (Å)	**Bond angle of N–Zn–N in 6R (°)**	Bond angle of N–Zn–N in 4R (°)	Included angle of nIm–6R (°)	Included angle of nIm–4R (°)
ZIF-65(Zn)-II	1.87–2.17	**85.3–155.4**	97.7–102.1	8.0–67.4	83.3–89.3
ZIF-65(Zn)-III·(*n*-C10)	1.89–2.23	**81.5–147.7**	78.9–125.2	12.7–69.9	75.4–86.4
ZIF-65(Zn)-I·(*i*-C4OH)	2.01	**107.3**	113.9	0.9–69.6	55.7
β-ZIF-65(Zn)[3]	2.02–2.04	**99.6–103.2**	123.7–127.6	10.0–89.2	38.0–40.0
α-ZIF-65(Zn)[3]	2.02	**104.3**	120.5	3.3–73.9	51.4
ZIF-7-II[20]	1.93–2.24	**91.2–120.6**	87.3–123.1	12.4–73.6	2.7–87.6
ZIF-7-II[21]	1.93–2.06	**100.1–119.7**	100.1–116.4	26.9–86.9	6.5–75.5
ZIF-7-I[22]	1.98–2.00	**107.7–115.2**	103.6–105.3	22.4–64.0	4.3–64.0
ZIF-8_*Cm*[23]	1.99–2.02	**102.2–117.1**	108.6–113.1	7.1–76.7	22.7–71.8
ZIF-8_*R*3*m*[23]	1.99–2.03	**103.3–115.8**	108.6–112.8	4.6–69.3	16.8–80.9
ZIF-8_0 GPa[24]	1.99	**109.4**	109.7	10.5–60.0	65.2
ZIF-8_1.47 GPa[24]	1.99	**110.1**	108.3	33.5-37.0	88.3

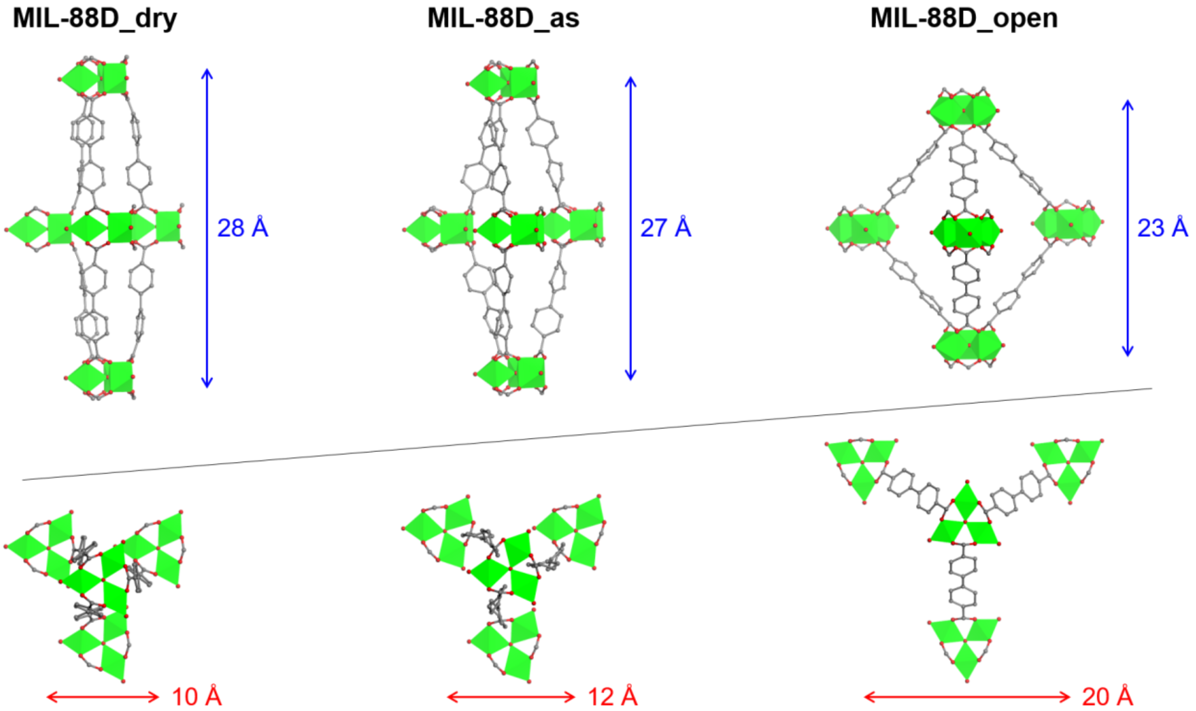

Supplementary Fig. 15 Scheme of the large flexibility of MIL-88D: the evolution of the bipyramidal cage. (dry for dehydrated, as for as-synthesized, open for the filled cage.)

S2.3 The liquid adsorption of ZIF-65(Zn)

Supplementary Table 7 Physical properties of studied alkanes, alcohols, aldehydes, and ketones.

Adsorbate		Molecular size (Å)	Polarizability (Å^3)	Dipole moment (debye)	P_0 (kPa)
Full name	Abbreviation				
n-hexane	*n*-C6	10.6 × 4.6 × 4.2	10.76	0	20.266
n-heptane	*n*-C7	11.9 × 4.6 × 4.2	12.53	0.06	6.066
n-octane	*n*-C8	13.2 × 4.6 × 4.2	14.30	0	1.871
n-nonane	*n*-C9	14.4 × 4.6 × 4.2	16.08	0.06	0.580
n-decane	*n*-C10	15.7 × 4.6 × 4.2	17.86	0	0.181
2-methylpentane	2-MeC5	9.3 × 6.6 × 5.2	10.71	0.10	28.149
2-methylhexane	2-MeC6	10.7 × 6.5 × 5.4	12.40	0.06	8.775
2-methylheptane	2-MeC7	11.2 × 6.8 × 5.4	14.18	0.12	2.881
2-methyloctane	2-MeC8	12.3 × 6.8 × 5.4	15.96	0.08	0.924
2-methylnonane	2-MeC9	13.7 × 7.1 × 5.4	17.67	0.13	0.272
2,2-dimethylbutane	2,2-diMeC4	8.1 × 6.8 × 5.7	10.76	0.04	42.138
2,2,3-trimethylbutane	2,2,3-triMeC4	7.5 × 6.8 × 6.2	12.41	0.03	13.458
2,2,4-trimethylpentane	2,2,4-triMeC5	9.3 × 6.6 × 6.2	14.27	0.10	6.576
2,2,4-trimethylhexane	2,2,4-triMeC6	9.0 × 7.5 × 6.6	15.87	0.11	2.117
4,4-dimethyloctane	4,4-diMeC8	12.0 × 6.7 × 6.2	17.48	0.08	0.487
cyclohexane	*c*-C6	7.4 × 6.7 × 5.1	10.07	0	13.174
ethanol	EtOH	6.5 × 4.6 × 4.2	4.43	1.54	7.928
n-propanol	*n*-C3OH	7.8 × 4.6 × 4.2	6.13	1.47	2.753
n-butanol	*n*-C4OH	9.0 × 4.6 × 4.2	7.88	1.51	0.902
n-pentanol	*n*-C5OH	10.3 × 4.6 × 4.2	9.63	1.45	0.330
n-hexanol	*n*-C6OH	11.6 × 4.6 × 4.2	11.39	1.50	0.098
n-heptanol	*n*-C7OH	12.9 × 4.6 × 4.2	13.16	1.45	0.029
n-octanol	*n*-C8OH	14.2 × 4.6 × 4.2	14.93	1.50	0.011
n-nonanol	*n*-C9OH	15.5 × 4.6 × 4.2	16.71	1.44	0.003
n-decanol	*n*-C10OH	16.7 × 4.6 × 4.2	18.49	1.50	0.001
isobutanol	*i*-C4OH	7.8 × 5.5 × 5.5	7.91	1.40	1.373
tert-butanol	*t*-C4OH	6.7 × 6.2 × 5.8	8.02	1.57	5.581
cyclohexanol	*c*-C6OH	8.1 × 6.7 × 5.1	10.76	1.61	0.088
acetone	acetone	6.2 × 5.8 × 4.2	5.81	2.78	30.752
1,3-propanediol	1,3-PDO	8.8 × 4.6 × 4.2	6.76	2.99	0.004
2,3-butanediol	2,3-BDO	8.0 × 6.9 × 5.1	8.68	1.94	0.034
furfural	Fur	7.9 × 6.2 × 3.4	9.75	4.46	0.252
5-hydroxymethylfurfural	5-HMF	10.0 × 6.8 × 4.2	12.44	4.17	0.001

P_0: saturation vapor pressure at 298K.

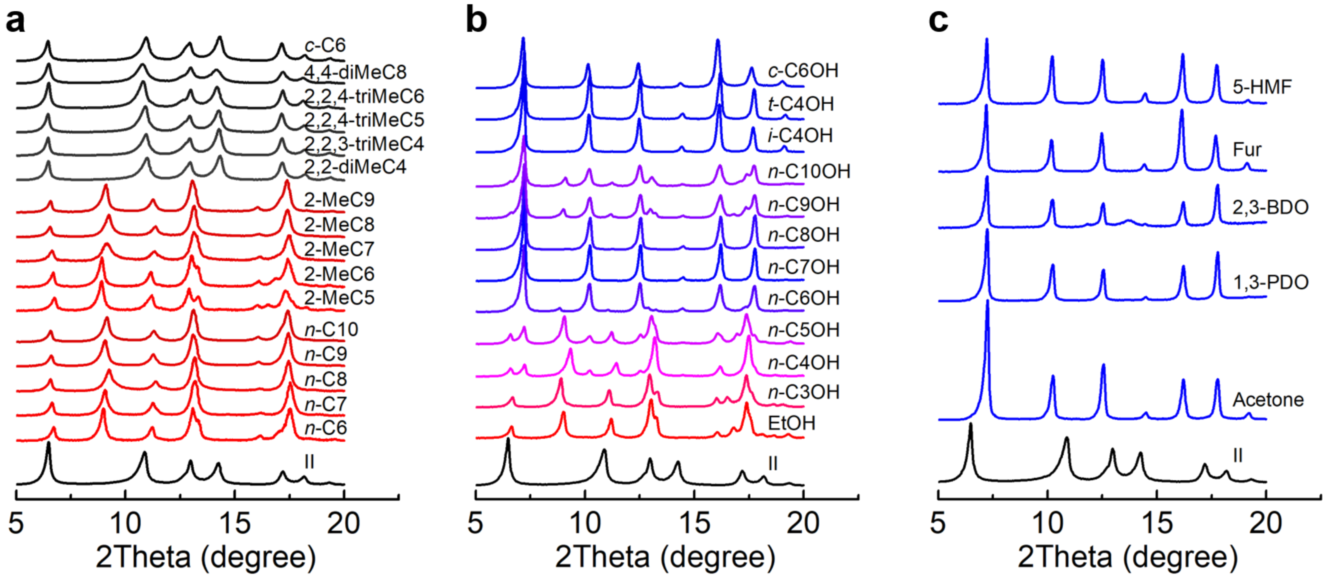

Supplementary Fig. 16 Liquid adsorption in ZIF-65(Zn)-II. PXRD of ZIF-65(Zn)-II after immersion in typical **a** alkanes, **b** mono-alcohols, and **c** poly-alcohols, aldehydes, and ketones at 298 K.

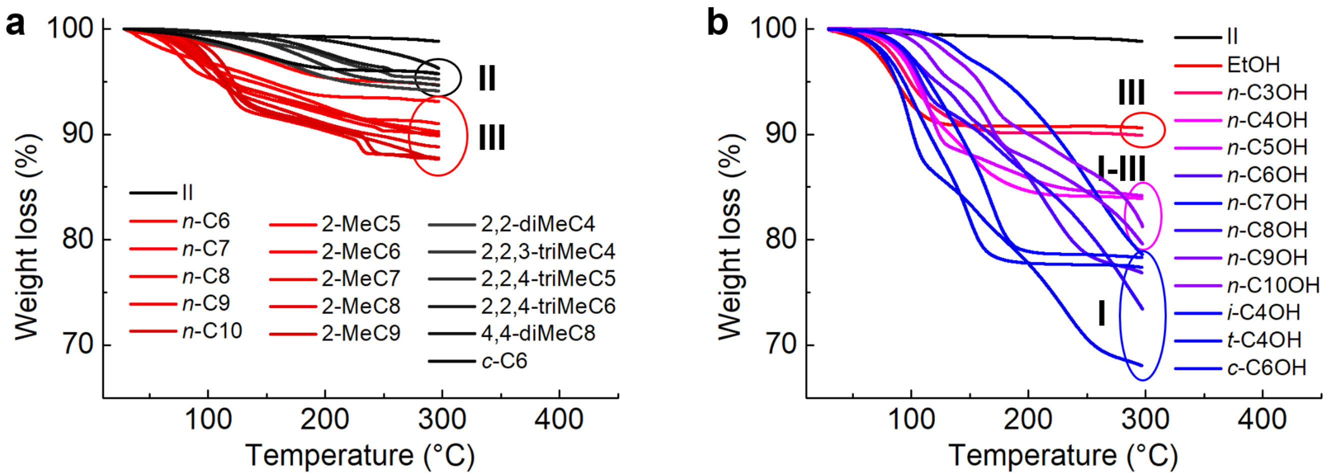

Supplementary Fig. 17 Liquid adsorption in ZIF-65(Zn)-II. TG curves after **a** alkanes, and **b** mono-alcohols adsorption in ZIF-65(Zn)-II.

S2.4 The vapor adsorption of ZIF-65(Zn)

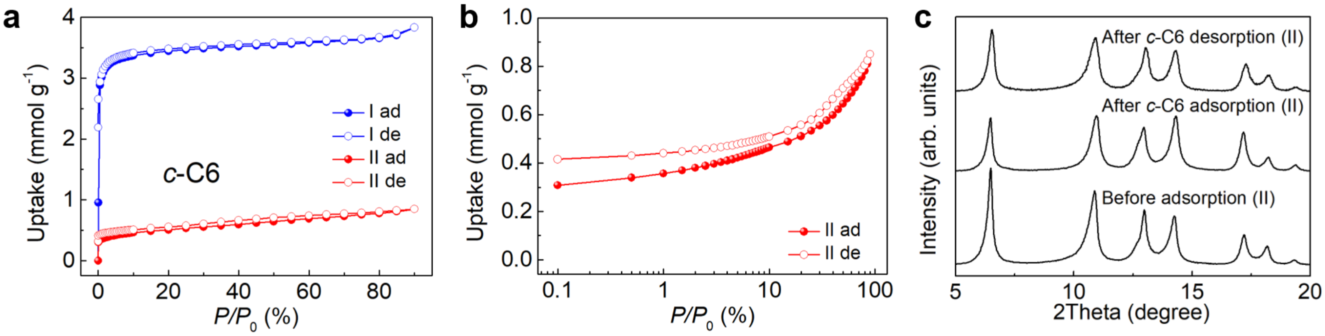

Supplementary Fig. 18 The *c*-C6 vapor adsorption-desorption isotherms in ZIF-65(Zn) and corresponding PXRD patterns. a Linear curve of *c*-C6 adsorption (solid) and desorption (empty) isotherms in ZIF-65(Zn)-II (red) and ZIF-65(Zn)-I (blue) at 298 K, **b** logarithmic curve of *c*-C6 adsorption and desorption isotherms in ZIF-65(Zn)-II. **c** PXRD patterns of ZIF-65(Zn)-II before and after *c*-C6 adsorption-desorption.

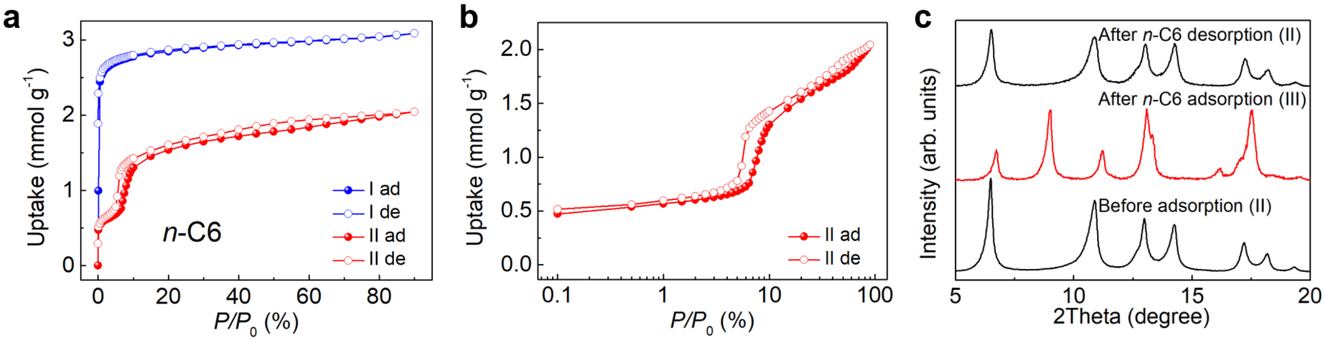

Supplementary Fig. 19 The *n*-C6 vapor adsorption-desorption isotherms in ZIF-65(Zn) and corresponding PXRD patterns. a Linear curve of *n*-C6 adsorption (solid) and desorption (empty) isotherms in ZIF-65(Zn)-II (red) and ZIF-65(Zn)-I (blue) at 298 K, **b** logarithmic curve of *n*-C6 adsorption and desorption isotherms in ZIF-65(Zn)-II. **c** PXRD patterns of ZIF-65(Zn)-II before and after *n*-C6 adsorption-desorption.

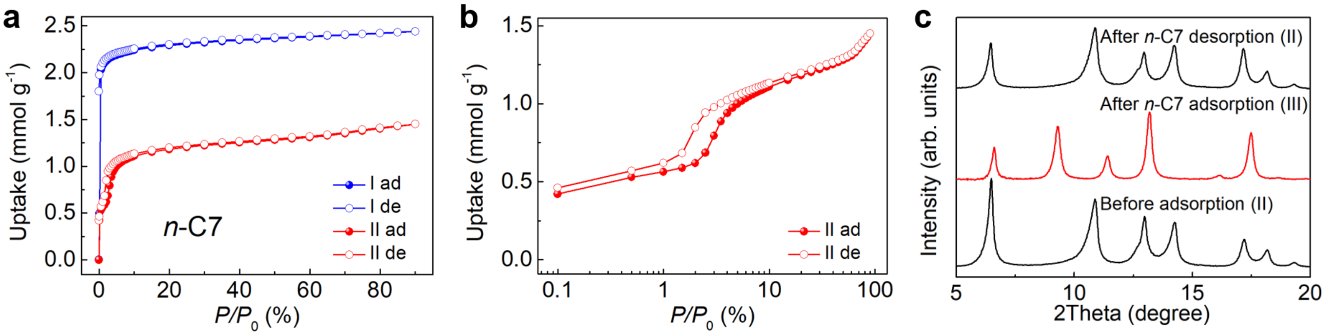

Supplementary Fig. 20 The *n*-C7 vapor adsorption-desorption isotherms in ZIF-65(Zn) and corresponding PXRD patterns. a Linear curve of *n*-C7 adsorption (solid) and desorption (empty) isotherms in ZIF-65(Zn)-II (red) and ZIF-65(Zn)-I (blue) at 298 K, **b** logarithmic curve of *n*-C7 adsorption and desorption isotherms in ZIF-65(Zn)-II. **c** PXRD patterns of ZIF-65(Zn)-II before and after *n*-C7 adsorption-desorption.

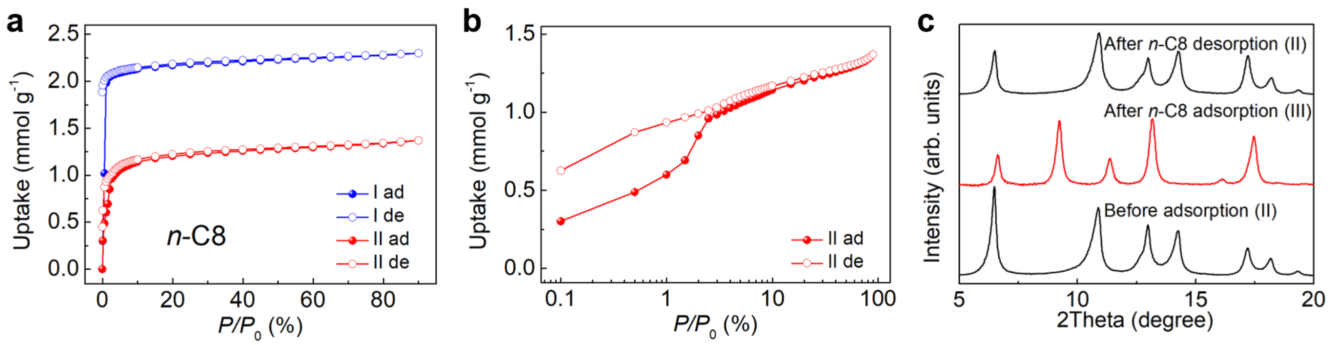

Supplementary Fig. 21 The *n*-C8 vapor adsorption-desorption isotherms in ZIF-65(Zn) and corresponding PXRD patterns. a Linear curve of *n*-C8 adsorption (solid) and desorption (empty) isotherms in ZIF-65(Zn)-II (red) and ZIF-65(Zn)-I (blue) at 298 K, **b** logarithmic curve of *n*-C8 adsorption and desorption isotherms in ZIF-65(Zn)-II. **c** PXRD patterns of ZIF-65(Zn)-II before and after *n*-C8 adsorption-desorption.

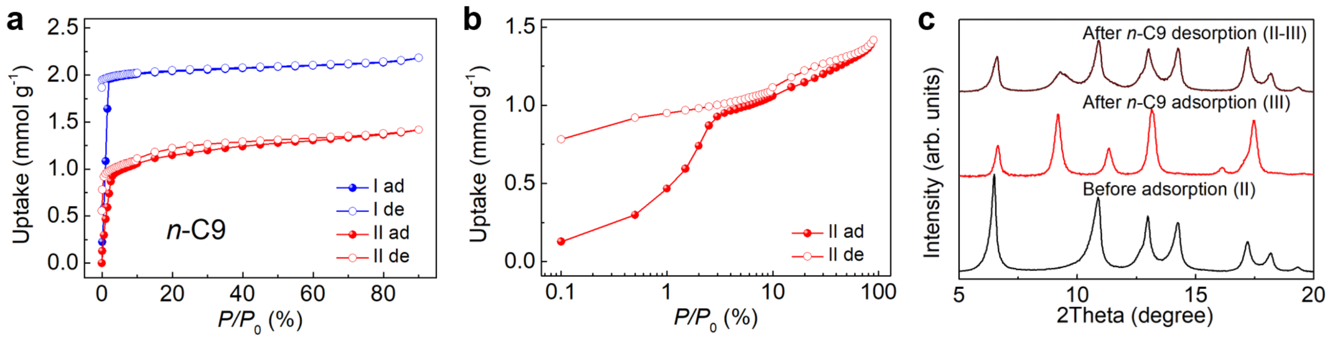

Supplementary Fig. 22 The *n*-C9 vapor adsorption-desorption isotherms in ZIF-65(Zn) and corresponding PXRD patterns. a Linear curve of *n*-C9 adsorption (solid) and desorption (empty) isotherms in ZIF-65(Zn)-II (red) and ZIF-65(Zn)-I (blue) at 298 K, **b** logarithmic curve of *n*-C9 adsorption and desorption isotherms in ZIF-65(Zn)-II. **c** PXRD patterns of ZIF-65(Zn)-II before and after *n*-C9 adsorption-desorption.

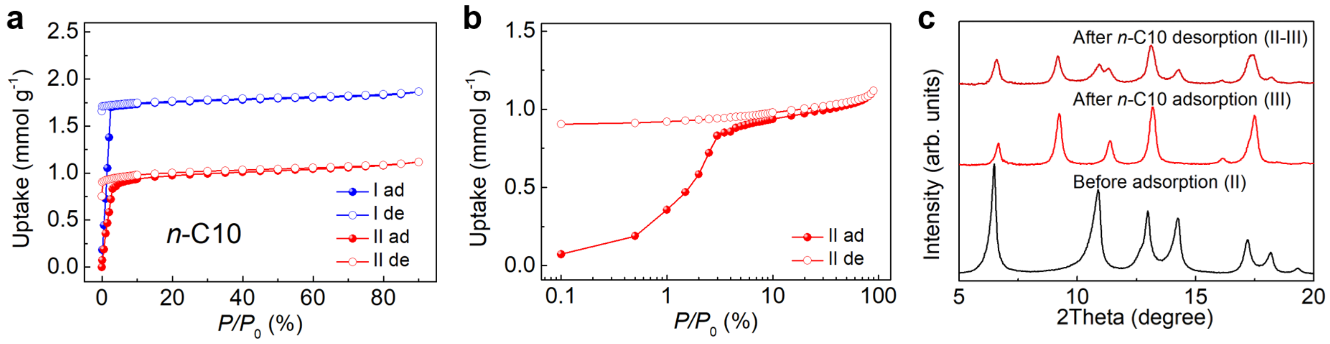

Supplementary Fig. 23 The *n*-C10 vapor adsorption-desorption isotherms in ZIF-65(Zn) and corresponding PXRD patterns. a Linear curve of *n*-C10 adsorption (solid) and desorption (empty) isotherms in ZIF-65(Zn)-II (red) and ZIF-65(Zn)-I (blue) at 298 K, **b** logarithmic curve of *n*-C10 adsorption and desorption isotherms in ZIF-65(Zn)-II. **c** PXRD patterns of ZIF-65(Zn)-II before and after *n*-C10 adsorption-desorption.

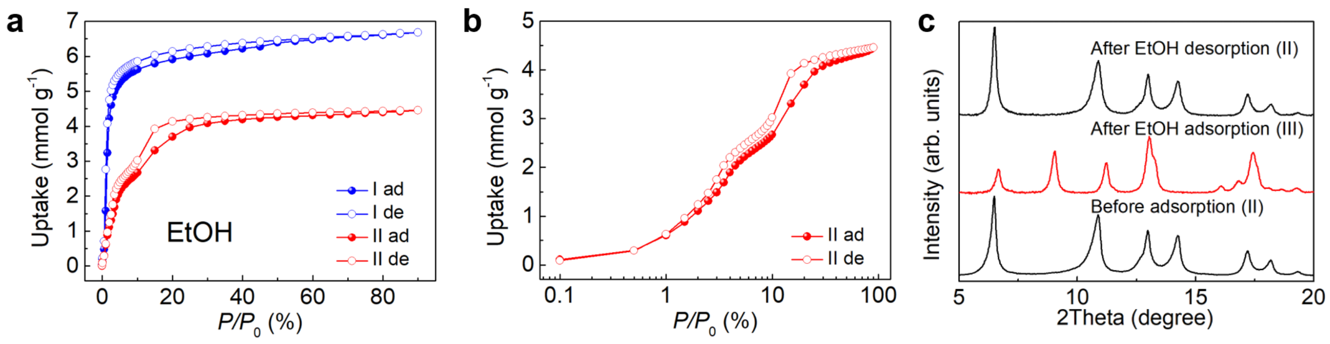

Supplementary Fig. 24 The EtOH vapor adsorption-desorption isotherms in ZIF-65(Zn) and corresponding PXRD patterns. a Linear curve of EtOH adsorption (solid) and desorption (empty) isotherms in ZIF-65(Zn)-II (red) and ZIF-65(Zn)-I (blue) at 298 K, **b** logarithmic curve of EtOH adsorption and desorption isotherms in ZIF-65(Zn)-II. **c** PXRD patterns of ZIF-65(Zn)-II before and after EtOH adsorption-desorption.

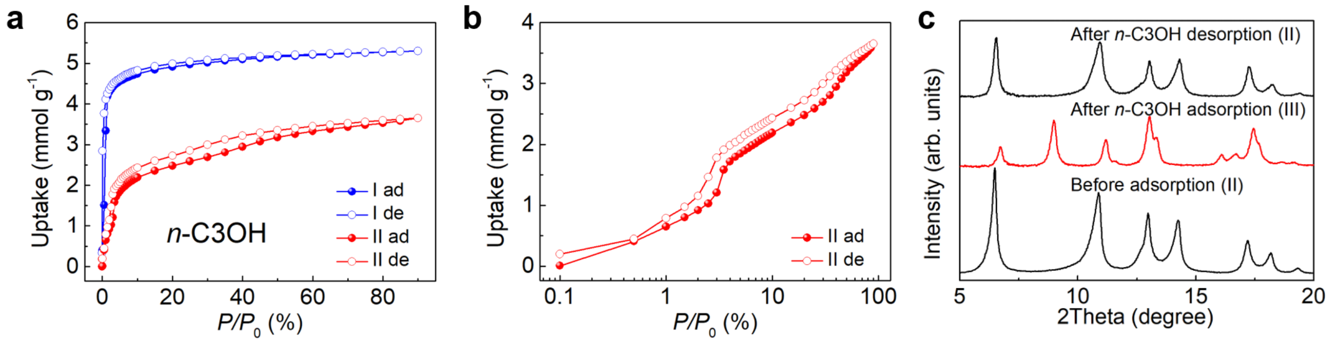

Supplementary Fig. 25 The *n*-C3OH vapor adsorption-desorption isotherms in ZIF-65(Zn) and corresponding PXRD patterns. a Linear curve of *n*-C3OH adsorption (solid) and desorption (empty) isotherms in ZIF-65(Zn)-II (red) and ZIF-65(Zn)-I (blue) at 298 K, **b** logarithmic curve of *n*-C3OH adsorption and desorption isotherms in ZIF-65(Zn)-II. **c** PXRD patterns of ZIF-65(Zn)-II before and after *n*-C3OH adsorption-desorption.

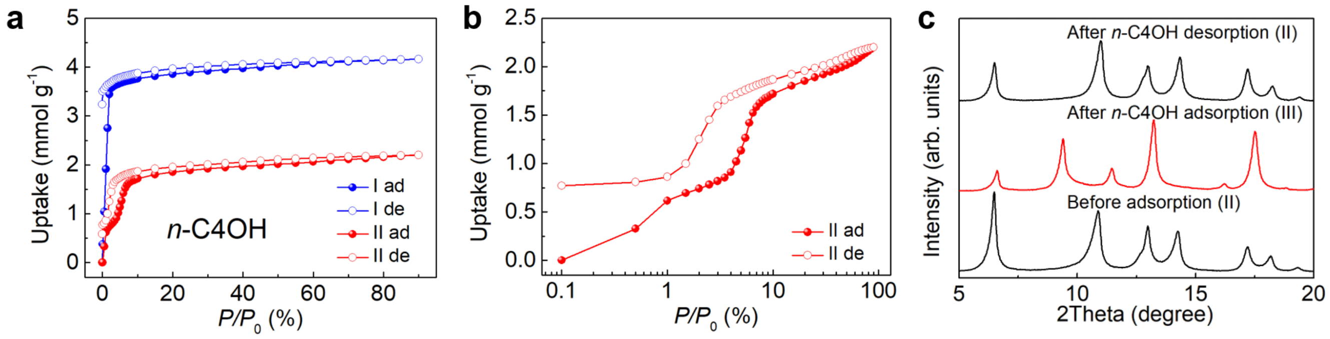

Supplementary Fig. 26 The *n*-C4OH vapor adsorption-desorption isotherms in ZIF-65(Zn) and corresponding PXRD patterns. a Linear curve of *n*-C4OH adsorption (solid) and desorption (empty) isotherms in ZIF-65(Zn)-II (red) and ZIF-65(Zn)-I (blue) at 298 K, **b** logarithmic curve of *n*-C4OH adsorption and desorption isotherms in ZIF-65(Zn)-II. **c** PXRD patterns of ZIF-65(Zn)-II before and after *n*-C4OH adsorption-desorption.

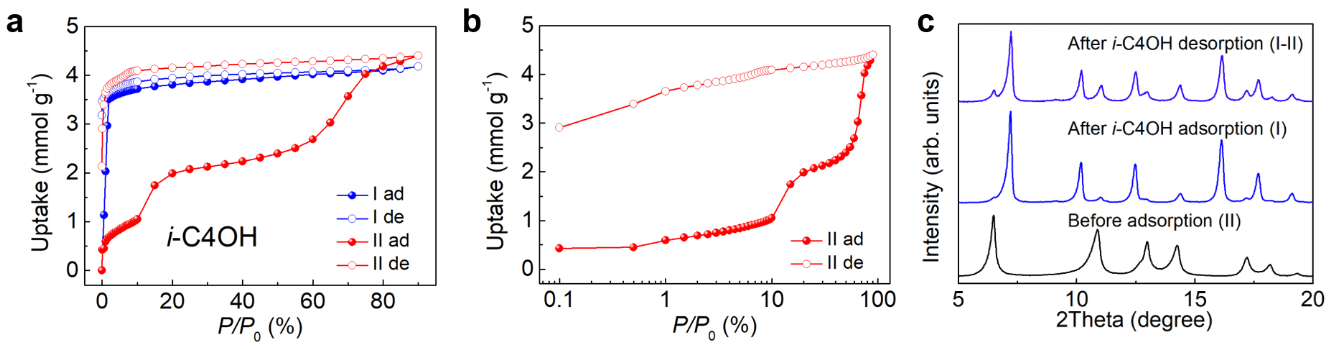

Supplementary Fig. 27 The *i*-C4OH vapor adsorption-desorption isotherms in ZIF-65(Zn) and corresponding PXRD patterns. a Linear curve of *i*-C4OH adsorption (solid) and desorption (empty) isotherms in ZIF-65(Zn)-II (red) and ZIF-65(Zn)-I (blue) at 298 K, **b** logarithmic curve of *i*-C4OH adsorption and desorption isotherms in ZIF-65(Zn)-II. **c** PXRD patterns of ZIF-65(Zn)-II before and after *i*-C4OH adsorption-desorption.

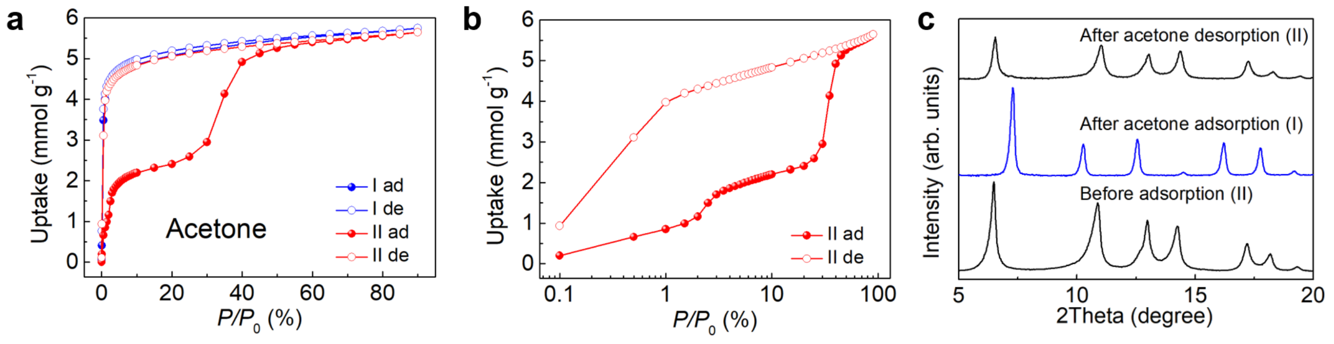

Supplementary Fig. 28 The acetone vapor adsorption-desorption isotherms in ZIF-65(Zn) and corresponding PXRD patterns. a Linear curve of acetone adsorption (solid) and desorption (empty) isotherms in ZIF-65(Zn)-II (red) and ZIF-65(Zn)-I (blue) at 298 K, **b** logarithmic curve of acetone adsorption and desorption isotherms in ZIF-65(Zn)-II. **c** PXRD patterns of ZIF-65(Zn)-II before and after acetone adsorption-desorption.

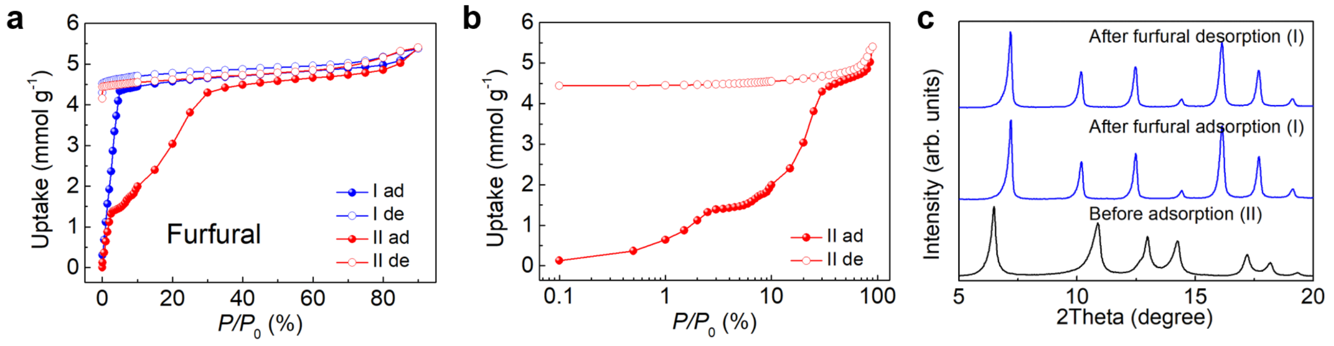

Supplementary Fig. 29 The furfural vapor adsorption-desorption isotherms in ZIF-65(Zn) and corresponding PXRD patterns. a Linear curve of furfural adsorption (solid) and desorption (empty) isotherms in ZIF-65(Zn)-II (red) and ZIF-65(Zn)-I (blue) at 298 K, **b** logarithmic curve of furfural adsorption and desorption isotherms in ZIF-65(Zn)-II. **c** PXRD patterns of ZIF-65(Zn)-II before and after furfural adsorption-desorption.

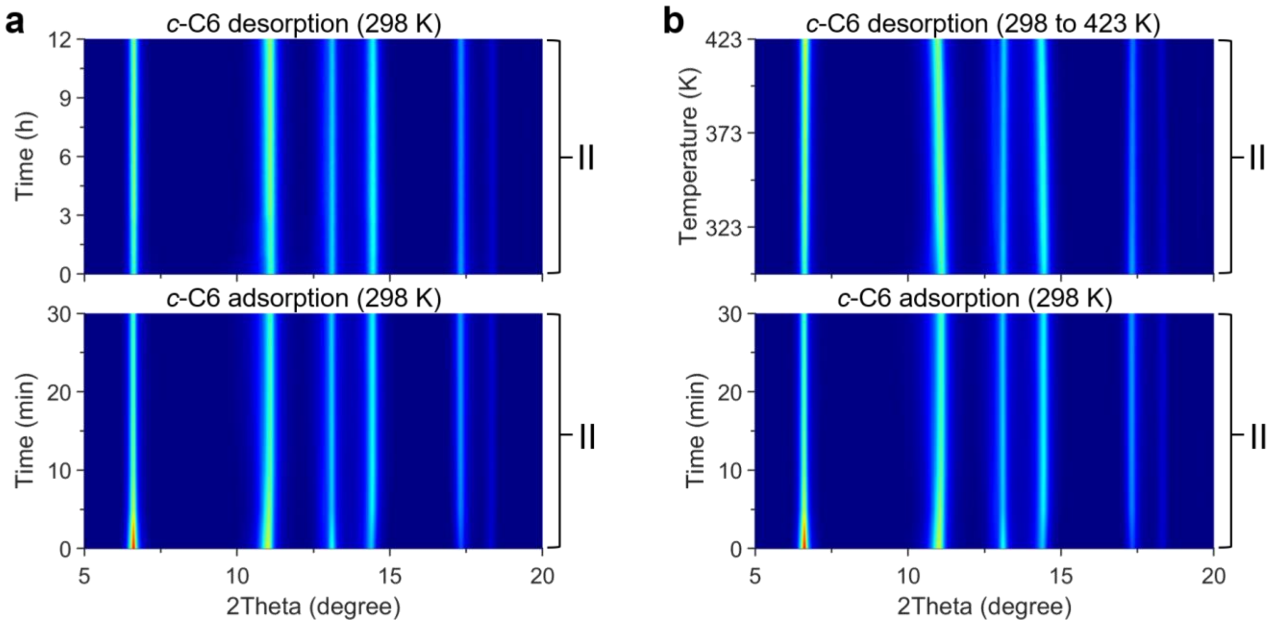

Supplementary Fig. 30 In situ PXRD for *c*-C6 vapor adsorption-desorption in ZIF-65(Zn)-II. a In situ PXRD patterns for *c*-C6 adsorption (bottom) and desorption (top) at 298 K in ZIF-65(Zn)-II, **b** in situ PXRD patterns for *c*-C6 adsorption at 298 K (bottom) and desorption from 298 to 423 K (top) in ZIF-65(Zn)-II.

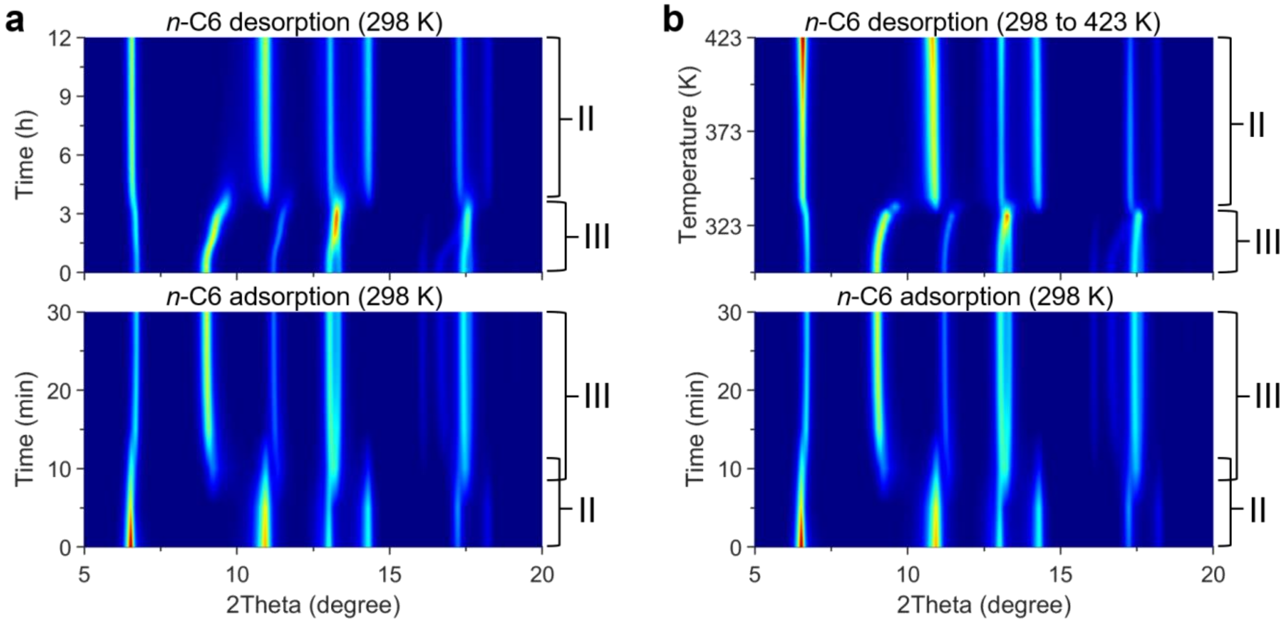

Supplementary Fig. 31 In situ PXRD for *n*-C6 vapor adsorption-desorption in ZIF-65(Zn)-II. a In situ PXRD patterns for *n*-C6 adsorption (bottom) and desorption (top) at 298 K in ZIF-65(Zn)-II, **b** in situ PXRD patterns for *n*-C6 adsorption at 298 K (bottom) and desorption from 298 to 423 K (top) in ZIF-65(Zn)-II.

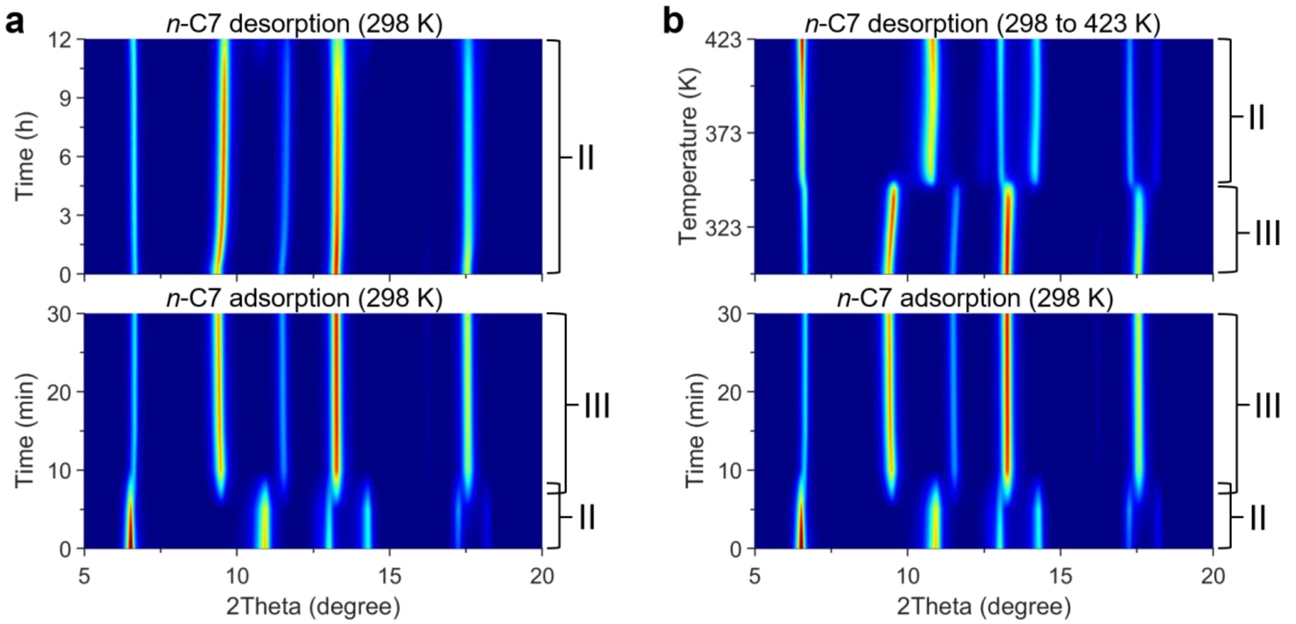

Supplementary Fig. 32 In situ PXRD for *n*-C7 vapor adsorption-desorption in ZIF-65(Zn)-II. a In situ PXRD patterns for *n*-C7 adsorption (bottom) and desorption (top) at 298 K in ZIF-65(Zn)-II, **b** in situ PXRD patterns for *n*-C7 adsorption at 298 K (bottom) and desorption from 298 to 423 K (top) in ZIF-65(Zn)-II.

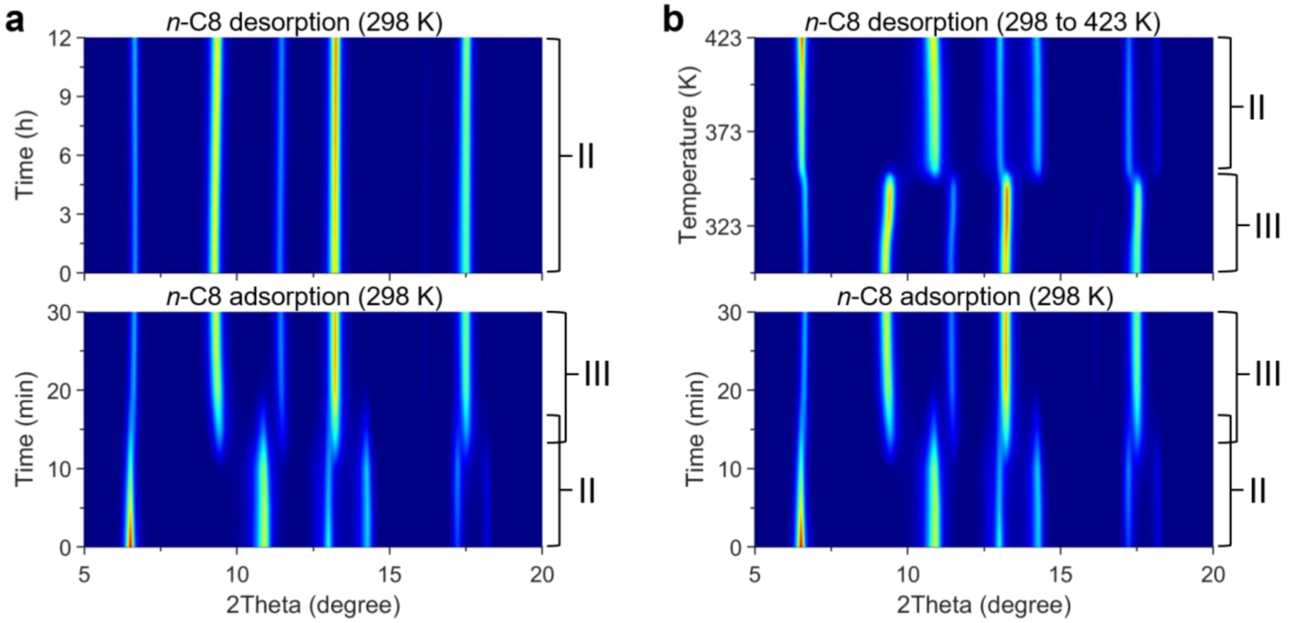

Supplementary Fig. 33 In situ PXRD for *n*-C8 vapor adsorption-desorption in ZIF-65(Zn)-II. a In situ PXRD patterns for *n*-C8 adsorption (bottom) and desorption (top) at 298 K in ZIF-65(Zn)-II, **b** in situ PXRD patterns for *n*-C8 adsorption at 298 K (bottom) and desorption from 298 to 423 K (top) in ZIF-65(Zn)-II.

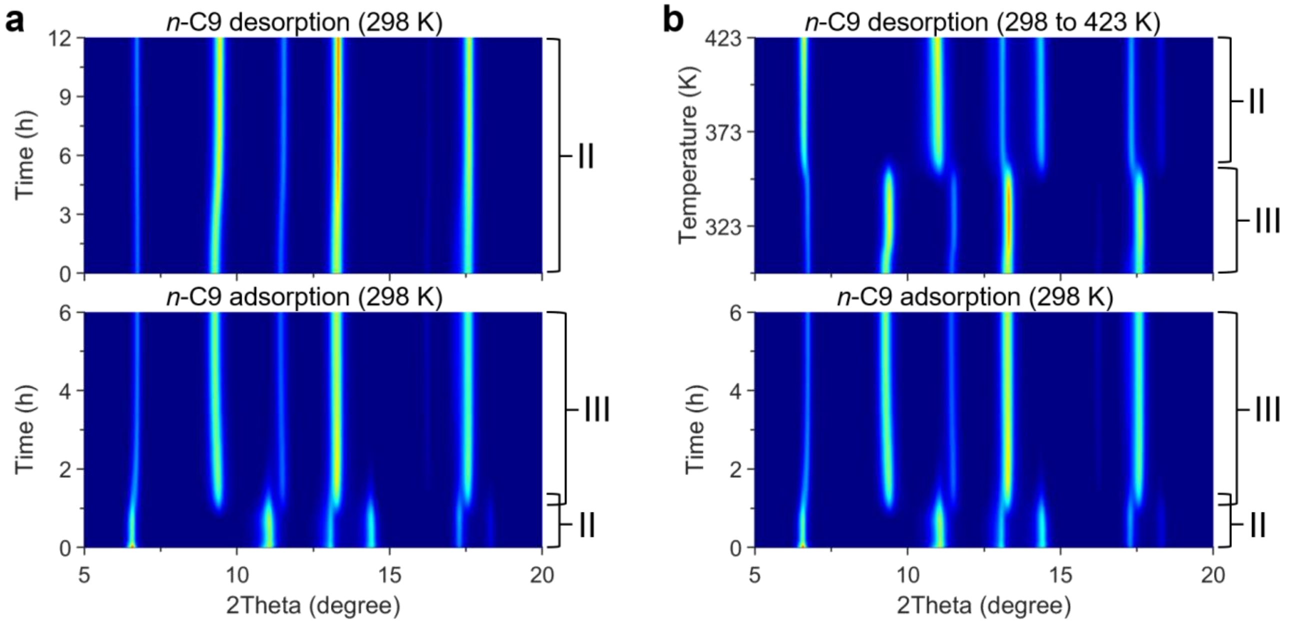

Supplementary Fig. 34 In situ PXRD for *n*-C9 vapor adsorption-desorption in ZIF-65(Zn)-II. a In situ PXRD patterns for *n*-C9 adsorption (bottom) and desorption (top) at 298 K in ZIF-65(Zn)-II, **b** in situ PXRD patterns for *n*-C9 adsorption at 298 K (bottom) and desorption from 298 to 423 K (top) in ZIF-65(Zn)-II.

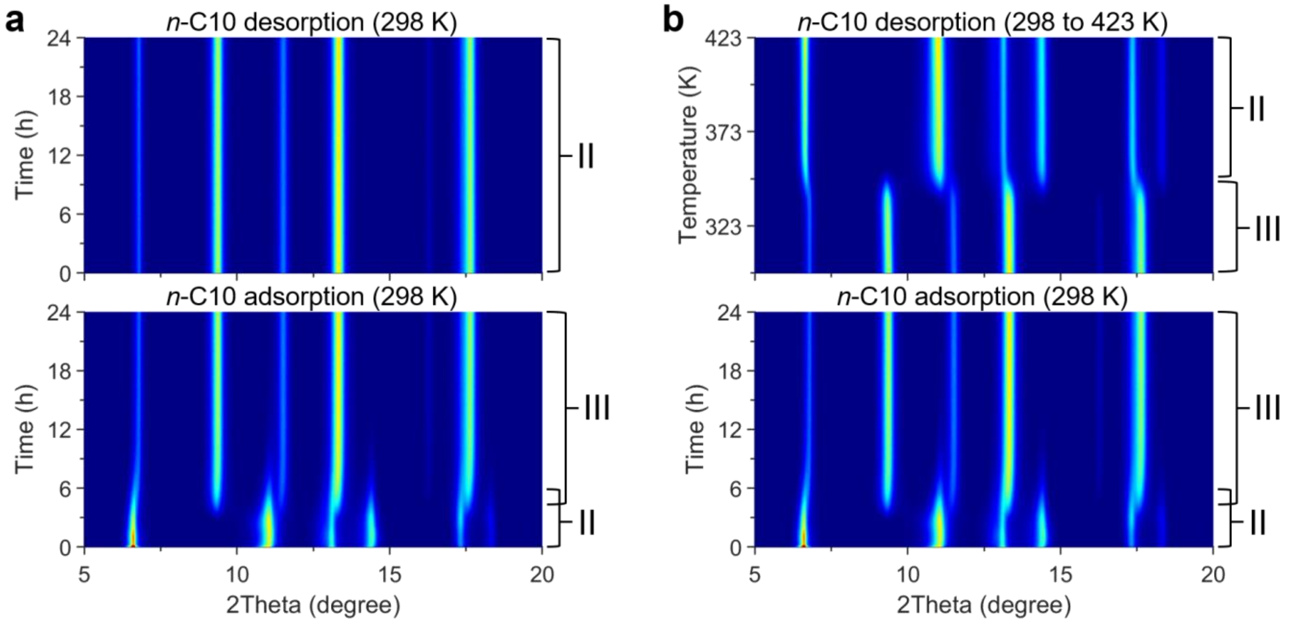

Supplementary Fig. 35 In situ PXRD for *n*-C10 vapor adsorption-desorption in ZIF-65(Zn)-II. a In situ PXRD patterns for *n*-C10 adsorption (bottom) and desorption (top) at 298 K in ZIF-65(Zn)-II, **b** in situ PXRD patterns for *n*-C10 adsorption at 298 K (bottom) and desorption from 298 to 423 K (top) in ZIF-65(Zn)-II.

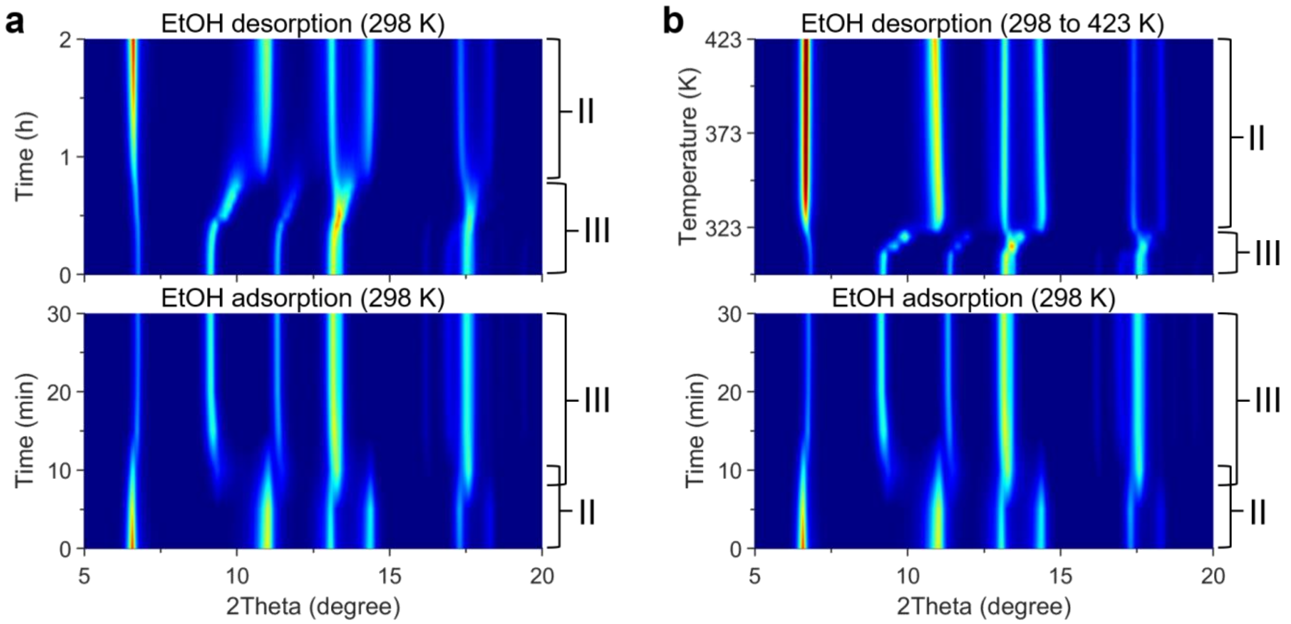

Supplementary Fig. 36 In situ PXRD for EtOH vapor adsorption-desorption in ZIF-65(Zn)-II. a In situ PXRD patterns for EtOH adsorption (bottom) and desorption (top) at 298 K in ZIF-65(Zn)-II, **b** in situ PXRD patterns for EtOH adsorption at 298 K (bottom) and desorption from 298 to 423 K (top) in ZIF-65(Zn)-II.

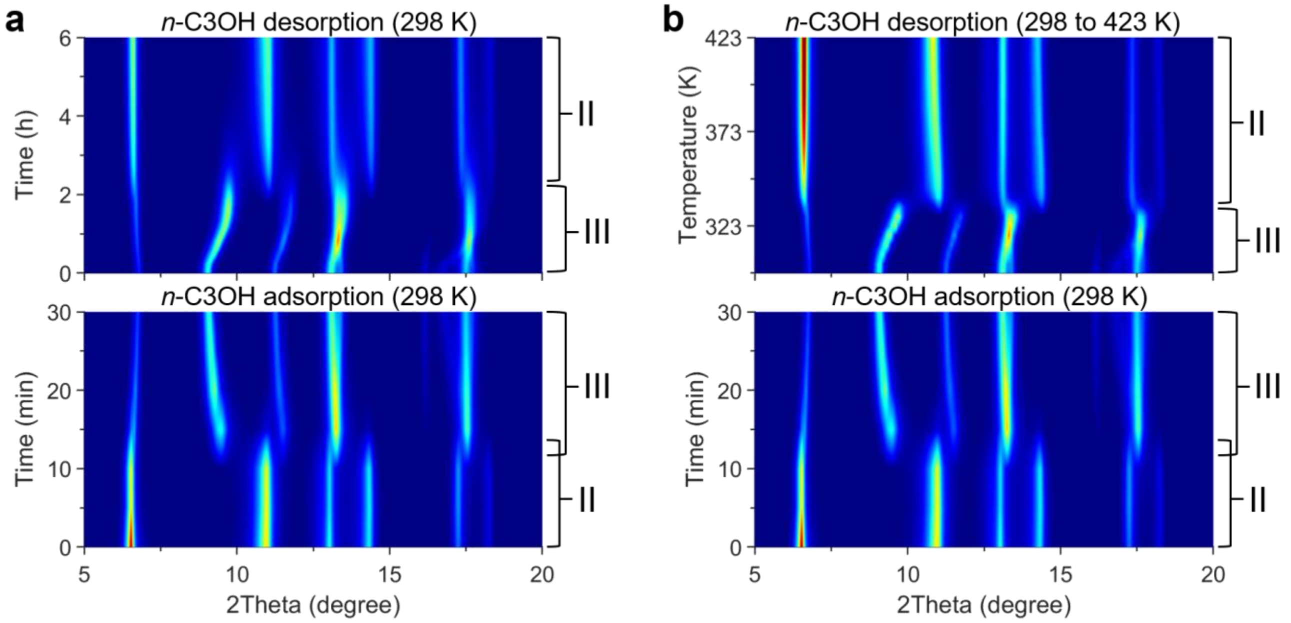

Supplementary Fig. 37 In situ PXRD for *n*-C3OH vapor adsorption-desorption in ZIF-65(Zn)-II. a In situ PXRD patterns for *n*-C3OH adsorption (bottom) and desorption (top) at 298 K in ZIF-65(Zn)-II, **b** in situ PXRD patterns for *n*-C3OH adsorption at 298 K (bottom) and desorption from 298 to 423 K (top) in ZIF-65(Zn)-II.

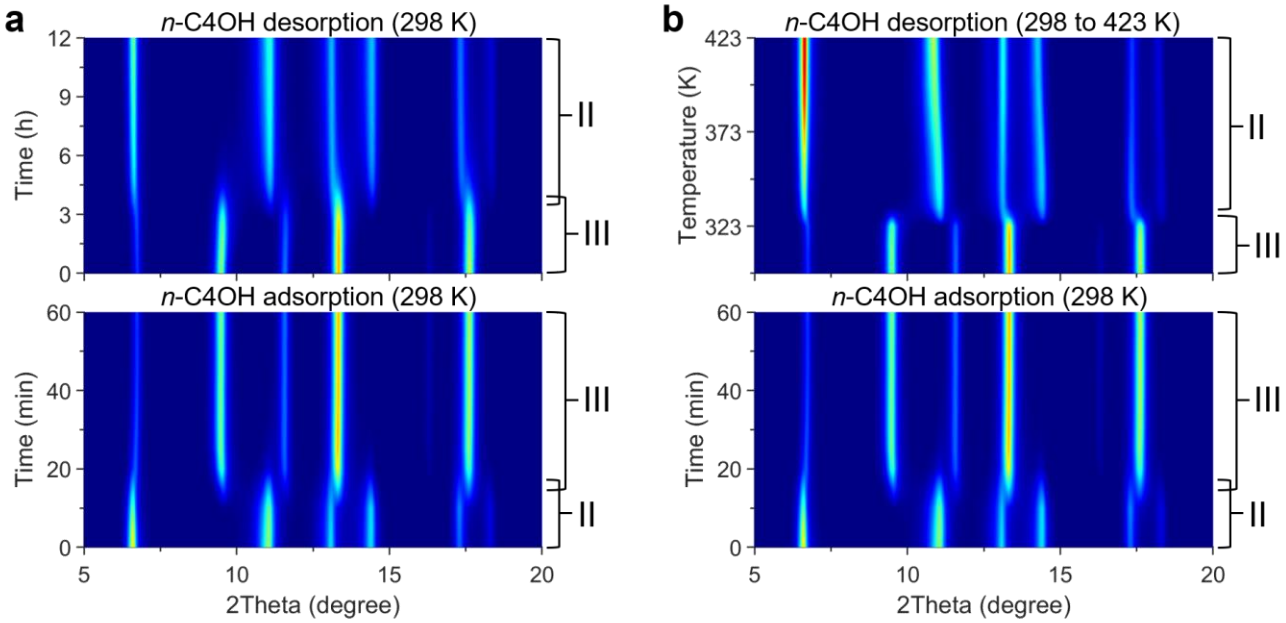

Supplementary Fig. 38 In situ PXRD for *n*-C4OH vapor adsorption-desorption in ZIF-65(Zn)-II. a In situ PXRD patterns for *n*-C4OH adsorption (bottom) and desorption (top) at 298 K in ZIF-65(Zn)-II, **b** in situ PXRD patterns for *n*-C4OH adsorption at 298 K (bottom) and desorption from 298 to 423 K (top) in ZIF-65(Zn)-II.

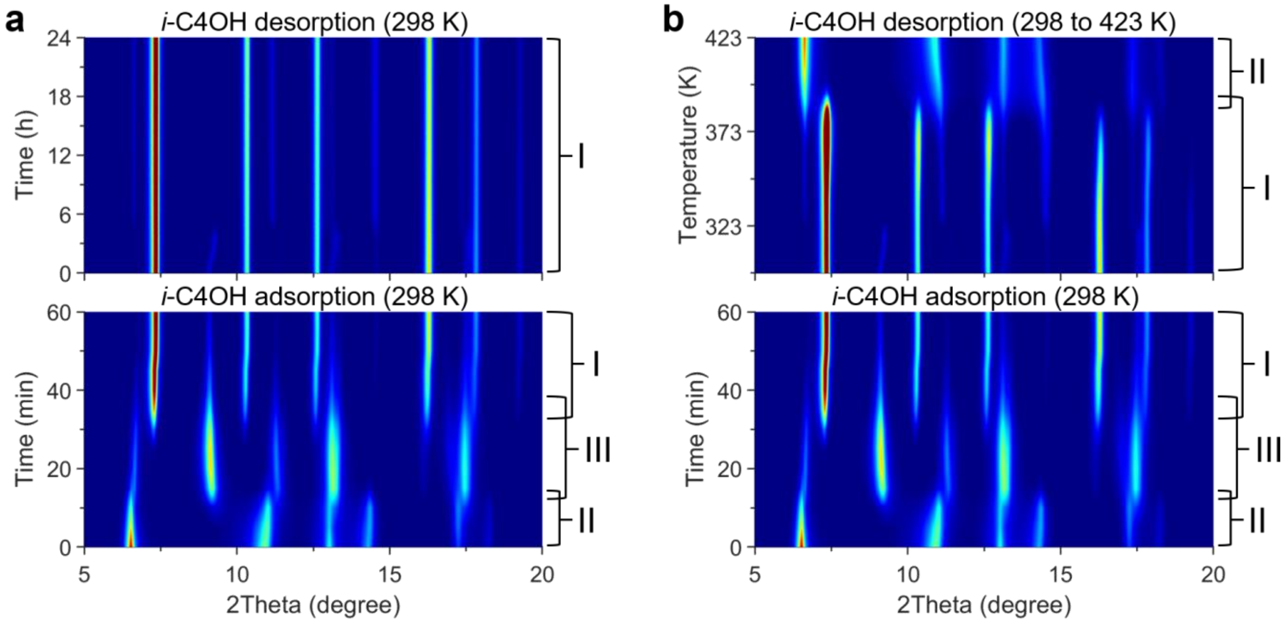

Supplementary Fig. 39 In situ PXRD for *i*-C4OH vapor adsorption-desorption in ZIF-65(Zn)-II. a In situ PXRD patterns for *i*-C4OH adsorption (bottom) and desorption (top) at 298 K in ZIF-65(Zn)-II, **b** in situ PXRD patterns for *i*-C4OH adsorption at 298 K (bottom) and desorption from 298 to 423 K (top) in ZIF-65(Zn)-II.

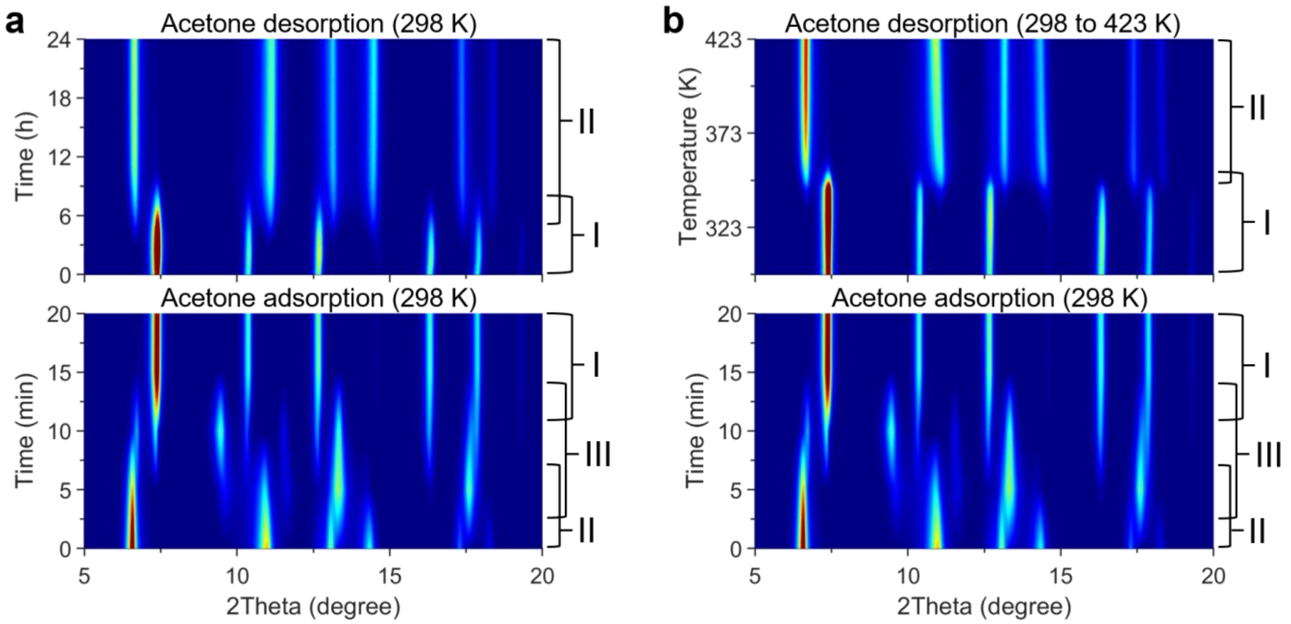

Supplementary Fig. 40 In situ PXRD for acetone vapor adsorption-desorption in ZIF-65(Zn)-II. a In situ PXRD patterns for acetone adsorption (bottom) and desorption (top) at 298 K in ZIF-65(Zn)-II, **b** in situ PXRD patterns for acetone adsorption at 298 K (bottom) and desorption from 298 to 423 K (top) in ZIF-65(Zn)-II.

S2.5 The dynamic structural transition of ZIF-65(Zn)

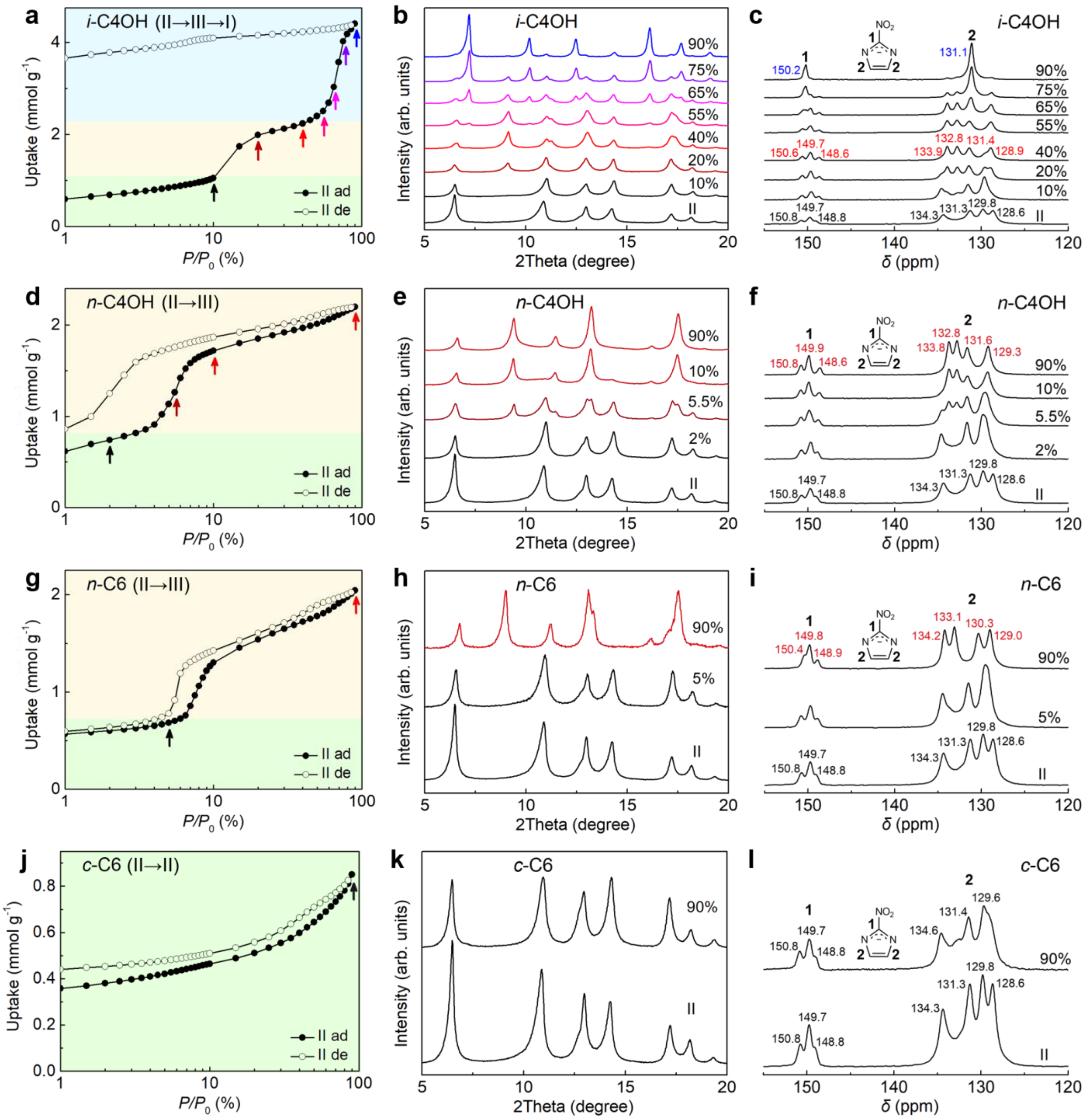

Supplementary Fig. 41 The dynamic structural transition for *c*-C6 (II→II), *n*-C6 (II→III), *n*-C4OH (II→III), and *i*-C4OH (II→III→I) adsorption in ZIF-65(Zn)-II at 298 K. The adsorption (solid) and desorption (empty) logarithmic isotherms (**a, d, g, j**). The PXRD patterns upon increasing the relative pressure (**b, e, h, k**). The ^{13}C NMR spectra upon increasing the relative pressure (**c, f, i, l**).

The *c*-C6 adsorption (0.9 mmol g^{-1} at P/P_0 = 90%) mainly occurs in the pore volume of II (Supplementary Fig. 41j). Meanwhile, the PXRD and ^{13}C NMR spectra of II are similar before and after adsorption, which indicates no structural transition can be observed (Supplementary Fig. 41k, 41l). Noted that the first reflection intensity significantly decreases in the PXRD pattern and the two sharp peaks (δ = 129.8/128.6 ppm) begin to merge into a large broad peak in the ^{13}C NMR spectrum for II·(*c*-C6) (P/P_0 = 90%) due to the adsorption of *c*-C6.

For *n*-C6 adsorption, ZIF-65(Zn)-II exhibits a pre-step and step-shaped adsorption (Supplementary Fig. 41g). ZIF-65(Zn)-II shows an initial *n*-C6 uptake below 5% (P/P_0) and remains II from PXRD observation (Supplementary Fig. 41h), in which the adsorption mainly occurs in the pore volume of II. The step-shaped adsorption starts at P/P_0 = 5% (0.7 mmol g^{-1}) and ends at P/P_0 = 90% (2.0 mmol g^{-1}), which corresponds to the II→III structural transition from PXRD observation, and the adsorption mainly occurs in the pore volume of III. The ^{13}C NMR spectrum of II (no guest) shows three peaks (δ = 150.8/149.7/148.8 ppm) representing C#1 of the nIm linker and four peaks (δ = 134.3/131.3/129.8/128.6 ppm) representing C#2 of in nIm linker (Supplementary Fig. 41i), which is consistent with the three conformations of the nIm linker according to the structure of II (Supplementary Fig. 13 and Supplementary Table 4). From the ^{13}C NMR spectrum of III·(*n*-C6) (P/P_0 = 90%), in terms of C#1 of nIm linker, three peaks are similar to those in II; in terms of C#2 of nIm linker, three peaks (δ = 134.2/130.3/129.0 ppm) are similar to those in II, but another one peak (δ = 133.1 ppm) appears with the disappearance of another one peaks (δ = 131.3 ppm) in II, which suggest that some difference must exist between the structures of II and III. The most important difference is found that the N–Zn–N angle decreases from 155.4° (II) to 124.0°(III·(*n*-C6)) (Supplementary Fig. 12) associated with the displacement of nIm, which leads to structural transition. [Note: the ^{13}C NMR spectrum of III·(*n*-C6) is similar to that of III·(*n*-C10) in Supplementary Fig. 42, thus the structure of III·(*n*-C6) should be basically the same as that of III·(*n*-C10). The structure of III·(*n*-C6) has not been solved due to the volatile nature of the *n*-C6 molecule, but can refer to that of III·(*n*-C10)].

The isotherm trend of *n*-C4OH adsorption in ZIF-65(Zn)-II is similar to that of *n*-C6 adsorption (Supplementary Fig. 41d). ZIF-65(Zn)-II after *n*-C4OH adsorption at P/P_0 = 90% also occurs the distinct II→III structural transitions (Supplementary Fig. 41e). The ^{13}C NMR spectrum of III·(*n*-C4OH) (P/P_0 = 90%) is different from that of III·(*n*-C6) (P/P_0 = 90%) (Supplementary Fig. 41f, 41i). For the ^{13}C NMR spectrum of III·(*n*-C4OH), in terms of C#1 of nIm linker, three peaks are also similar to those in II; in terms of C#2 of nIm linker, two peaks (δ = 131.6/129.3 ppm) are similar to those in II, but another two peaks (δ = 133.8/132.8 ppm) appear with the disappearance of another two peaks (δ = 134.3/129.8 ppm) in II. The most important difference is found that the N–Zn–N angle decreases from 155.4/138.8° (II) to 131.1/96.1° (III·(*n*-C4OH)) (Supplementary Fig. 12) associated with the displacement of nIm, which leads to the structural transition.

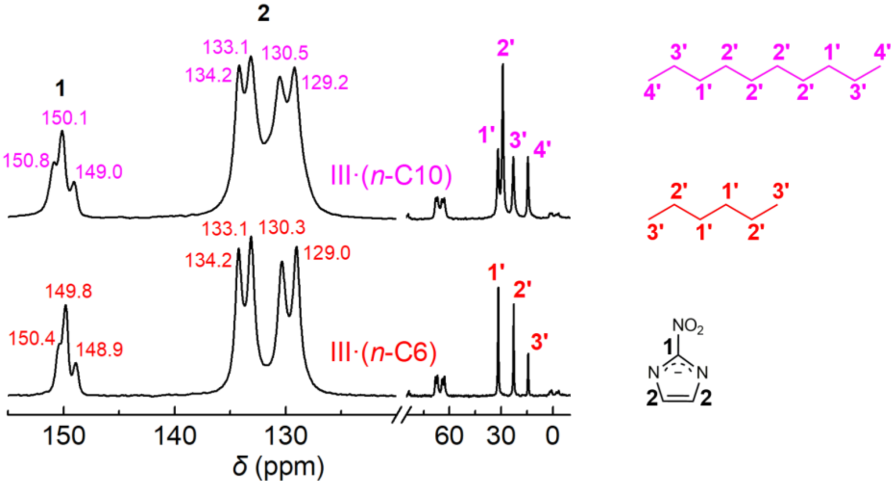

Supplementary Fig. 42 The ^{13}C NMR spectra for III·(*n*-C6) and III·(*n*-C10). [Note: III·(*n*-C6) and III·(*n*-C10) are *n*-C6 and *n*-C10 adsorption in ZIF-65(Zn)-II at P/P_0 = 90%, respectively.]

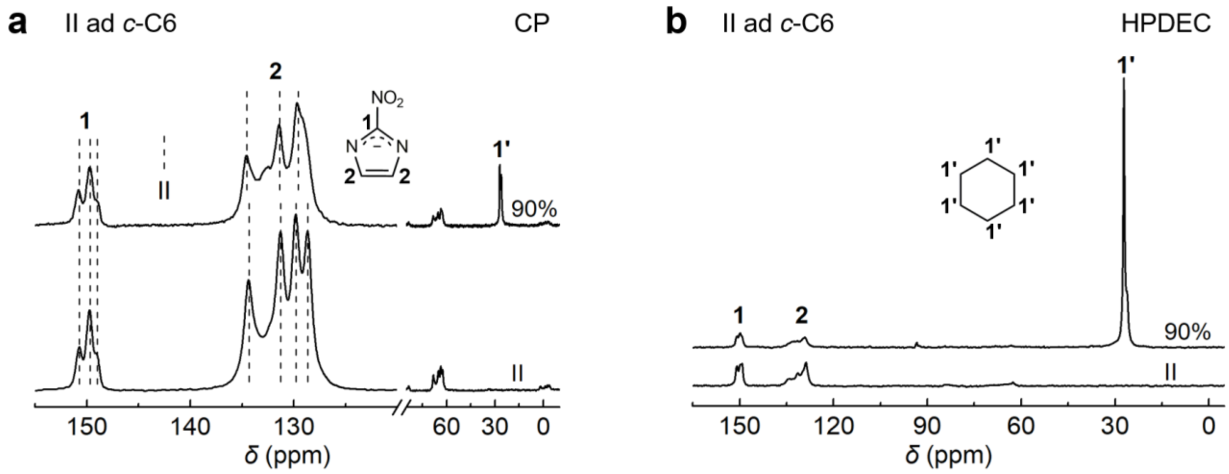

Supplementary Fig. 43 Solid-state ^{13}C NMR spectra for *c*-C6 adsorption in ZIF-65(Zn)-II upon increasing the relative pressure. a ^{13}C (CP) NMR, and **b** ^{13}C (HPDEC) NMR spectra.

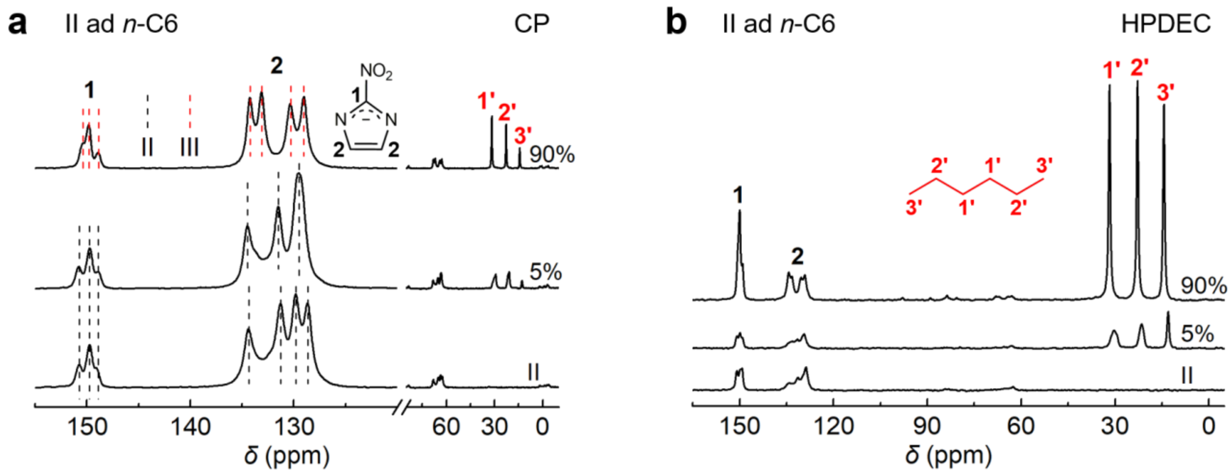

Supplementary Fig. 44 Solid-state ^{13}C NMR spectra for *n*-C6 adsorption in ZIF-65(Zn)-II upon increasing the relative pressure. a ^{13}C (CP) NMR, and **b** ^{13}C (HPDEC) NMR spectra.

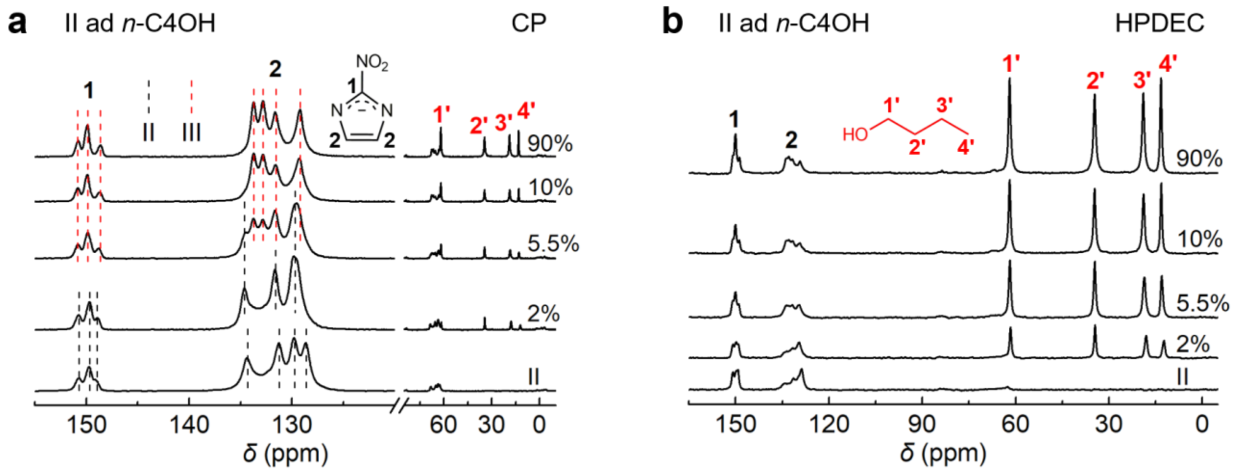

Supplementary Fig. 45 Solid-state ^{13}C NMR spectra for *n*-C4OH adsorption in ZIF-65(Zn)-II upon increasing the relative pressure. a ^{13}C (CP) NMR, and **b** ^{13}C (HPDEC) NMR spectra.

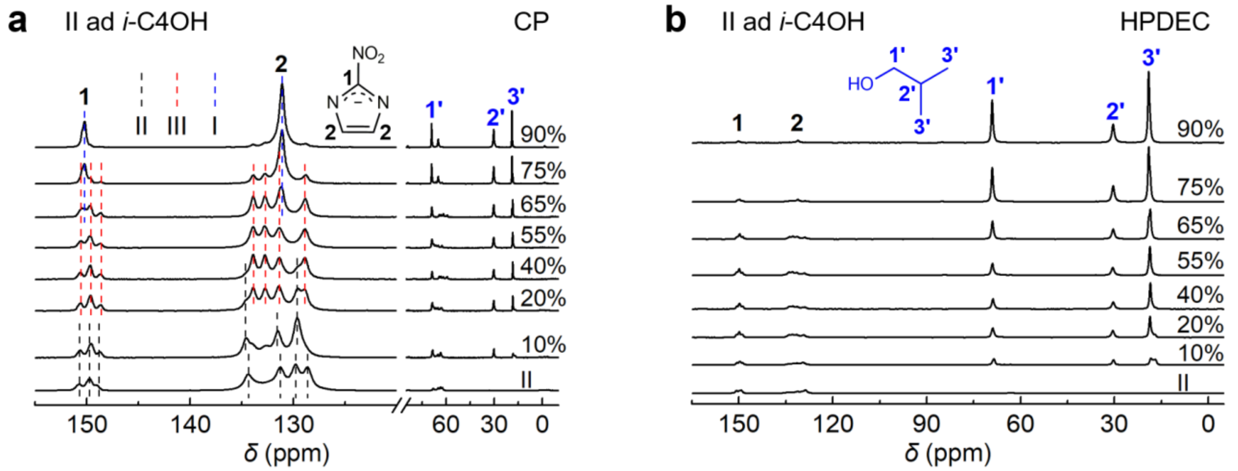

Supplementary Fig. 46 Solid-state ^{13}C NMR spectra for *i*-C4OH adsorption in ZIF-65(Zn)-II upon increasing the relative pressure. a ^{13}C (CP) NMR, and **b** ^{13}C (HPDEC) NMR spectra.

2.6 Understanding the flexibility of ZIF-65(Zn)

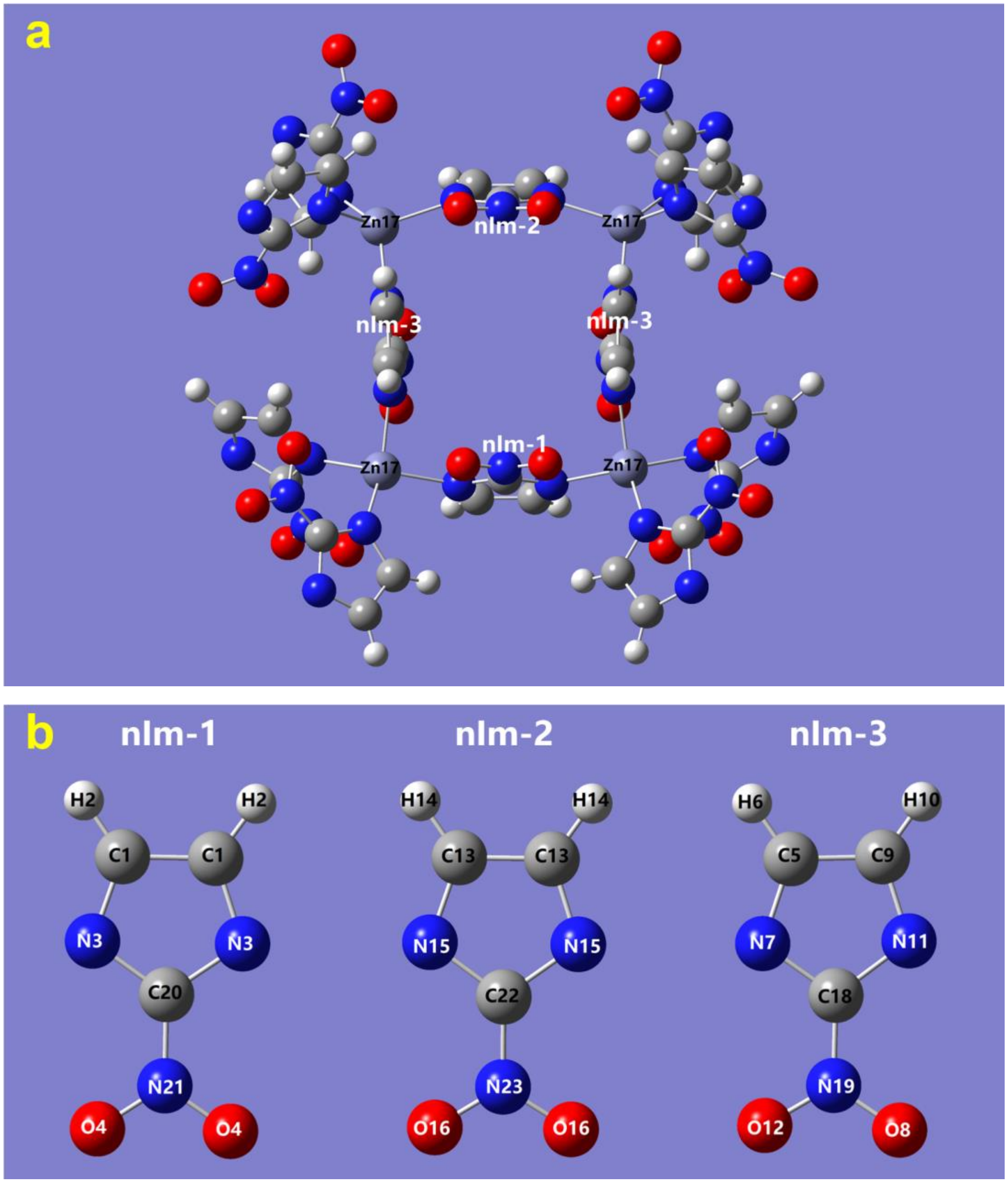

Supplementary Fig. 47 Calculation of partial charges on ZIF-65(Zn)-II. a Details of clusters used for deriving partial charges. **b** Three nIm conformations and their atomic names.

Supplementary Table 8 Partial atomic charges for **ZIF-65(Zn)-II**.

**Atom**	**Zn17**	**C1**	**H2**	**N3**	**C20**	**N21**	**O4**
**Charge(e)**	0.8790	-0.0525	0.1828	-0.2302	0.0555	0.7172	-0.4635
**Atom**		**C13**	**H14**	**N15**	**C22**	**N23**	**O16**
**Charge(e)**		0.0468	0.1109	-0.4817	0.3507	0.6864	-0.4832
**Atom**		**C5**	**H6**	**N7**	**C18**	**N19**	**O8**
**Charge(e)**		0.0223	0.1668	-0.4010	0.0755	0.8582	-0.4787
**Atom**		**C9**	**H10**	**N11**			**O12**
**Charge(e)**		-0.0297	0.1679	-0.2678			-0.5268

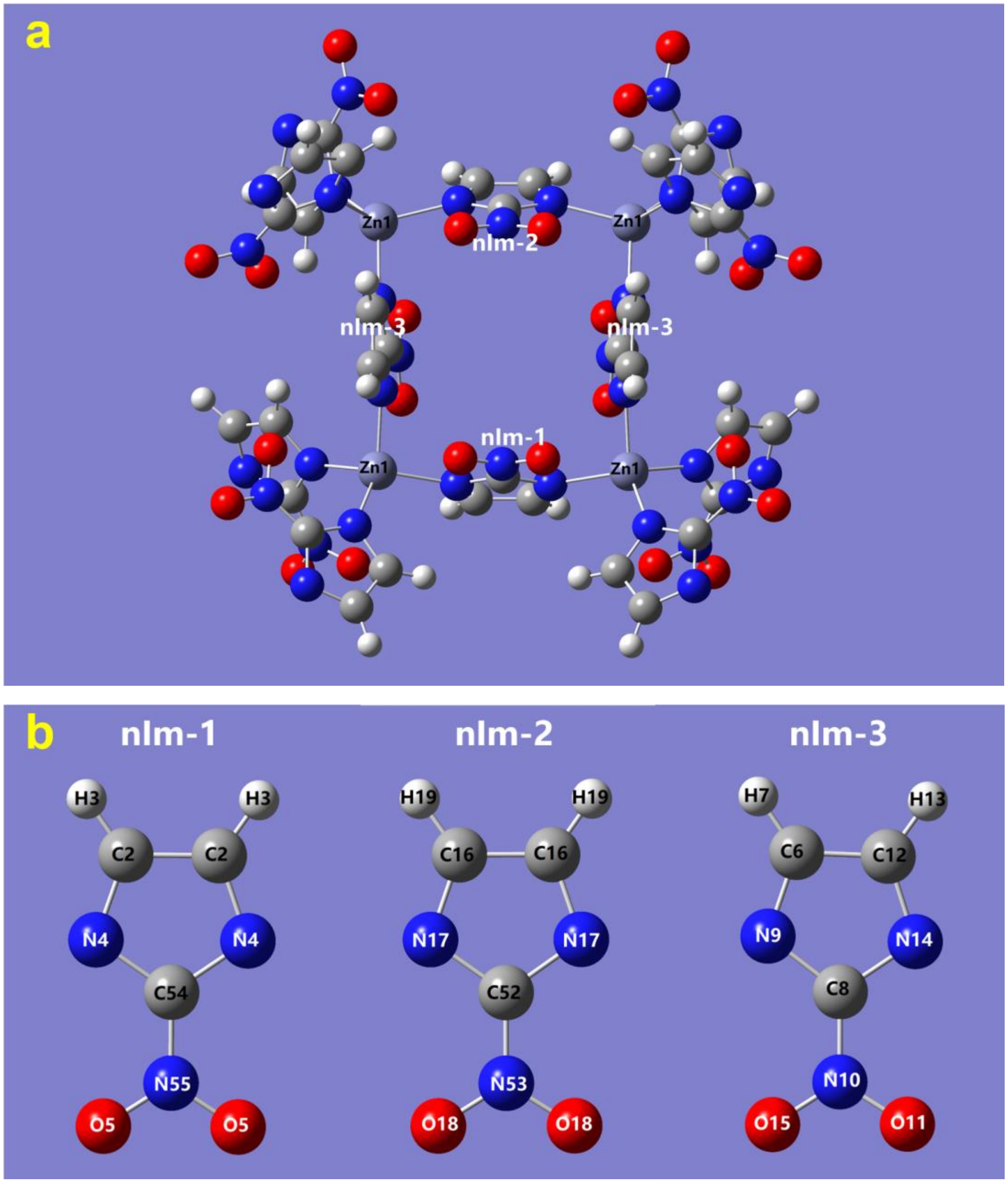

Supplementary Fig. 48 Calculation of partial charges on ZIF-65(Zn)-III·(*n*-C10). a Details of clusters used for deriving partial charges. **b** Three nIm conformations and their atomic names.

Supplementary Table 9 Partial atomic charges for **ZIF-65(Zn)-III·(*n*-C10)**.

**Atom**	**Zn1**	**C2**	**H3**	**N4**	**C54**	**N55**	**O5**
**Charge(e)**	0.8391	-0.0145	0.1621	-0.2476	0.0779	0.7113	-0.4734
**Atom**		**C16**	**H19**	**N17**	**C52**	**N53**	**O18**
**Charge(e)**		0.0640	0.1010	-0.4162	0.1953	0.7865	-0.5203
**Atom**		**C6**	**H7**	**N9**	**C8**	**N10**	**O11**
**Charge(e)**		0.0530	0.1355	-0.2598	-0.0067	0.8376	-0.5249
**Atom**		**C12**	**H13**	**N14**			**O15**
**Charge(e)**		-0.0269	0.1693	-0.2796			-0.4772

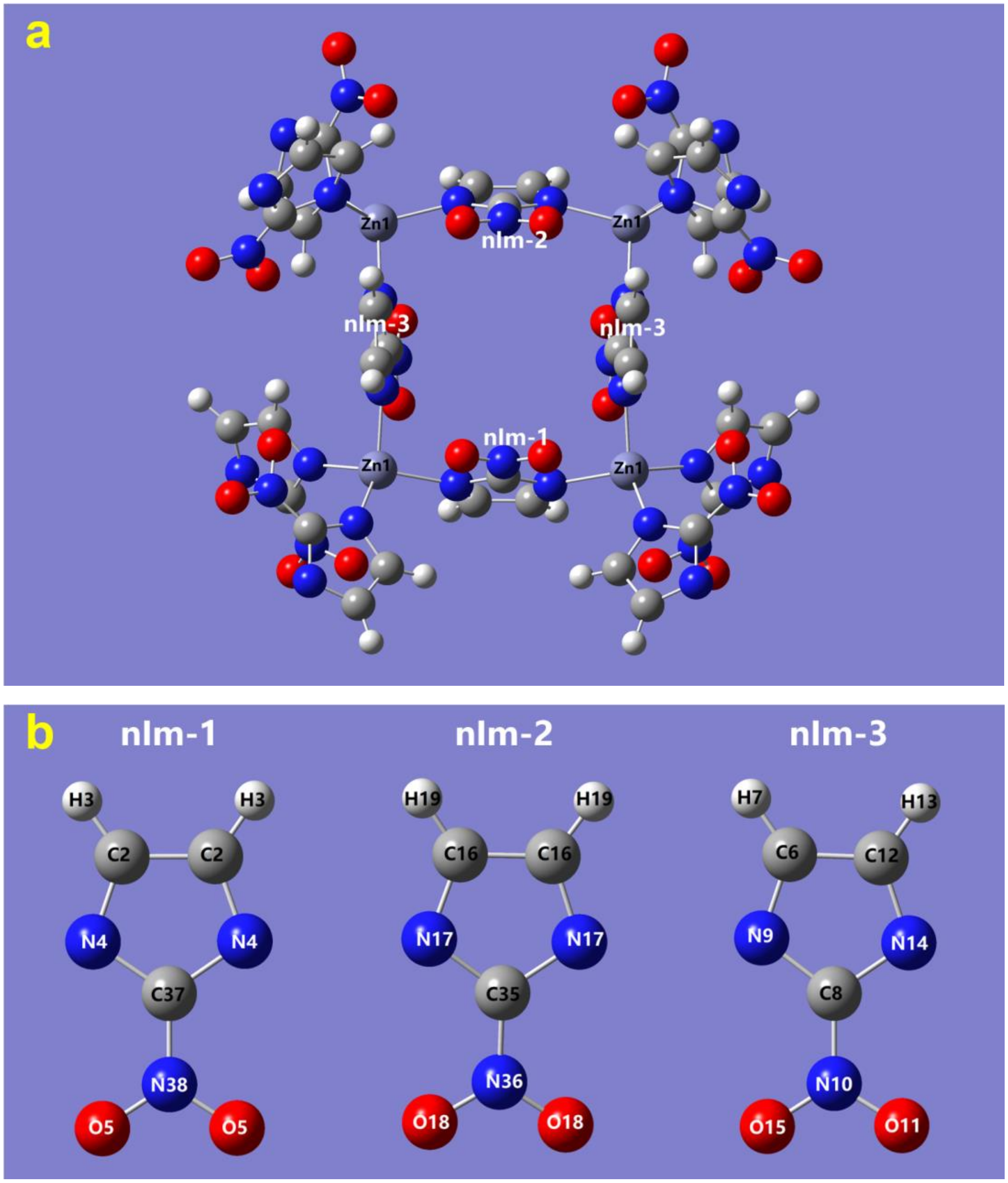

Supplementary Fig. 49 Calculation of partial charges on ZIF-65(Zn)-III·(*n*-C4OH). a Details of clusters used for deriving partial charges. **b** Three nIm conformations and their atomic names.

Supplementary Table 10 Partial atomic charges for **ZIF-65(Zn)-III·(*n*-C4OH)**.

**Atom**	**Zn1**	**C2**	**H3**	**N4**	**C37**	**N38**	**O5**
**Charge(e)**	0.8521	-0.0217	0.1686	-0.2368	0.0521	0.7480	-0.5207
**Atom**		**C16**	**H19**	**N17**	**C35**	**N36**	**O18**
**Charge(e)**		0.0595	0.1059	-0.4288	0.2388	0.7681	-0.5091
**Atom**		**C6**	**H7**	**N9**	**C8**	**N10**	**O11**
**Charge(e)**		0.0414	0.1424	-0.2662	0.0185	0.8425	-0.5207
**Atom**		**C12**	**H13**	**N14**			**O15**
**Charge(e)**		-0.0236	0.1737	-0.3044			-0.4761

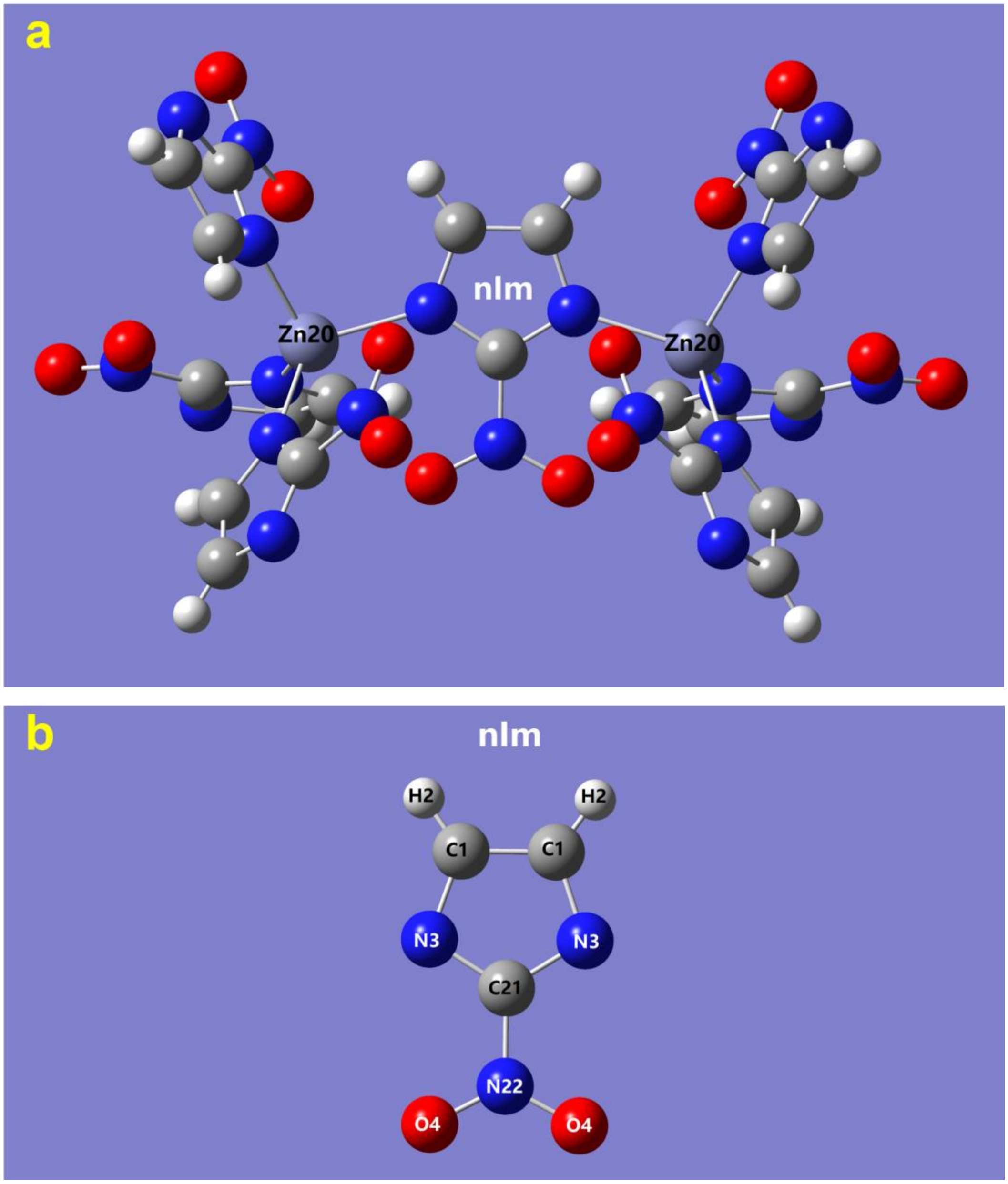

Supplementary Fig. 50 Calculation of partial charges on ZIF-65(Zn)-I_*I*-43*m*. a Details of clusters used for deriving partial charges. **b** One unique nIm conformation and its atomic names.

Supplementary Table 11 Partial atomic charges for **ZIF-65(Zn)-I_*I*-43m**.

**Atom**	**Zn20**	**C1**	**H2**	**N3**	**C21**	**N22**	**O4**
**Charge(e)**	0.7920	-0.3000	0.1720	-0.1200	0.4500	0.7100	-0.5300

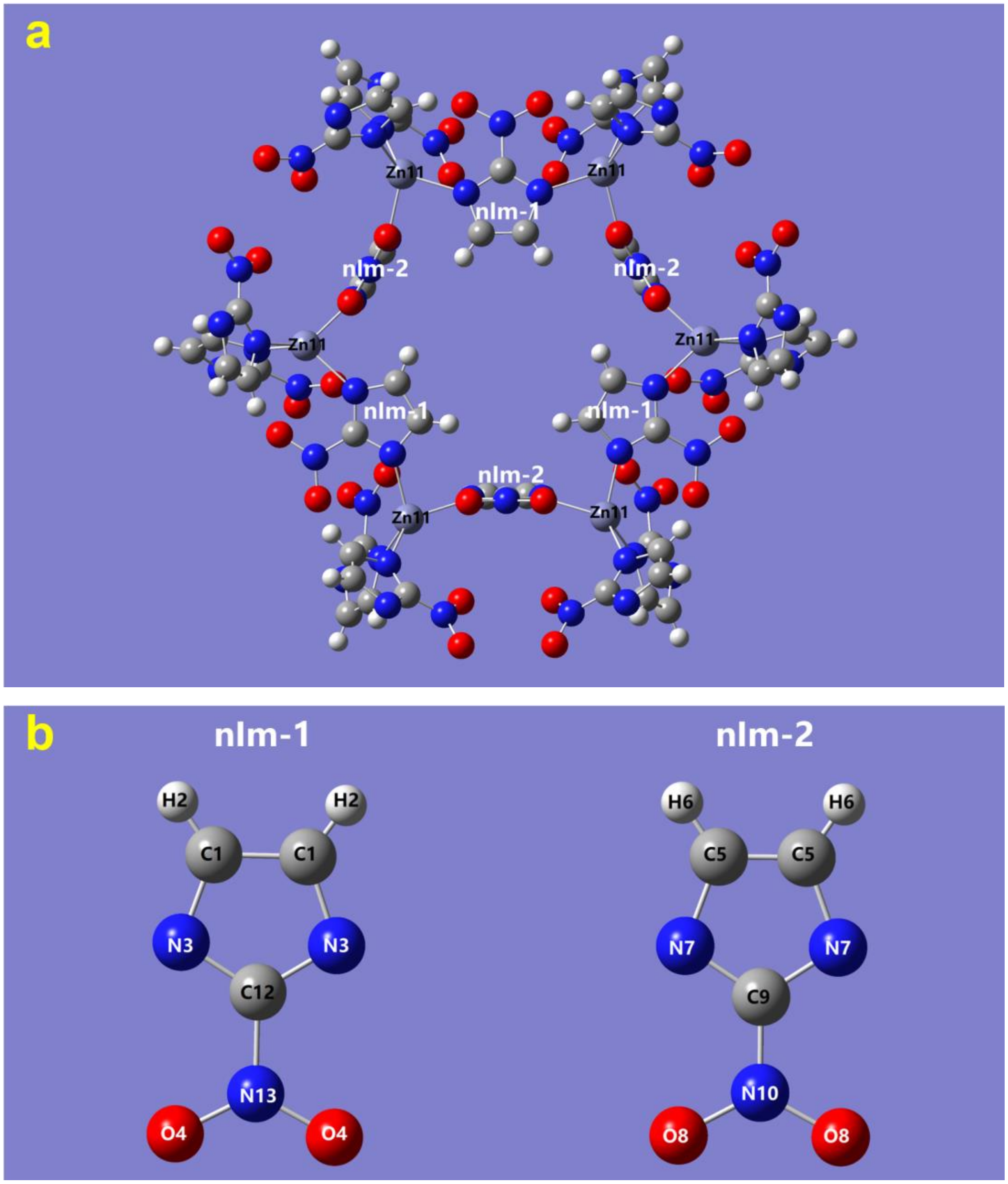

Supplementary Fig. 51 Calculation of partial charges on ZIF-65(Zn)-I_*P*-43*m*. a Details of clusters used for deriving partial charges. **b** Two nIm conformations and their atomic names.

Supplementary Table 12 Partial atomic charges for **ZIF-65(Zn)-I_*P*-43*m***.

**Atom**	**Zn11**	**C1**	**H2**	**N3**	**C12**	**N13**	**O4**
**Charge(e)**	0.8134	-0.0380	0.1311	-0.3229	0.3402	0.5066	-0.3859
**Atom**		**C5**	**H6**	**N7**	**C9**	**N10**	**O8**
**Charge(e)**		-0.0211	0.1036	-0.2904	0.2572	0.6388	-0.4545

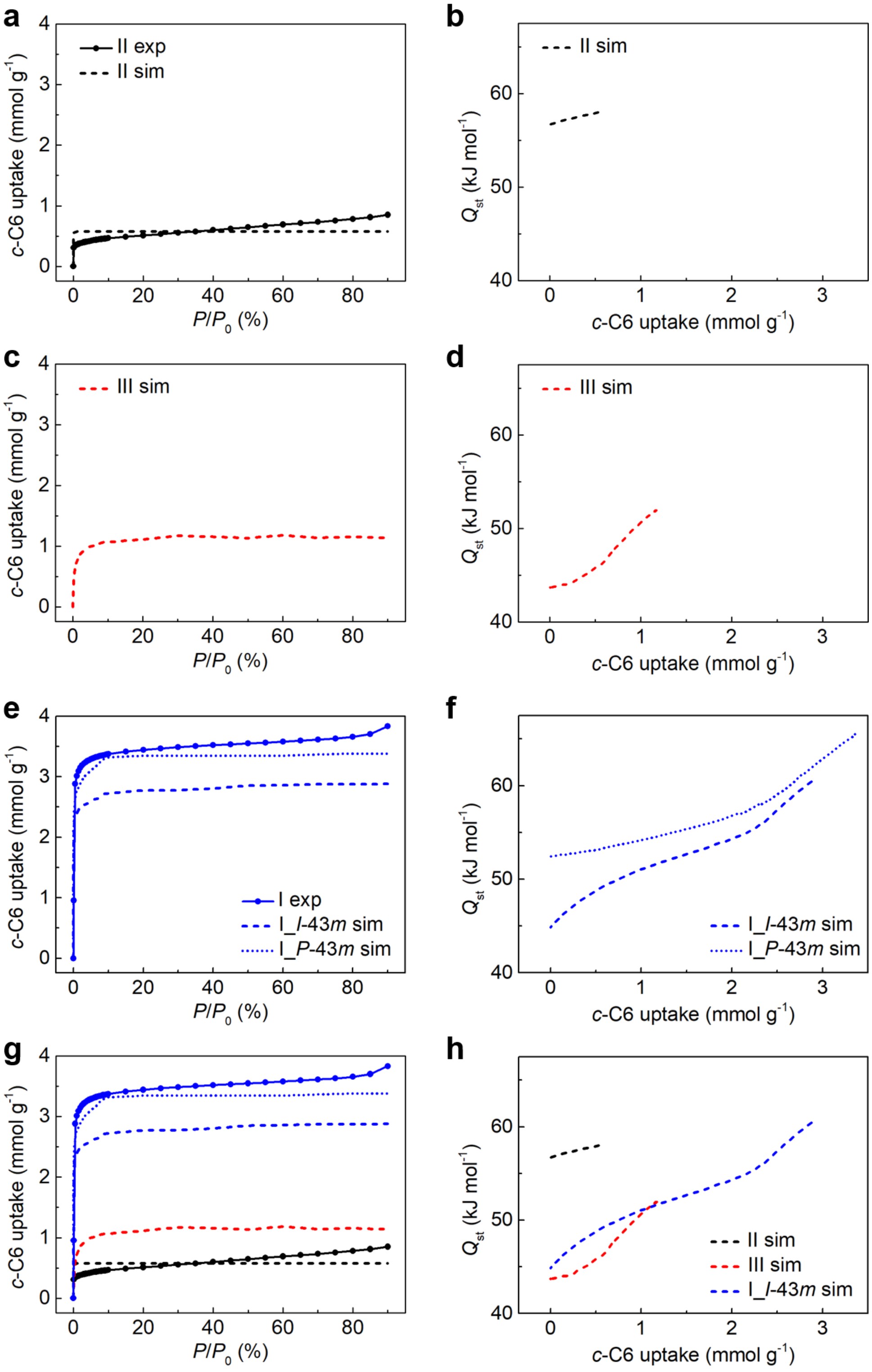

Supplementary Fig. 52 Comparison of the simulated and experimental *c*-C6 adsorption isotherms for ZIF-65(Zn) and corresponding simulated guest adsorption heat at 298 K. **a** Comparison of the simulated and the experimental isotherms of II. **c** Simulated isotherm of III. **e** Comparison of the simulated isotherms of I_*I*-43*m* and I_*P*-43*m* with the experimental isotherm of I. **g** The summary of all simulated and experimental isotherms. The simulated adsorption heats in **b** II, **d** III, **f** I_*I*-43*m* and I_*P*-43*m*, and **h** II, III, and I_*I*-43*m*. (Experimental data: solid line; simulated result: dashed line.)

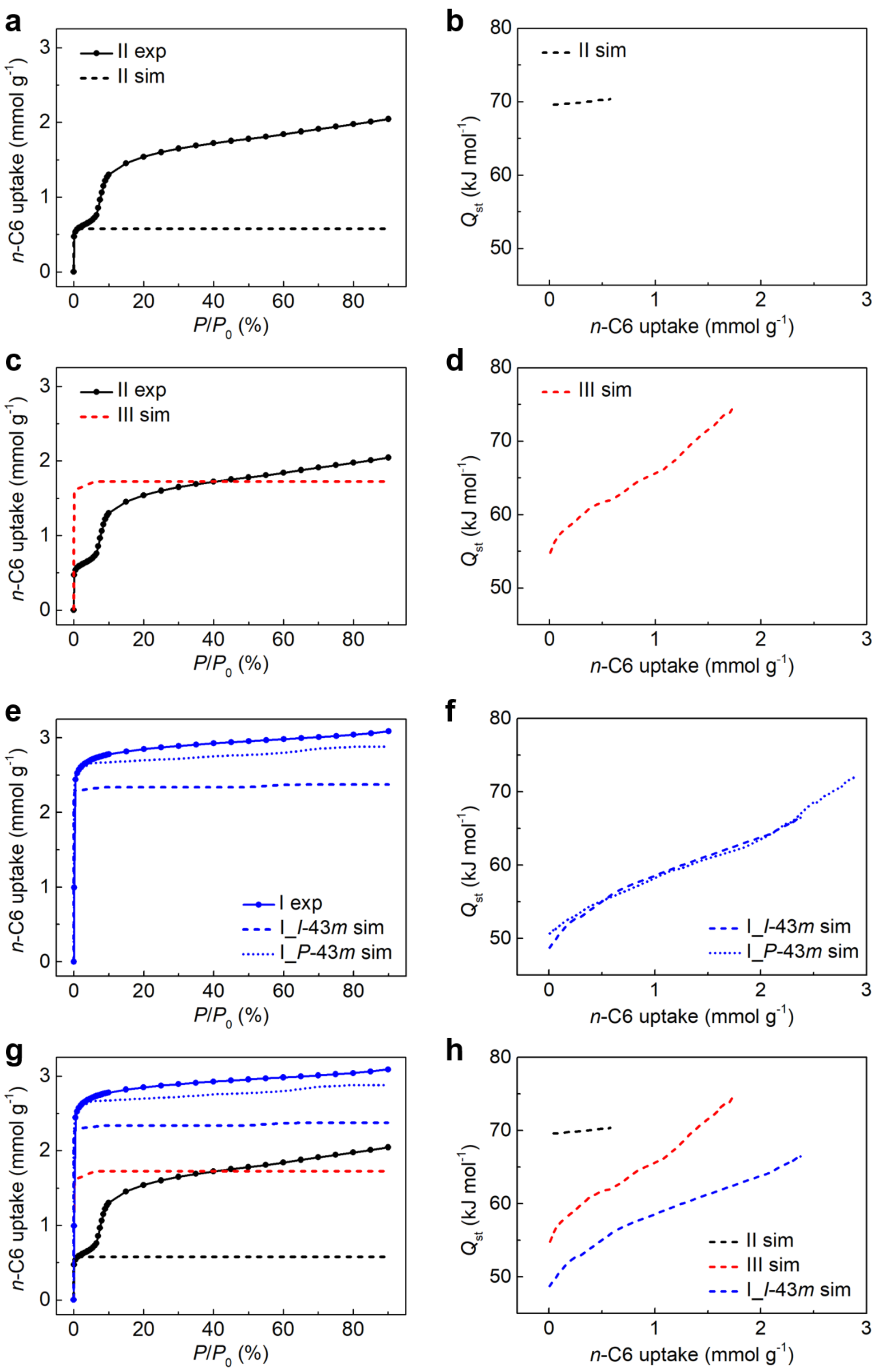

Supplementary Fig. 53 Comparison of the simulated and experimental *n*-C6 adsorption isotherms for ZIF-65(Zn) and corresponding simulated guest adsorption heat at 298 K. Comparison of the simulated isotherms of **a** II and **c** III with the first and second plateau on the experimental isotherm of II, respectively. Comparison of the simulated adsorption isotherms of **e** I_*I*-43*m* and I_*P*-43*m* with the experimental isotherm of I. **g** The summary of all simulated and experimental isotherms. The simulated adsorption heats in **b** II, **d** III, **f** I_*I*-43*m* and I_*P*-43*m*, and **h** II, III, and I_*I*-43*m*. (Experimental data: solid line; simulated result: dashed line.)

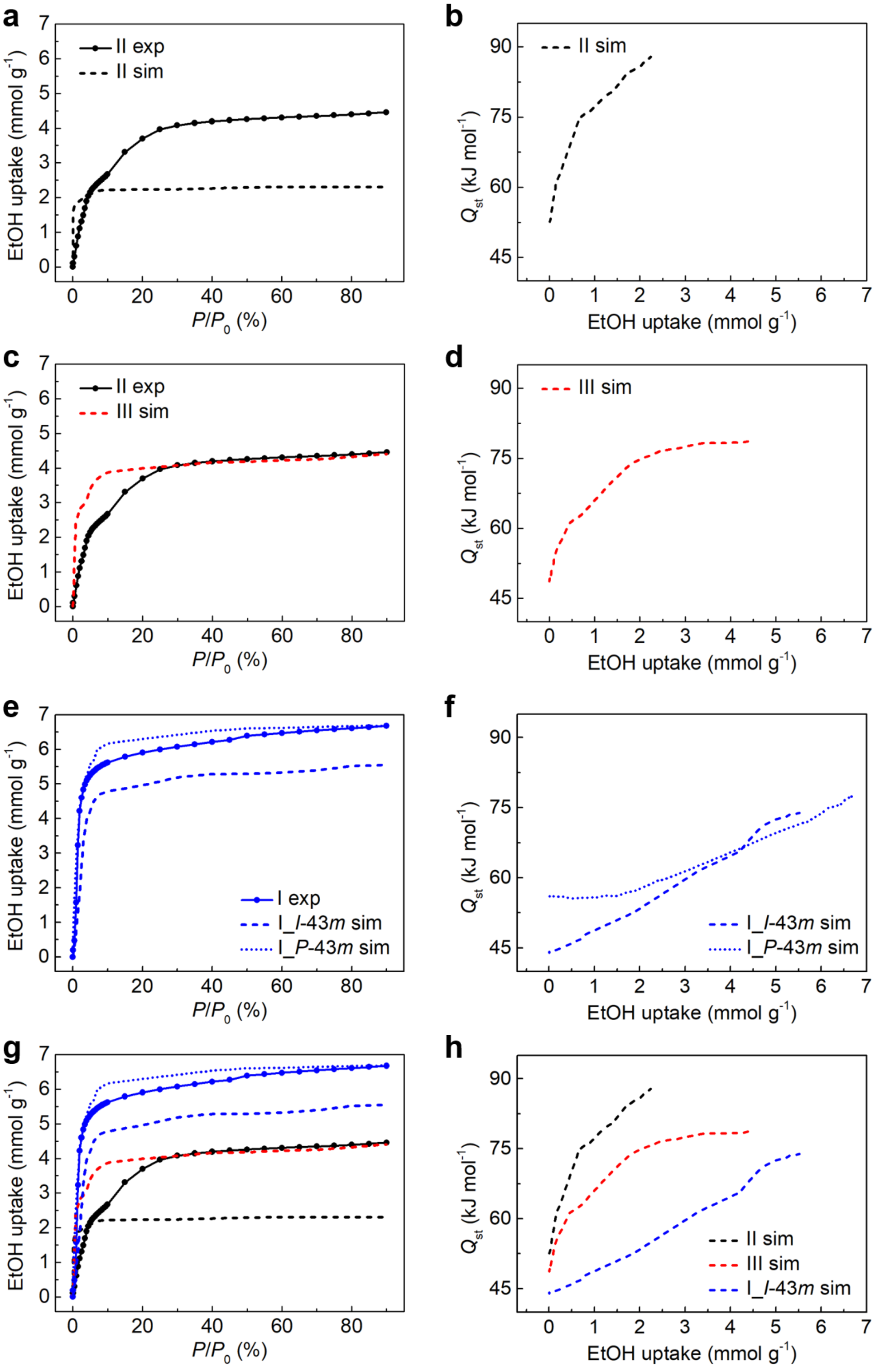

Supplementary Fig. 54 Comparison of the simulated and experimental EtOH adsorption isotherms for ZIF-65(Zn) and corresponding simulated guest adsorption heat at 298 K. Comparison of the simulated isotherms of **a** II and **c** III with the first and second plateau on the experimental isotherm of II, respectively. Comparison of the simulated isotherms of **e** I_*I*-43*m* and I_*P*-43*m* with the experimental isotherm of I. **g** The summary of all simulated and experimental isotherms. The simulated adsorption heats in **b** II, **d** III, **f** I_*I*-43*m* and I_*P*-43*m*, and **h** II, III, and I_*I*-43*m*. (Experimental data: solid line; simulated result: dashed line.)

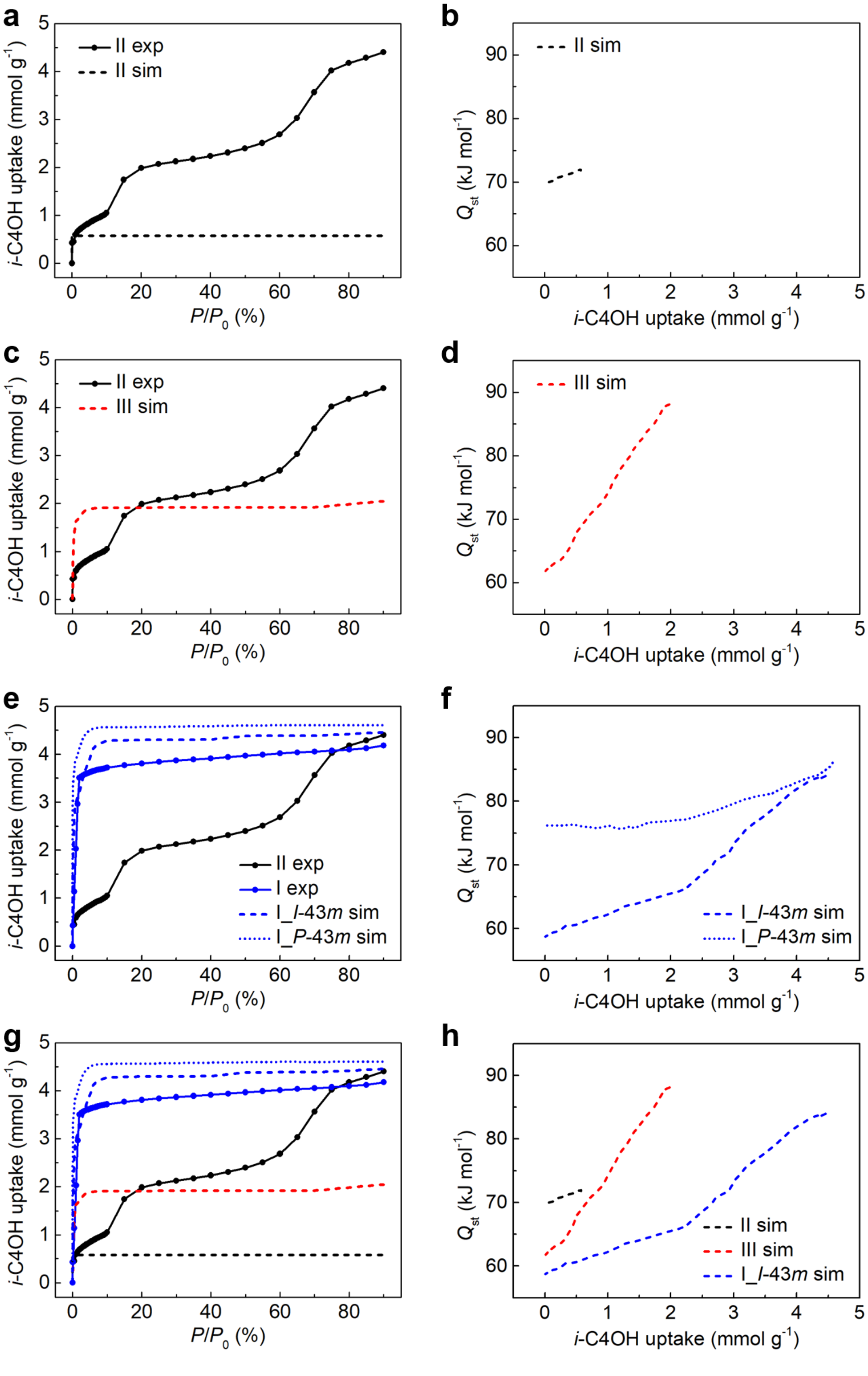

Supplementary Fig. 55 Comparison of the simulated and experimental *i*-C4OH adsorption isotherms for ZIF-65(Zn) and corresponding simulated guest adsorption heat at 298 K. Comparison of the simulated isotherms of **a** II and **c** III with the first and second plateau on the experimental isotherm of II, respectively. Comparison of the simulated isotherms of **e** I_*I*-43*m* and I_*P*-43*m* with the third plateau on the experimental isotherm of II and the experimental isotherm of I. **g** The summary of all simulated and experimental isotherms. The simulated adsorption heats in **b** II, **d** III, **f** I_*I*-43*m* and I_*P*-43*m*, and **h** II, III, and I_*I*-43*m*. (Experimental data: solid line; simulated result: dashed line.)

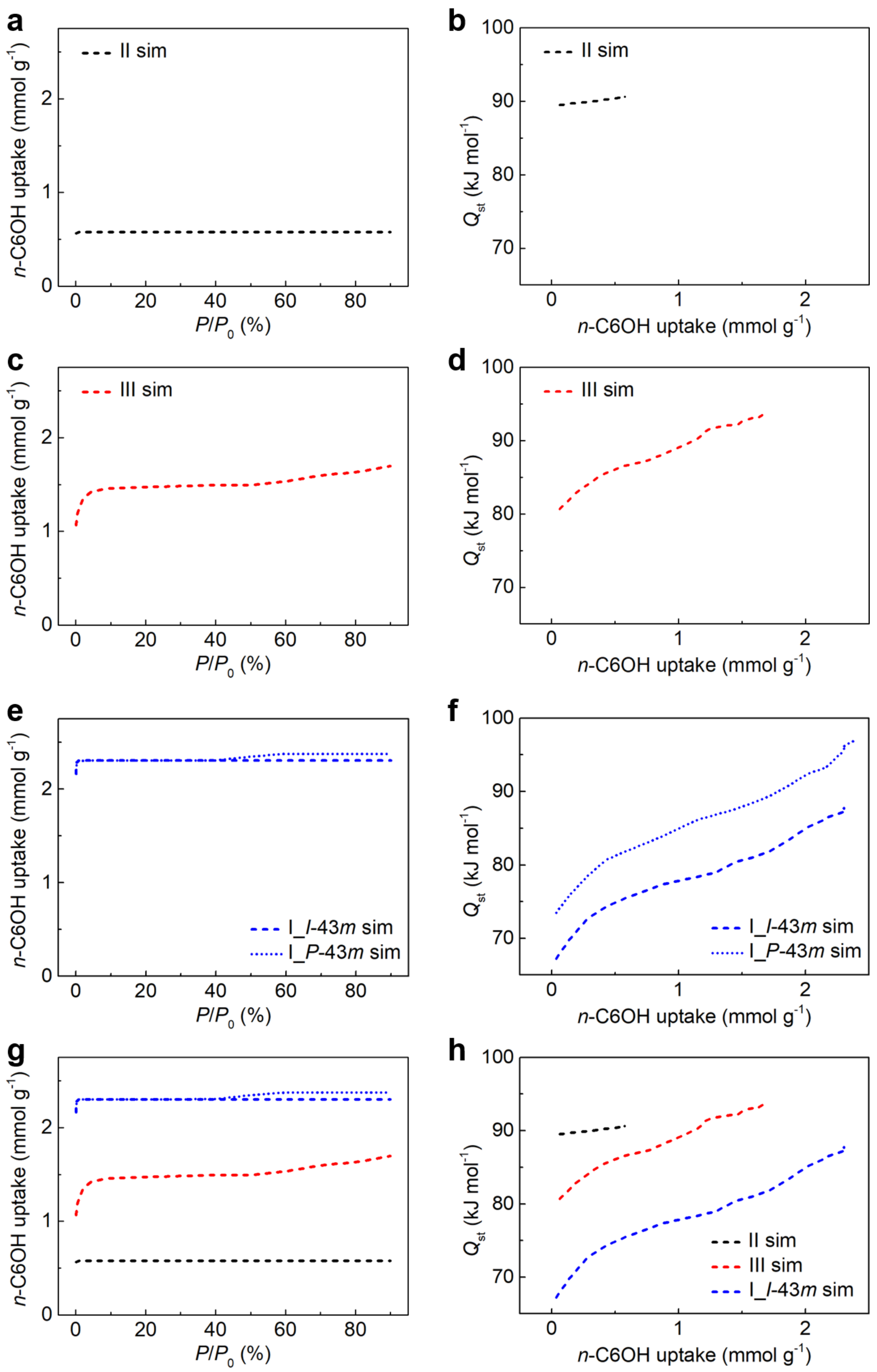

Supplementary Fig. 56 The simulated *n*-C6OH adsorption isotherms for ZIF-65(Zn) and corresponding guest adsorption heat at 298 K. The simulated isotherms of **a** II, **c** III, **e** I_*I*-43*m* and I_*P*-43*m*, and **g** all different phases. The simulated adsorption heats in **b** II, **d** III, **f** I_*I*-43*m* and I_*P*-43*m*, and **h** II, III, and I_*I*-43*m*. (Simulated result: dashed line.)

Supplementary Table 13 Comparison of the simulated and experimental uptake.

	Experimental data				Simulated result			
	**II**	**III**	**I_*I*-43*m***	**I_*P*-43*m***	**II**	**III**	**I_*I*-43*m***	**I_*P*-43*m***
N_{max} (mmol g^{-1})								
c-C6	0.85		3.83		0.58	1.15	2.88	3.38
n-C6	0.63[a]	2.04	3.09		0.58	1.73	2.38	2.88
EtOH	2.67[a]	4.46	6.68		2.30	4.41	5.55	6.68
i-C4OH	0.96[a]	2.24[a]	4.40		0.58	2.05	4.46	4.61
n-C6OH			2.94[b]		0.58	1.69	2.30	2.38
N_{max} (x·molecules per cell)								
c-C6	4		13		3	6	10	12
n-C6	3	11	11		3	9	8	10
EtOH	14	23	23		12	23	19	23
i-C4OH	5	12	15		3	11	16	16
n-C6OH			10		3	9	8	8
N_{max} (x·molecules per cage)								
c-C6	1 or 2		6 or 7		1	2	5	6
n-C6	1	3 or 4	5 or 6		1	3	4	5
EtOH	4 or 5	7 or 8	11 or 12		4	7 or 8	9 or 10	11 or 12
i-C4OH	1 or 2	4	7 or 8		1	3 or 4	8	8
n-C6OH			5		1	3	4	4

[a] Adsorption uptake reckoned by steps of the adsorption isotherm;

[b] Adsorption uptake is reckoned roughly by TG.

Note: max value refers to corresponding adsorption value at P/P_0 = 90%.

Supplementary Table 14 The simulated guest adsorption heat at infinite dilution and maximum uptake.

	Simulated result							
	**II**	**III**	**I_*I*-43*m***	**I_*P*-43*m***	**II**	**III**	**I_*I*-43*m***	**I_*P*-43*m***
	Q_{st}^{0} (kJ mol^{-1})				$Q_{\text{st}}^{\text{max}}$ (kJ mol^{-1})			
c-C6	56.7	43.7	44.9	52.4	58.1	51.8	60.4	65.7
n-C6	69.6	54.8	48.8	50.7	70.4	74.5	66.5	71.8
EtOH	52.6	48.7	44.0	56.1	87.6	78.7	74.2	77.1
i-C4OH	70.0	61.8	58.8	76.2	71.8	88.9	84.0	86.4
n-C6OH	89.5	80.7	67.2	73.5	90.7	93.9	87.7	97.4

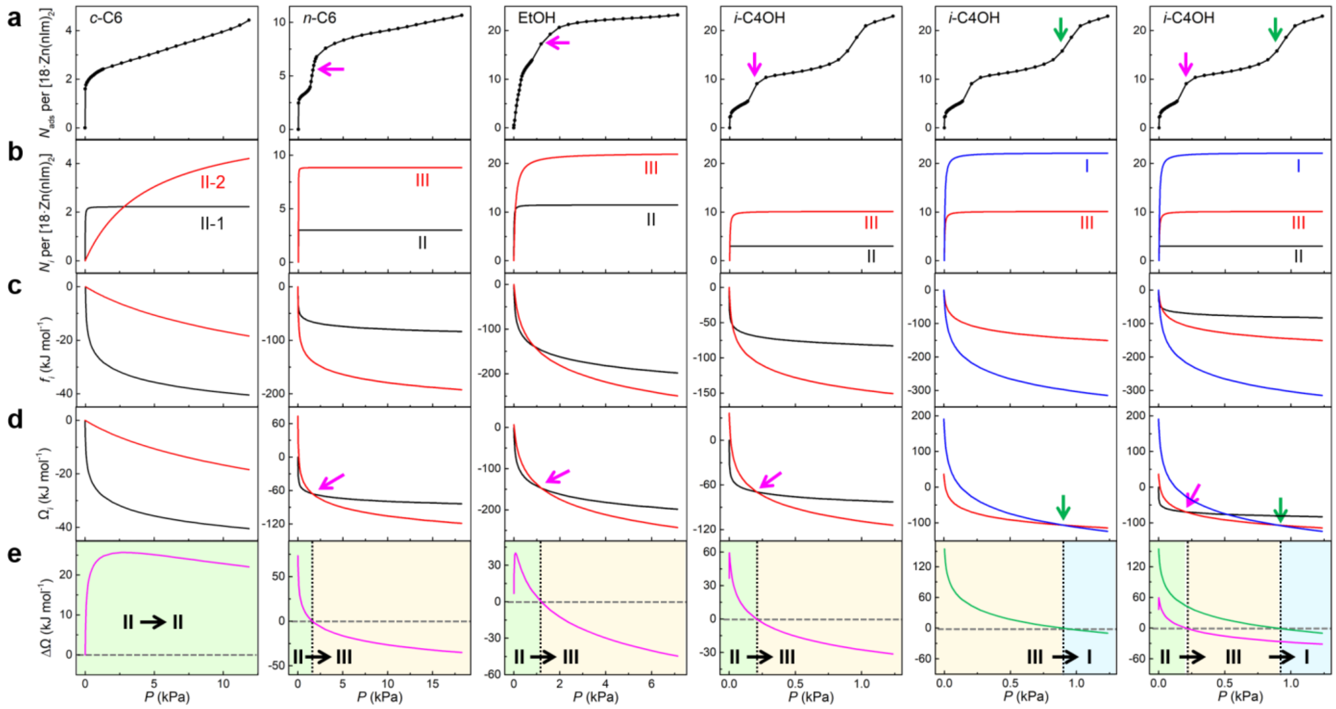

Supplementary Fig. 57 Osmotic ensemble model for predicting structural transitions of ZIF-65(Zn) induced by *c*-C6, *n*-C6, EtOH, and *i*-C4OH adsorption, respectively. a The experimental adsorption isotherms in ZIF-65(Zn)-II at 298 K. **b** Langmuir isotherms fitted on experimental or simulated adsorption in different ZIF-65(Zn) phases at 298 K (the adsorption isotherm of *c*-C6 is experimental, and these of other guests is simulated). **c** Plot the function of the pressure to calculate the free energy difference between two phases. **d** Osmotic potential for each phase. **e** The difference in osmotic potential ($\Delta\Omega$) between two phases. Note: $\Delta\Omega = 0$, which means that is the structural transition point.

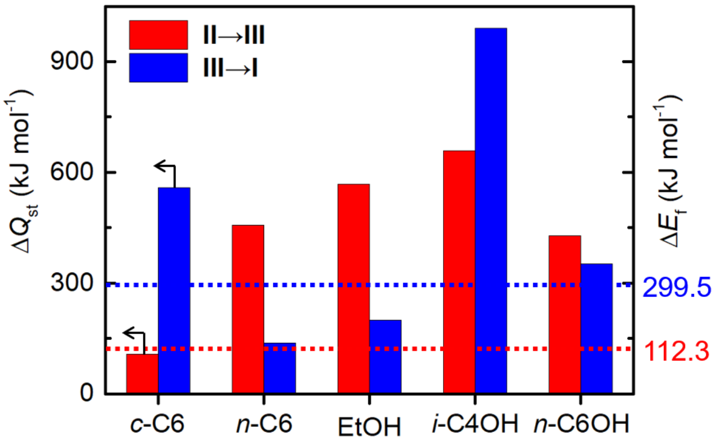

Supplementary Fig. 58 The comparison of host energy difference (ΔE_f) and guest adsorption heat difference (ΔQ_{st}) between different ZIF-65(Zn) phases. The ΔE_f is obtained by DFT calculations. For the empty ZIF-65(Zn) structure with 18·Zn(nIm)$_2$, ΔE_f(II→III) and ΔE_f(III→I) are around 112.3 and 299.5 kJ mol^{-1}, respectively. The ΔQ_{st}(II→III) and ΔQ_{st}(III→I) in the transition region are obtained from GCMC simulations. If the ΔQ_{st} is higher than the ΔE_f, the structural transition will occur; otherwise, no structural transition occurs.

Supplementary Table 15 The GCMC simulated uptake and corresponding guest adsorption heat at P/P_0=10% (adsorption saturation point in the low-pressure region), and guest adsorption heat difference (ΔQ_{st}) between different ZIF-65(Zn) phases.

	GCMC simulated result					
	**II**	**III**	**I_*I*-43*m***	**II**	**III**	**I_*I*-43*m***
	N (*x*·molecules per [18·Zn(nIm)$_2$])			*N* (mmol g^{-1})		
c-C6	3.0	5.5	14.2	0.58	1.06	2.73
n-C6	3.0	9.0	12.2	0.58	1.73	2.34
EtOH	11.6	20.2	24.9	2.22	3.88	4.78
i-C4OH	3.0	10.0	22.3	0.58	1.91	4.29
n-C6OH	3.0	7.6	12.0	0.58	1.46	2.30
	**II**	**III**	**I_*I*-43*m***	**II→III**	**III→I_*I*-43*m***	
	Q_{st} (kJ mol^{-1})			ΔQ_{st} (kJ mol^{-1})		
c-C6	58.1	51.2	59.3	108.0	558.6	
n-C6	70.5	74.5	66.3	457.6	137.6	
EtOH	87.6	78.3	71.5	568.7	200.1	
i-C4OH	71.8	87.7	83.5	658.5	989.9	
n-C6OH	90.7	92.2	87.7	428.1	352.3	

The guest adsorption heat difference equation (1): $\Delta Q_{st}(i \rightarrow j) = (N_j \times Q_{st\_}j) - (N_i \times Q_{st\_}i)$ (1)

The host energy difference equation (2): $\Delta E_f(i \rightarrow j) = E_f(j) - E_f(i)$ (2)

To assess the host energy difference (ΔE_f) between different ZIF-65(Zn) phases by DFT calculation, their structures need to contain the same number of atoms. Here, E_f(II) and E_f(III) are the energy of one unit cell, and E_f(I) is the energy of one unit cell multiplied by 1.5. Because one unit cell of phases II and III contains 18·Zn(nIm)$_2$, and the one unit cell of phase I contains 12·Zn(nIm)$_2$.

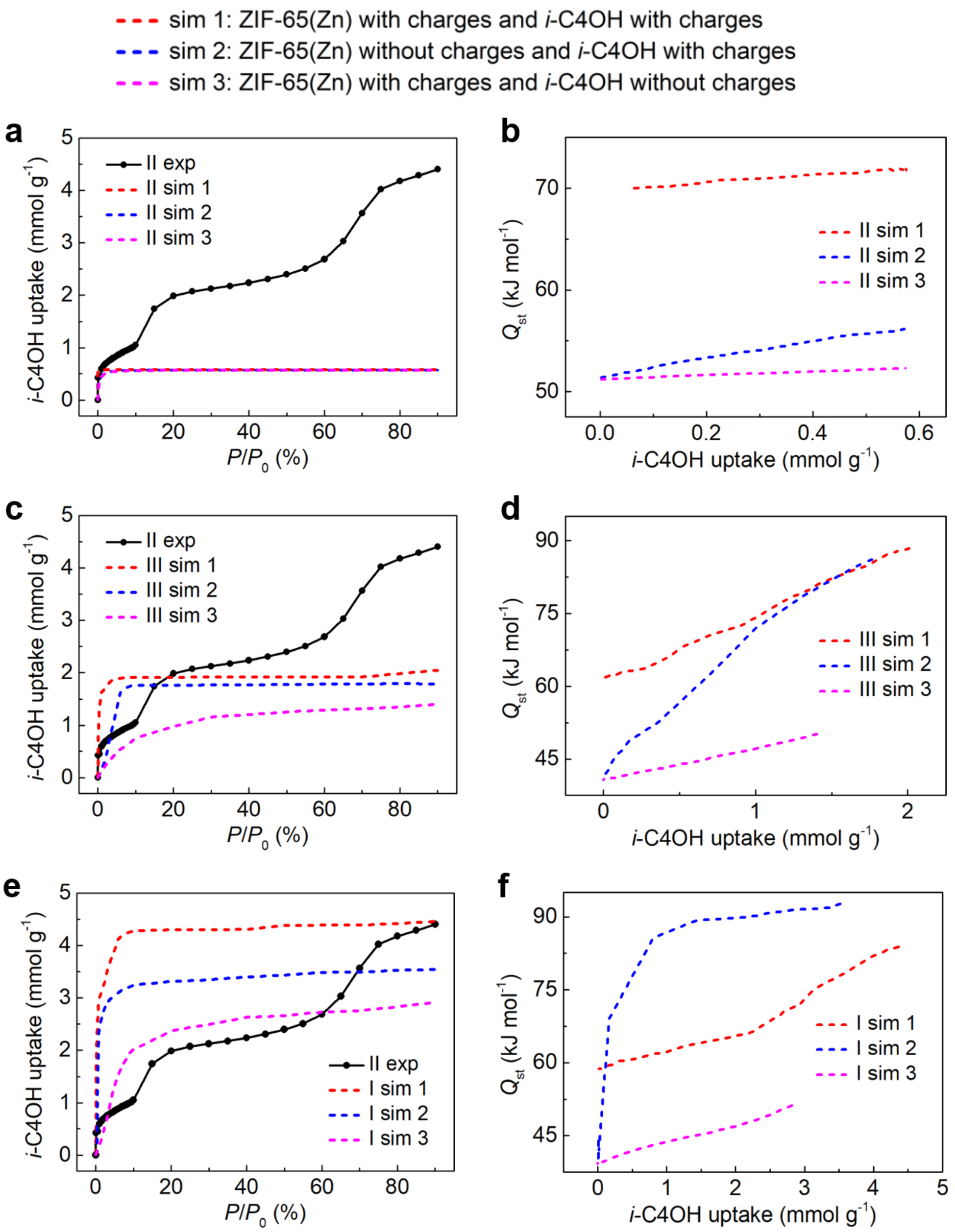

Supplementary Fig. 59 The effect of the structure and guest charges for the *i*-C4OH adsorption in ZIF-65(Zn). Comparison of the 3 different simulated adsorption isotherms of *i*-C4OH in **a** II, **c** III, and **e** I with the first, second, and third plateau on the experimental adsorption isotherm of ZIF-65(Zn)-II at 298 K, respectively. The corresponding 3 different simulated *i*-C4OH adsorption heat in **b** II, **d** III, and **f** I. (Experimental data: solid line; simulated result: dashed line.)

Supplementary Table 16 The simulated maximum uptake of the *i*-C4OH adsorption in ZIF-65(Zn) under 3 different simulations.

	**II**	**III**	**I_*I*-43*m***
		N_{max} (mmol g^{-1})	
**sim 1**	0.58	2.05	4.46
**sim 2**	0.58	1.79	3.55
**sim 3**	0.58	1.40	2.92

MC and DFTB calculated host–guest structures: most probable *n*-C6 or *i*-C4OH adsorption site in ZIF-65(Zn)-II, ZIF-65(Zn)-III and ZIF-65(Zn)-I with the maximum number of adsorbed molecules per cage. The host frameworks and guest molecules are shown as thin and thick stick models, respectively. Zn: slate gray; C: gray; N: blue; O: red; H: white. The strong guest–guest O–H⋯O H-bonding, weak host–guest C–H⋯O H-bonding, and guest–guest vdW interactions are displayed as red, black, and green dashed lines, respectively. [Note: *n*-hexane (*n*-C6), isobutanol (*i*-C4OH).]

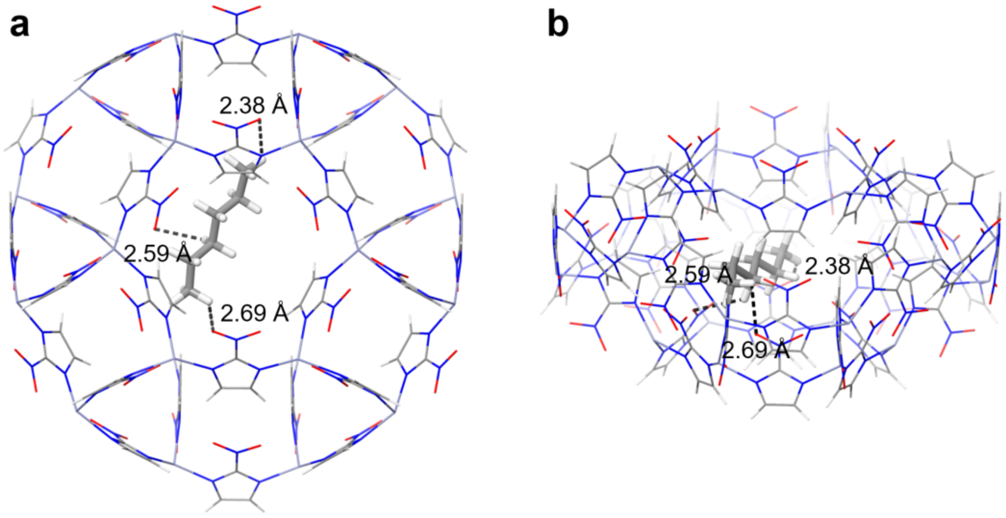

Supplementary Fig. 60 Most probable *n*-C6 adsorption site in ZIF-65(Zn)-II (1·*n*-C6 per cage). a The 6R-A is forward; **b** the 6R-B is forward.

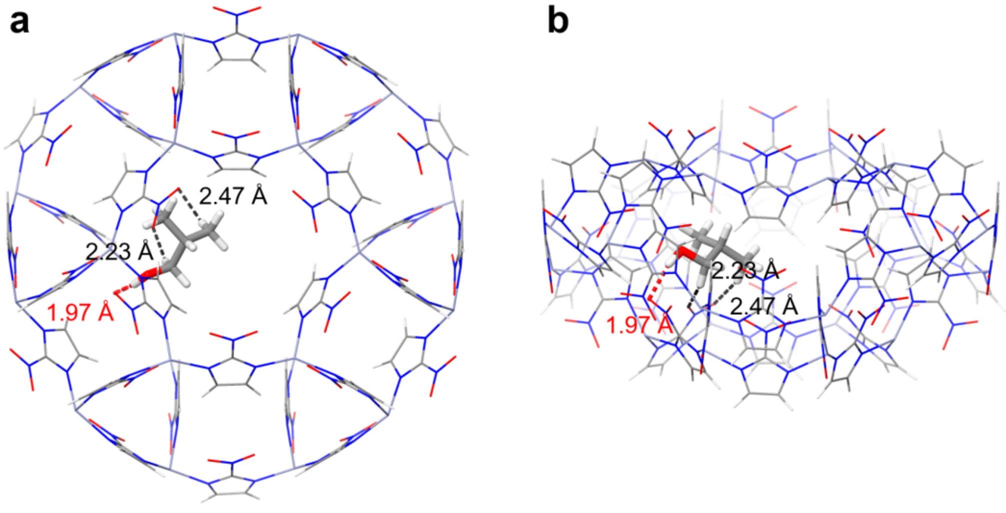

Supplementary Fig. 61 Most probable *i*-C4OH adsorption site in ZIF-65(Zn)-II (1·*i*-C4OH per cage). a The 6R-A is forward; **b** the 6R-B is forward.

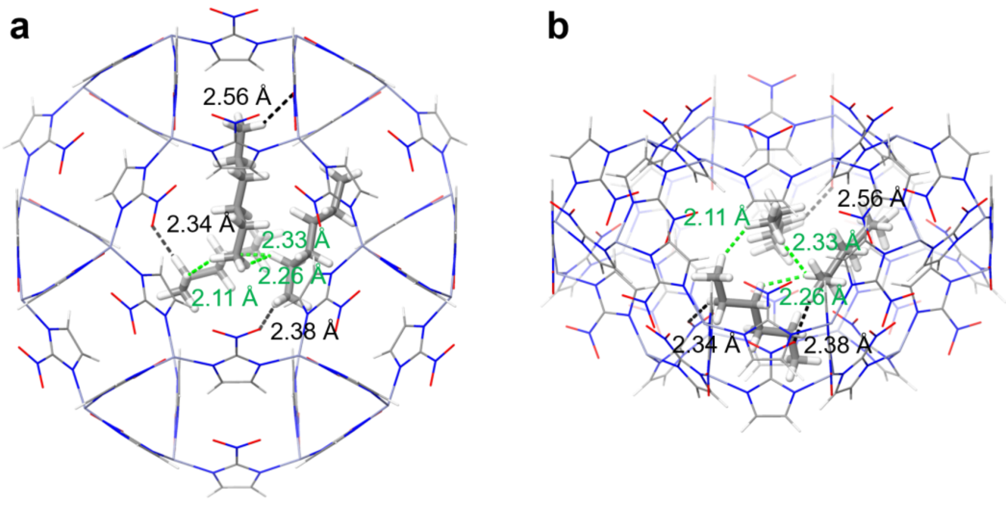

Supplementary Fig. 62 Most probable *n*-C6 adsorption site in ZIF-65(Zn)-III (3·*n*-C6 per cage). a The 6R-A is forward; **b** the 6R-B is forward.

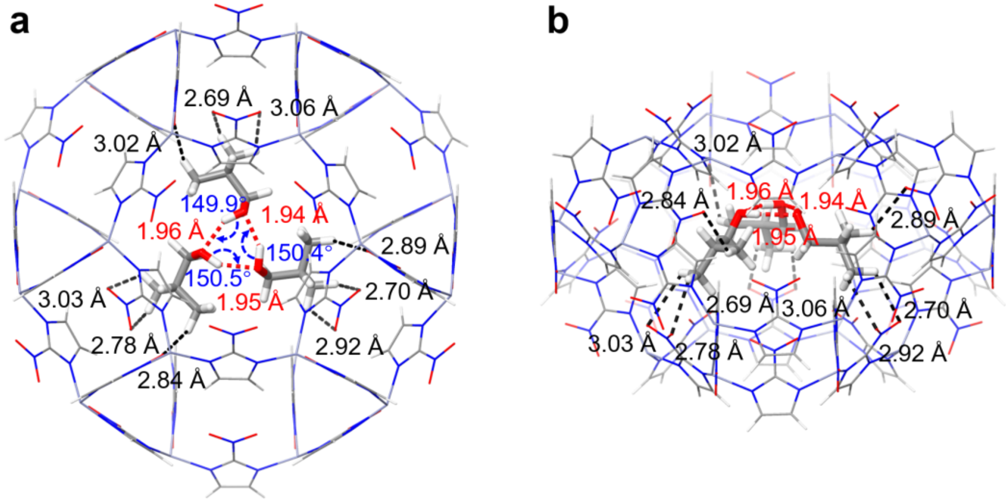

Supplementary Fig. 63 Most probable *i*-C4OH adsorption site in ZIF-65(Zn)-III (3·*i*-C4OH per cage). a The 6R-A is forward; **b** the 6R-B is forward.

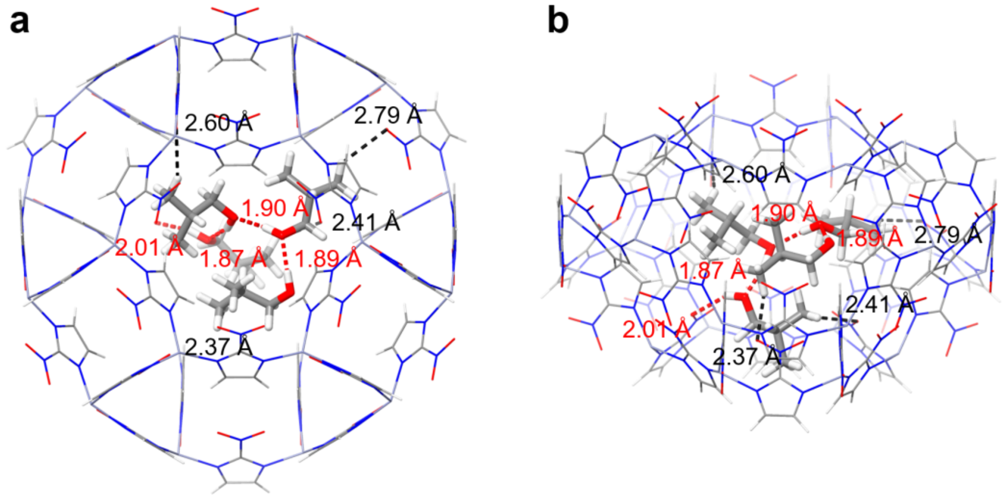

Supplementary Fig. 64 Most probable *i*-C4OH adsorption site in ZIF-65(Zn)-III (4·*i*-C4OH per cage). a The 6R-A is forward; **b** the 6R-B is forward.

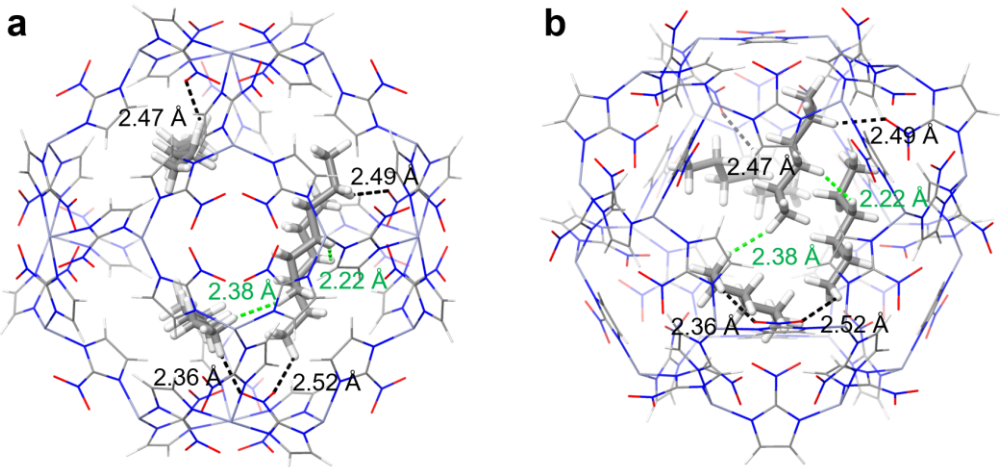

Supplementary Fig. 65 Most probable *n*-C6 adsorption site in ZIF-65(Zn)-I (4·*n*-C6 per cage). a The 6R-A is forward; **b** the 6R-B is forward.

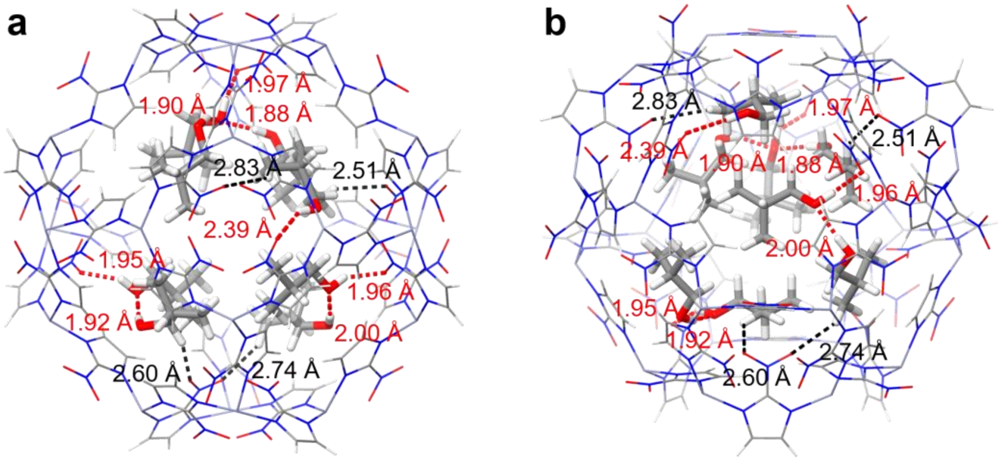

Supplementary Fig. 66 Most probable *i*-C4OH adsorption site in ZIF-65(Zn)-I (8·*i*-C4OH per cage). a The 6R-A is forward; **b** the 6R-B is forward.

Supplementary References

1. Banerjee, R. et al. High-throughput synthesis of zeolitic imidazolate frameworks and application to CO_2 capture. *Science* **319**, 939-943 (2008).
2. Dassault Systèmes BIOVIA. *Materials Studio Modeling Environment, Release 2017*. Dassault Systèmes BIOVIA, San Diego, CA, 2016.
3. Choi, Y., Noh, K., Lee, J. & Kim, J. Porosity properties of the conformers of sodalite-like zeolitic imidazolate frameworks. *J. Am. Chem. Soc.* **140**, 14586-14589 (2018).
4. Martin, M. G. & Siepmann, J. I. Transferable potentials for phase equilibria. 1. united-atom description of *n*-alkanes. *J. Phys. Chem. B* **102**, 2569-2577 (1998).
5. Chen, B., Potoff, J. J. & Siepmann, J. I. Monte carlo calculations for alcohols and their mixtures with alkanes. transferable potentials for phase equilibria. 5. united-atom description of primary, secondary, and tertiary alcohols. *J. Phys. Chem. B* **105**, 3093-3104 (2001).
6. Keasler, S. J., Charan, S. M., Wick, C. D., Economou, I. G. & Siepmann, J. I. Transferable potentials for phase equilibria-united atom description of five- and six-membered cyclic alkanes and ethers. *J. Phys. Chem. B* **116**, 11234-11246 (2012).
7. Mayo, S. L., Olafson, B. D. & Goddard, W. A. Dreiding: a generic force field for molecular simulations. *J. Phys. Chem.* **94**, 8897-8909 (1990).
8. Amrouche, H. et al. Experimental and computational study of functionality impact on sodalite–zeolitic imidazolate frameworks for CO_2 separation. *J. Phys. Chem. C* **115**, 16425-16432 (2011).
9. Gao, M., Wang, J., Rong, Z., Shi, Q. & Dong, J. A combined experimental-computational investigation on water adsorption in various ZIFs with the SOD and RHO topologies. *RSC Adv.* **8**, 39627-39634 (2018).
10. Cui, Q., Elstner, M., Kaxiras, E., Frauenheim, T. & Karplus, M. A QM/MM implementation of the self-consistent charge density functional tight binding (SCC-DFTB) method. *J. Phys. Chem. B* **105**, 569-585 (2001).
11. Aradi, B., Hourahine, B. & Frauenheim, T. DFTB+, a sparse matrix-based implementation of the DFTB method. *J. Phys. Chem. A* **111**, 5678-5684 (2007).
12. Official internet link to the program DFTB+: http://www.dftb-plus.info/.
13. Gaus, M., Lu, X., Elstner, M. & Cui, Q. Parameterization of DFTB3/3OB for sulfur and phosphorus for chemical and biological applications. *J. Chem. Theory Comput.* **10**, 1518-1537 (2014).
14. Han, X. et al. Visualizing the {110} surface structure of equilibrium-form ZIF-8 crystals by low-dose Cs-corrected TEM. *Nanoscale* **13**, 13215-13219 (2021).
15. Grimme, S., Antony, J., Ehrlich, S. & Krieg, H. A consistent and accurate ab initio parametrization of density functional dispersion correction (DFT-D) for the 94 elements H-Pu. *J. Chem. Phys.* **132**, 154104 (2010).
16. Grimme, S., Ehrlich, S. & Goerigk, L. Effect of the damping function in dispersion corrected density functional theory. *J. Comput. Chem.* **32**, 1456-1465 (2011).
17. Tu, M., Wiktor, C., Röslera, C. & Fischer, R. A. Rapid room temperature syntheses of zeolitic-imidazolate framework (ZIF) nanocrystals. *Chem. Commun.* **50**, 13258-13260 (2014).
18. Tu, M., Wannapaiboon, S., Khaletskaya, K. & Fischer, R. A. Engineering zeolitic-imidazolate framework (ZIF) thin film devices for selective detection of volatile organic compounds. *Adv. Funct. Mater.* **25**, 4470-4479 (2015).
19. Bhattacharyya, S. et al. Acid gas stability of zeolitic imidazolate frameworks: generalized kinetic and thermodynamic characteristics. *Chem. Mater.* **30**, 4089-4101 (2018).
20. Zhao, P. et al. Phase transitions in zeolitic imidazolate framework 7: the importance of framework flexibility and guest-induced instability. *Chem. Mater.* **26**, 1767-1769 (2014).
21. Klein, R. A. et al. Structural resolution and mechanistic insight into hydrogen adsorption in flexible ZIF-7. *Chem. Sci.* **12**, 15620-15631 (2021).
22. Park, K. S. et al. Exceptional chemical and thermal stability of zeolitic imidazolate frameworks. *Proc. Natl. Acad. Sci. U. S. A.* **103**, 10186-10191 (2006).
23. Knebel, A. et al. Defibrillation of soft porous metal-organic frameworks with electric fields. *Science* **358**, 347-351 (2017).
24. Moggach, S. A., Bennett, T. D. & Cheetham, A. K. The effect of pressure on ZIF-8: increasing pore size with pressure and the formation of a high-pressure phase at 1.47 GPa. *Angew. Chem. Int. Ed.* **48**, 7087-7089 (2009).
